# Transcriptional Repression of Aerobic Glycolysis by OVOL2 in Breast Cancer

**DOI:** 10.1002/advs.202200705

**Published:** 2022-07-27

**Authors:** Xiujuan Zhang, Fei Luo, Shaliu Luo, Ling Li, Xinxin Ren, Jing Lin, Yingchun Liang, Chao Ma, Lihua Ding, Deyu Zhang, Tianxing Ye, Yanni Lin, Bilian Jin, Shan Gao, Qinong Ye

**Affiliations:** ^1^ Department of Medical Molecular Biology Beijing Institute of Biotechnology Collaborative Innovation Center for Cancer Medicine Beijing 100850 China; ^2^ Medical School of Guizhou University Guiyang 550025 China; ^3^ Department of Clinical Laboratory The Fourth Medical Center of PLA General Hospital Beijing 100037 China; ^4^ Shanxi Medical University Taiyuan 030000 China; ^5^ Institute of Cancer Stem Cell Dalian Medical University Dalian 116000 China; ^6^ Zhongda Hospital School of Life Sciences and Technology Advanced Institute for Life and Health Southeast University Nanjing 210096 China

**Keywords:** aerobic glycolysis, OVOL2, transcriptional regulation, tumor growth and metastasis

## Abstract

Aerobic glycolysis (Warburg effect), a hallmark of cancer, plays a critical role in cancer cell growth and metastasis; however, direct inhibition of the Warburg effect remains largely unknown. Herein, the transcription factor OVO‐like zinc finger 2 (OVOL2) is demonstrated to directly repress the expression of several glycolytic genes, blocking the Warburg effect and breast tumor growth and metastasis in vitro and in vivo. OVOL2 inhibits glycolysis by recruiting the nuclear receptor co‐repressor (NCoR) and histone deacetylase 3 (HDAC3). The tumor suppressor p53, a key regulator of cancer metabolism, activates OVOL2 by binding to the oncoprotein mouse double minute 2 homolog (MDM2) and inhibiting MDM2‐mediated ubiquitination and degradation of OVOL2. OVOL2 expression is negatively correlated with glycolytic gene expression and can be a good predictor of prognosis in patients with breast cancer. Therefore, targeting the p53/MDM2/OVOL2 axis provides a potential avenue for cancer treatment, especially breast cancer.

## Graphical abstract

OVOL2 inhibits the transcription of glycolytic genes (e.g., mouse double minute 2 homolog, HK2) by recruiting NCoR and HDAC3. OVOL2 can be repressed by MDM2‐mediated ubiquitination and degradation and induced under hypoxia by p53, which binds MDM2 and blocks the MDM2–OVOL2 interaction. Thus, the p53/MDM2/OVOL2 axis links glycolytic gene expression to glycolysis and tumor growth and metastasis.

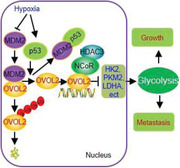

## 1 Introduction

Aerobic glycolysis (the Warburg effect), a hallmark of cancer, is caused by the active metabolic reprogramming required to support sustained cancer cell proliferation and malignant progression.^[^ ^1^, ^2^, ^3^ ^]^ Compared with normal cells, cancer cells frequently demonstrate elevated glucose uptake and glycolysis, despite abundant oxygen, thus generating increased lactate levels. Furthermore, agents targeting aerobic glycolysis have shown promising anticancer activity both in vitro and in vivo.^[^ ^4^, ^5^ ^]^ Aerobic glycolysis is directly regulated by transcription factors, such as hypoxia‐inducible factor‐1*α* (HIF‐1*α*),^[^ ^6^ ^]^ oncogene c‐Myc,^[^ ^7^ ^]^ sine oculis homeobox 1 (SIX1),^[^ ^8^ ^]^ Forkhead Box K1 and K2 (FOXK1 and FOXK2),^[^ ^9^ ^]^ and tumor suppressor p53.^[^ ^10^ ^]^ HIF‐1*α*, c‐Myc, SIX1 and FOXK1/2 can directly activate the expression of glycolytic genes by binding to glycolytic gene promoters, resulting in enhanced aerobic glycolysis. Conversely, the tumor‐suppressive transcription factor p53 directly suppresses glycolytic gene transcription by binding to glycolytic gene promoters, thereby reducing aerobic glycolysis. Despite extensive studies on mechanisms underlying the transcriptional activation of the Warburg effect, the molecular machinery involved in the transcriptional inhibition of this effect remains largely elusive.

OVO‐like zinc finger 2 (OVOL2), a member of the OVO family of conserved zinc‐finger transcription factors, can regulate embryonic development and cancer metastasis.^[^ ^11^, ^12^, ^13^, ^14^, ^15^, ^16^, ^17^ ^]^ It has been reported that Ovol2 gene knockout (KO) in mice results in embryonic lethality before embryonic day 10.5, indicating that OVOL2 is involved in early embryonic development.^[^ ^11^ ^]^ OVOL2 expression was shown to be downregulated in hepatocellular carcinoma and colorectal cancer.^[^ ^13^, ^14^ ^]^ Decreased OVOL2 expression predicts poor clinical outcomes. OVOL2 can inhibit cancer cell migration, invasion, and metastasis. However, whether OVOL2 regulates cancer metabolism remains unclear. In the present study, we identified OVOL2 as a key transcriptional repressor of aerobic glycolysis.

## 2 Results

### 2.1 Identification of OVOL2 as a Key Inhibitor of Glycolytic Gene Expression and Glycolysis

**Figure 1:**
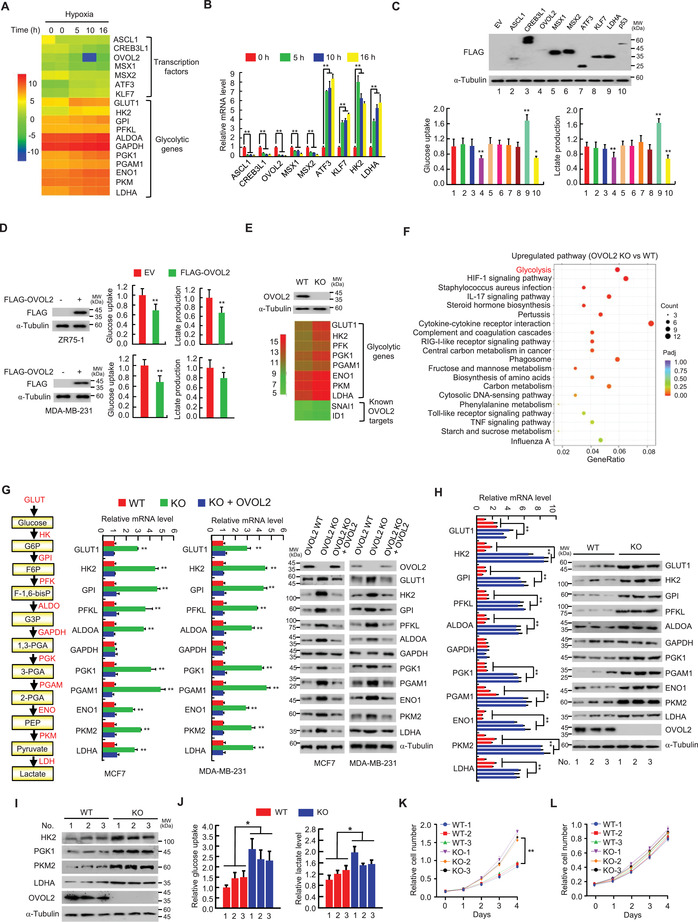
OVOL2 inhibits glycolytic gene expression and glycolysis. A) Heatmap of transcription factors and known hypoxia‐inducible glycolytic genes identified by RNA‐seq using MCF7 cells treated with hypoxia (1% O_2_) for indicated times. B) RT‐qPCR analysis of transcription factors and some known hypoxia‐inducible glycolytic genes identified in (A) in MCF7 cells treated with hypoxia for indicated times. C) Glucose uptake and lactate production in MCF7 cells transfected with the indicated FLAG‐tagged expression vectors or empty vector (EV). LDHA was used as a positive regulator of glucose uptake and lactate production, and p53 as a negative regulator of glucose uptake and lactate production. Typical immunoblot shows the expression of FLAG‐tagged proteins. *α*‐Tubulin was used as a loading control. D) Measurement of glucose uptake and lactate production in ZR75‐1 and MDA‐MB231 cells transfected with FLAG‐tagged OVOL2 (FLAG‐OVOL2) or EV. Representative immunoblot shows FLAG‐OVOL2 expression. E) Heatmap of glycolytic genes and known OVOL2 downstream target genes identified by RNA‐seq using OVOL2 wild‐type (WT) or knockout (KO) MCF7 cells. F) KEGG pathway analysis of genes differentially expressed between OVOL2 WT and KO MCF7 cells as in (E). G) RT‐qPCR and immunoblot analysis of glycolytic gene expression in OVOL2 WT or KO MCF7 or MDA‐MB‐231 cells. To avoid potential off‐target effects of KO, the KO cells were stably infected with lentivirus carrying OVOL2. *α*‐Tubulin was used as a loading control for analyzing glycolytic gene expression. A schematic diagram of the aerobic glycolysis pathway is indicated on the left. H) RT‐qPCR and immunoblot analysis of glycolytic gene expression in OVOL2 WT or KO MEFs isolated from corresponding mouse. WT mice were littermates of the KO mice (*n* = 3). I) Representative immunoblot analysis of glycolytic gene expression in OVOL2 WT and KO mouse mammary tissues isolated from mammary‐specific OVOL2 KO mice generated by mating conditional OVOL2 KO mice with MMTV‐Cre transgenic mice. J) Measurement of glucose uptake and lactate production in OVOL2 WT or KO MEFs from (H). K,L) Cell proliferation curves of OVOL2 WT and KO MEFs (*n* = 3) cultured in media containing 25 mm glucose (K) or 10 mm galactose (L). Data shown are mean ± standard deviation (SD) of triplicate measurements that have been repeated 3 times with similar results (B,C,G,H,K,L). Data shown are mean ± SD of quintuplicate measurements that have been repeated 3 times with similar results (D,J). Two‐sided Student's *t*‐test was used to compare the means of two groups. When more than two groups were compared, one‐way ANOVA was performed. *p < 0.05, **p < 0.01 versus control cells or EV.

As hypoxia is a key phenomenon in cancers,^[^ ^18^, ^19^, ^20^ ^]^ and some known transcription factors critical for the Warburg effect, such as c‐Myc and SIX1, are modulated by hypoxia at the mRNA level,^[^ ^8^, ^21^ ^]^ we used RNA sequencing (RNA‐seq) to identify transcription factors with unknown functions related to the inhibition of the Warburg effect in MCF7 human breast cancer cells under hypoxia or normoxia. We selected breast cancer cells for screening experiments, given that previous research has demonstrated that these cells exhibit the Warburg effect. As expected, hypoxia could regulate expression of several previously reported genes, including glycolysis‐related genes (**Figure** **1**A). Importantly, we identified several hypoxia‐regulated transcription factors with unknown functions related to aerobic glycolysis (accession number GEO: GSE166203). Quantitative reverse transcription‐PCR (RT‐qPCR) confirmed the altered expression of these transcription factors (Figure 1B). The difference in the altered range between RNA‐seq and RT‐qPCR results may be attributed to the distinct complexities of the RNA/cDNA library and the sensitivity of the two methods. Cancer cells aggressively consume glucose and exhibit high rates of lactate production. Among these transcription factors (e.g., CREB3L1), only OVOL2 significantly inhibited glucose uptake and lactate production, similar to a previously reported p53 tumor suppressor (Figure 1C). It should be noted that, most recently, CREB3L1, along with the transcription factors EGR2 and SOX4, has been reported to induce glycolysis in inflammatory cancer‐associated fibroblasts.^[^ ^22^ ^]^ However, the study conclusion was based on bioinformatics analysis only, with no experimental data supporting the conclusion. The observation that the molecular weight of OVOL2 was higher than expected might be due to post‐translational modifications of OVOL2, such as phosphorylation and/or other modifications, given that DNA sequences encoding OVOL2 were accurate. Overexpression of OVOL2, but not of other OVO family members (OVOL1 and OVOL3), reduced glucose uptake and lactate production in MCF7 cells (Figure S1A, Supporting Information). Moreover, OVOL2 overexpression repressed glucose uptake and lactate production in ZR75‐1 and MDA‐MB‐231 breast cancer cells (Figure 1D). Thus, we selected OVOL2 to identify its downstream effectors by performing RNA‐seq using the OVOL2 knockout (KO) MCF7 cell line or control cell line. Indeed, OVOL2 regulated the transcription of eight glycolysis‐related genes, in addition to the previously reported OVOL2‐regulated genes (accession number GEO: GSE189947) (Figure 1E). The glycolytic pathway was enriched based on the Kyoto Encyclopedia of Genes and Genomes (KEGG) analysis (Figure 1F). RT‐qPCR confirmed the altered expression of all glycolysis‐related genes (GLUT1, HK2, PFKL, PGK1, PGAM1, ENO1, PKM2, and LDHA) in OVOL2 KO MCF7 and MDA‐MB‐231 cells (Figure 1G). In addition, we examined the effect of OVOL2 KO on the transcription of three glycolysis‐related genes (GPI, ALDOA, and GAPDH), which were not identified by RNA‐seq. OVOL2 KO increased the transcription of GPI and ALDOA but not GAPDH. As mentioned above, the discrepancy between the RNA‐seq and RT‐qPCR results may be due to the different complexities of the RNA/cDNA library and the sensitivity of the two methods. Consistent with the results of OVOL2 modulation of glycolytic gene transcription, OVOL2 KO MCF7 and MDA‐MB‐231 cells showed enhanced expression of GLUT1, HK2, GPI, PFKL, ALDOA, PGK1, PGAM1, ENO1, PKM2 and LDHA proteins but not GAPDH protein (Figure 1G). Furthermore, the effect of OVOL2 KO on glycolytic gene expression was rescued by OVOL2 re‐expression in OVOL2 KO MCF7 and MDA‐MB‐231 cells. OVOL2‐mediated inhibition of glycolytic gene expression was not dependent on p53, a well‐known inhibitor of glycolysis, given that p53 KO in HCT116 human colon cancer cells or p53 knockdown (KD) in MCF7 cells did not affect the repression of glycolytic gene expression by OVOL2 overexpression (Figure S1B,C, Supporting Information). As OVOL2 KO in mice resulted in early embryonic lethality in the present, as well as in a previous study,^[^ ^11^ ^]^ we prepared OVOL2 KO mouse embryonic fibroblasts (MEFs) by adding Cre recombinase adenovirus (Ad‐Cre) to MEFs isolated from conditional OVOL2^fl/fl^ mice. Similar to OVOL2 KO cancer cells, OVOL2 KO MEFs exhibited enhanced glycolytic gene expression (Figure 1H). Increased glycolytic gene expression was also observed in mammary tissue‐specific OVOL2^fl/fl^ mice (Figure 1I). Moreover, OVOL2 KO MEFs demonstrated increased lactate and ATP production (Figure 1J). MEFs use glucose or galactose to generate energy via glycolysis or oxidative phosphorylation (OXPHOS). Cells grown in galactose mostly rely on OXPHOS for energy production.^[^ ^23^ ^]^ On culturing cells in glucose‐containing media, OVOL2 KO MEFs grew significantly faster than wild‐type (WT) MEFs (Figure 1K). However, when cells were grown in galactose‐containing media, WT and KO MEFs grew at similar rates (Figure 1L). These results indicated that increased glycolysis by OVOL2 KO supports cell proliferation. Overall, these data strongly suggested that OVOL2 is a key inhibitor of glycolytic gene expression and glycolysis.

### 2.2 OVOL2 Represses Glycolytic Gene Promoter Activity by Binding OVOL2‐Responsive Elements

**Figure 2:**
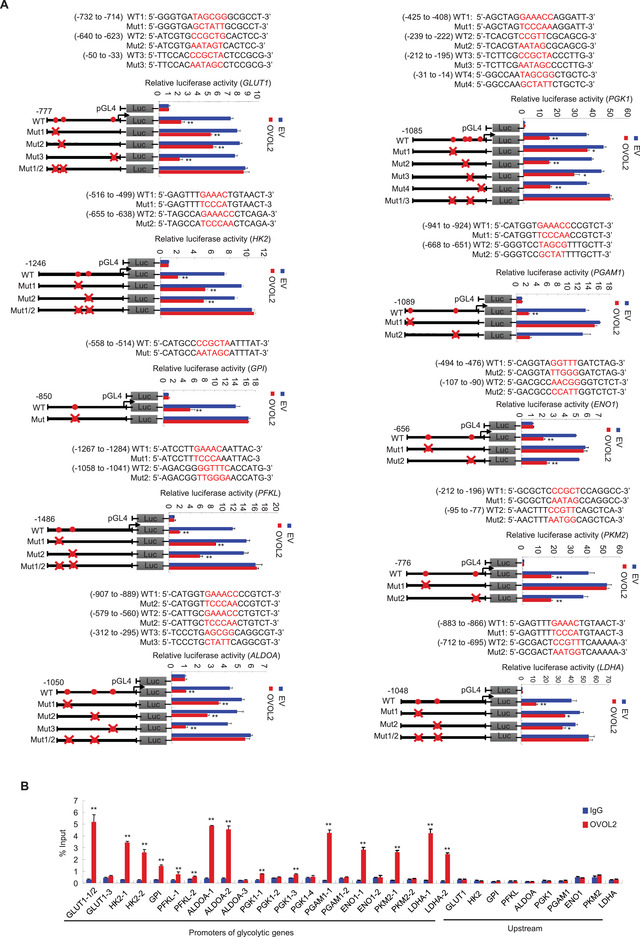
OVOL2 binds the OVOL2‐responsive element to inhibit glycolytic gene promoter activity. A) Luciferase activity of different glycolytic gene promoter reporters in MDA‐MB‐231 cells transfected with MYC‐tagged OVOL2 or EV. Filled circles indicate the position of putative OVOL2‐binding sites, and ‘‘ × ’’ indicates the mutated OVOL2‐binding sites. Red letters indicate the putative or mutated OVOL2‐binding sequences. WT, wild‐type; Mut, mutant. B) ChIP analysis of OVOL2 occupancy on promoters of glycolytic genes in MDA‐MB‐231 cells. IgG (immunoglobulin G): normal serum. The different number after each gene represents regions containing different putative OVOL2‐binding sites from left to right, as shown in (A). Data shown are mean ± SD of triplicate measurements that have been repeated 3 times with similar results. Data were analyzed using a two‐tailed Student's *t*‐test. *p < 0.05, **p < 0.01 versus respective promoter reporter without OVOL2 (A). **p < 0.01 versus respective normal IgG (B).

Genome‐wide analyses of OVOL2 binding sites in human corneal epithelial cells and mouse mammary epithelial cells with stem/progenitor cell features using chromatin immunoprecipitation sequencing (ChIP‐seq) show that OVOl2 binds four glycolytic genes (GLUT1, GPI, PFKL, and ENO1);^[^ ^12^, ^24^ ^]^ however, these high‐throughput sequencing results were not experimentally confirmed. OVOL2 reportedly binds chromatin primarily through the consensus sequence, CCGTTA or CCGCTA. OVOL2 may also bind to chromatin through GAAACC or GGTTTC. To determine whether OVOL2 modulates glycolytic gene transcription, we searched up to ≈1.5 kb promoter regions of these four glycolytic genes and the other six glycolytic genes identified in the present study for putative OVOL2 binding sites and generated promoter reporters containing the putative binding sites (**Figure** **2**A and Figure S2A, Supporting Information). OVOL2 overexpression decreased the reporter activity of GLUT1, HK2, GPI, PFKL, ALDOA, PGK1, PGAM1, ENO1, PKM2, and LDHA. The GPI, PGAM1, ENO1 and PKM2 promoters contained one OVOL2 binding site because mutation of the corresponding binding site, but not other putative OVOL2 binding sites, abrogated OVOL2‐mediated repression of promoter‐driven reporter activity. For promoters with two OVOL2 binding sites (GLUT1, HK2, PFKL, ALDOA, PGK1, and LDHA promoters), mutation of one of these binding sites only attenuated OVOL2‐mediated repression of promoter‐driven reporter activity. Mutation of these two binding sites, but not other putative OVOL2 binding sites, abolished OVOL2‐mediated suppression of promoter‐driven reporter activity. ChIP analysis demonstrated that endogenous OVOL2 was recruited to regions containing binding sites whose mutations attenuated or abrogated OVOL2‐mediated inhibition of the promoter reporter activity, but not to binding sites whose mutation did not alter promoter‐driven reporter activity or regions upstream of the promoters (Figure 2B and Figure S2B, Supporting Information). These data indicated that OVOL2 inhibits glycolytic gene transcription by binding to glycolytic gene promoters.

### 2.3 OVOL2 Inhibits Glycolytic Gene Expression by Interacting with the NCoR/HDAC3 Co‐Repressor Complex

**Figure 3:**
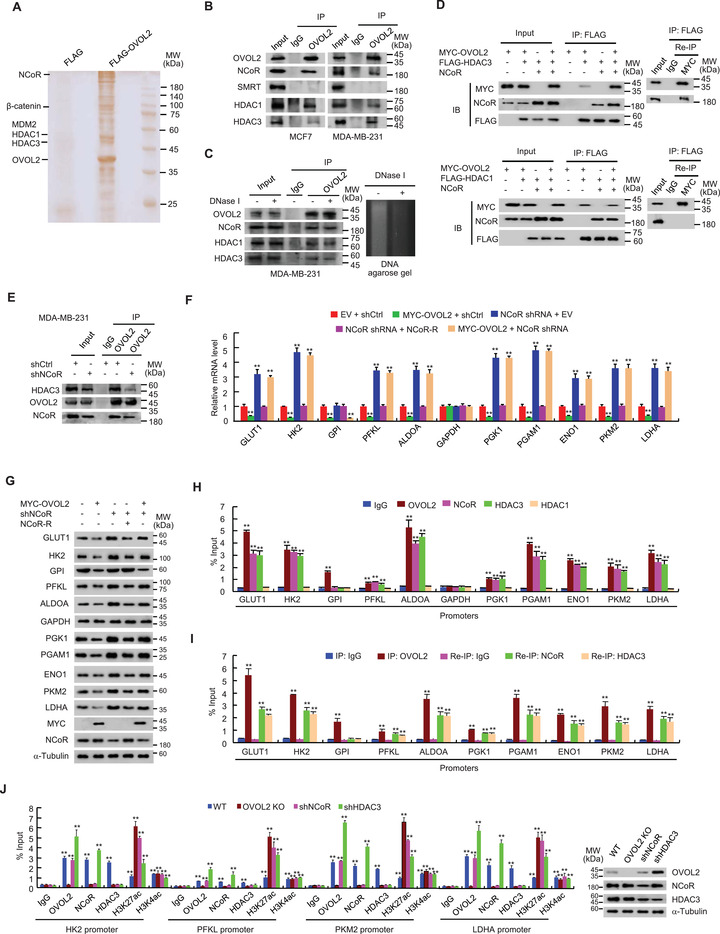
OVOL2 inhibits glycolytic gene expression by interacting with the NCoR/HDAC3 co‐repressor complex. A) Cellular lysates from MCF7 cells stably expressing FLAG (control) or FLAG‐OVOL2 were purified with anti‐FLAG affinity columns and eluted with FLAG peptide. The eluates were resolved by SDS‐PAGE and stained with silver. The differential protein bands were retrieved and analyzed by mass spectrometry. B) MCF7 or MDA‐MB‐231 cells were immunoprecipitated with anti‐OVOL2 or normal IgG, and precipitates were analyzed by immunoblotting (IB) with indicated antibodies. IP, immunoprecipitation. C) Co‐IP analysis of MDA‐MB‐231 cells treated with or without DNase I. DNA agarose gel electrophoresis serves as a control for DNase I activity. D) MDA‐MB‐231 cells transfected with indicated plasmids were immunoprecipitated with anti‐FLAG agarose beads. The immune complexes were eluted with FLAG peptide and reimmunoprecipitated (Re‐IP) with anti‐MYC or normal IgG. The resulting precipitates were analyzed by IB with indicated antibodies. E) MDA‐MB‐231 cells stably infected with lentivirus carrying NCoR short hairpin RNA (shRNA) or control shRNA (shNCoR or shCtrl) cells were immunoprecipitated with anti‐OVOL2 or normal IgG, and precipitates were analyzed by IB with indicated antibodies. F,G) MDA‐MB‐231 cells stably infected with lentivirus carrying shNCoR or shCtrl were stably transfected with MYC‐tagged OVOL2 or shRNA‐resistant NCoR (NCoR‐R) or EV as indicated. Glycolytic gene expression was examined using RT‐qPCR (F) and IB (G). H) ChIP analysis of OVOL2, NCoR, and HDAC1/3 occupancy on glycolytic gene promoters in MDA‐MB‐231 cells. Promoter regions of each gene represent the region containing the first OVOL2 binding site shown in Figure 2B unless only one OVOL2 binding site is present within the gene promoter analyzed. I) Re‐ChIP analysis of the occupancy of OVOL2 and NCoR or HDAC3 on the indicated glycolytic gene promoters in MDA‐MB‐231 cells. Re‐ChIP was performed after ChIP with anti‐OVOL2. J) ChIP analysis of OVOL2, NCoR, HDAC3, and histone H3 acetylation (H3K27ac and H3K4ac) occupancy on indicated glycolytic gene promoters in OVOL2 WT or KO MDA‐MB‐231 cells or MDA‐MB‐231 cells stably infected with lentivirus carrying shNCoR or HDAC3 shRNA (shHDAC3). Representative immunoblot shows the expression of OVOL2, NCoR and HDAC3. Data shown are mean ± SD of triplicate measurements that have been repeated 3 times with similar results. Statistical significance was assessed by a two‐tailed Student's *t*‐test. **p < 0.01 versus respective MDA‐MB‐231 cells transfected with EV and shCtrl (F). **p < 0.01 versus respective normal IgG (H–J).

Transcription factors can inhibit gene transcription by interacting with transcriptional co‐repressors to subsequently recruit histone‐modifying enzymes, such as histone deacetylases (HDACs).^[^ ^25^, ^26^ ^]^ To gain mechanistic insight into OVOL2 inhibition of glycolytic gene transcription, we used co‐immunoprecipitation (Co‐IP) combined with mass spectrometry to identify its interactome. In addition to the previously reported OVOL2‐interacting proteins *β*‐catenin and HDAC1, we only identified NCoR (nuclear receptor co‐repressor) and HDAC3 as a transcriptional co‐repressor and a histone deacetylase, respectively, and the mouse double minute 2 homolog (MDM2) oncoprotein as an E3 ubiquitin ligase (see below for regulation of OVOL2 expression) (**Figure** **3**A and Table S1, Supporting Information). Co‐IP of endogenous or exogenous proteins confirmed the interaction of OVOL2 with NCoR, HDAC1, and HDAC3, but not with SMRT (silencing mediator of retinoid and thyroid hormone receptor),^[^ ^27^ ^]^ the protein sharing ≈45% amino acid sequence identity with NCoR (Figure 3B and Figure S3A, Supporting Information). DNase I treatment did not alter these interactions, suggesting that the interactions were not mediated by DNA (Figure 3C). Given that NCoR reportedly interacts with HDAC1 and HDAC3,^[^ ^28^, ^29^, ^30^ ^]^ the observation that OVOL2 associates with NCoR, HDAC1 and HDAC3 prompted us to examine whether OVOL2 forms a complex with NcoR, HDAC1 or HDAC3. Co‐IP combined with Re‐IP revealed that OVOL2 complexes with NCoR and HDAC3, but not with HDAC1 (Figure 3D). Histidine (His) pull‐down experiments showed that purified His‐tagged OVOL2 protein interacted with in vitro translated NCoR, but not SMRT, HDAC1 or HDAC3 (Figure S3B, Supporting Information), indicating that OVOL2 does not directly interact with HDAC1 and HDAC3, and NCoR is a bridging factor for the OVOL2/NCoR/HDAC3 complex in mammalian cells. Purified OVOL2 (101–240) containing four C2H2 zinc finger domains, but not OVOL2 (1–100) containing the SNAG (snail/gfi‐1) domain and OVOL2 (241–275) containing the C‐terminal fragment, was associated with NCoR. As expected, KD of the bridging factor NCoR reduced the OVOL2–HDAC3 interaction (Figure 3E).

As NCOR is a well‐known co‐repressor, we determined whether OVOL2 inhibits glycolytic gene transcription via NCoR. Similar to OVOL2 KO, NCoR KD increased the mRNA and protein expression of GLUT1, HK2, PFKL, ALDOA, PGK1, PGAM1, ENO1, PKM2 and LDHA, but not GPI, in MDA‐MB‐231 and MCF7 cells (Figure 3F,G and Figure S3C,D, Supporting Information). Re‐expression of NCoR in NCoR KD cells rescued these effects. Importantly, NCoR KD greatly attenuated or abolished the ability of OVOL2 to inhibit glycolytic gene expression, except for GPI. Moreover, the deletion mutant OVOL2 (△101–240), which failed to interact with NCOR, did not alter NCoR‐regulated glycolytic gene expression (Figure S3E,F, Supporting Information). These data suggested that OVOL2 inhibits glycolytic gene expression, predominantly mediated via interaction with NCoR.

Next, we investigated how OVOL2 represses glycolytic gene transcription via NCoR. Given that OVOL2 forms a complex with NCoR and HDAC3, we examined whether NCoR and HDAC3 can be recruited to glycolytic gene promoters. Similar to OVOL2, NCoR and HDAC3, but not HDAC1, which also interacts with OVOL2, were recruited to the OVOL2 binding sites of GLUT1, HK2, PFKL, ALDOA, PGK1, PGAM1, ENO1, PKM2 and LDHA promoters (Figure 3H and Figure S3G, Supporting Information). NCoR and HDAC3 were not recruited to the OVOL2 binding site of the GPI promoter. Re‐ChIP experiments revealed that OVOL2 was associated with NCoR and HDAC3 on the corresponding binding sites (Figure 3I and Figure S3H, Supporting Information). OVOL2 KO abolished recruitment of NCoR and HDAC3 and increased recruitment of histone H3 lysine 27 acetylation (H3K27ac), an acetylated histone marker known to be regulated by NCoR‐HDAC3,^[^ ^31^, ^32^ ^]^ but not H3K4ac, a marker positively correlated with active transcription,^[^ ^33^, ^34^ ^]^ to OVOL2 binding sites of representative glycolytic gene promoters (HK2, PFKL, PKM2, and LDHA promoters) (Figure 3J and Figure S3I, Supporting Information). NCoR KD abrogated the recruitment of HDAC3 and promoted the recruitment of H3K27ac to OVOL2 binding sites without impacting OVOL2 recruitment. Consistent with a previous report,^[^ ^31^ ^]^ NCoR KD reduced HDAC3 expression. Notably, HDAC3 KD increased OVOL2 expression (Figure 3J and Figure S3I, Supporting Information). HDAC3 KD promoted the recruitment of OVOL2 and NcoR, simultaneously reducing the recruitment of H3K27ac to OVOL2 binding sites. This may be an additional mechanism through which HDAC3 KD inhibits glycolysis.^[^ ^35^ ^]^ Collectively, these findings suggested that OVOL2 represses glycolytic gene expression by recruiting NCoR and HDAC3.

### 2.4 OVOL2 Represses Aerobic Glycolysis through the NCoR Co‐Repressor

**Figure 4:**
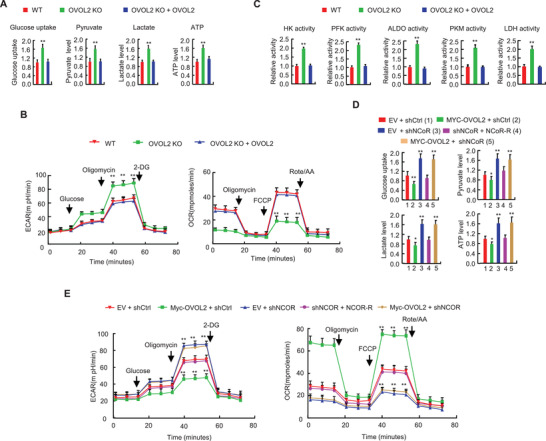
OVOL2 inhibits aerobic glycolysis through the NCoR co‐repressor. A) Glucose uptake, pyruvate level, lactate production, and ATP level in OVOL2 WT or KO MDA‐MB231 cells or OVOL2 KO MDA‐MB‐231 cells stably transfected with OVOL2. B) ECAR and OCR in the cells from (A). C) HK, PFK, ALDO, PKM, and LDH activities in the cells from (A). D) MDA‐MB‐231 cells stably expressing shNCoR or shCtrl were stably transfected with MYC‐OVOL2 or shRNA‐resistant NCoR as indicated, and glucose uptake, pyruvate level, lactate production and ATP level were then examined. E) ECAR and OCR were examined in the cells from (D). Data shown are mean ± SD of quintuplicate measurements and have been repeated 3 times with similar results. Statistical significance was assessed by one‐way ANOVA. ^**^ *p* < 0.01 versus OVOL2 WT MDA‐MB‐231 cells (A–C). ^*^ *p* < 0.05, ^**^ *p* < 0.01 versus MDA‐MB‐231 cells stably expressing EV and shCtrl (D,E).

Cancer cells metabolize glucose via aerobic glycolysis, producing lactate from pyruvate and ATP from glucose. As OVOL2 inhibits the expression of several glycolytic genes, we determined whether OVOL2 regulates the glycolytic phenotype in cultured cells. OVOL2 KO enhanced glucose uptake, pyruvate levels, lactate production and ATP levels in MDA‐MB‐231 and MCF7 cells (**Figure** **4**A and Figure S4A, Supporting Information); these effects were rescued by OVOL2 re‐expression in the OVOL2 KO cells. OVOL2 KO cells exhibited an increased extracellular acidification rate (ECAR), which reflects overall glycolytic flux, and a decreased oxygen consumption rate (OCR), an indicator of mitochondrial oxidative respiration (Figure 4B and Figure S4B, Supporting Information). Furthermore, OVOL2 re‐expression in OVOL2 KO cells rescued these effects. OVOL2 KO promoted HK, PFK, ALDO, PKM, and LDH activities in MDA‐MB‐231 and MCF7 cells (Figure 4C and Figure S4C, Supporting Information). OVOL2 re‐expression in OVOL2 KO cells rescued these effects.

Given that OVOL2 represses glycolytic gene expression through NCoR, we assessed whether OVOL2‐mediated regulation of the glycolytic phenotype depends on NCoR. Consistent with NCoR modulation of glycolytic gene expression, NCoR KD increased glucose uptake, pyruvate level, lactate production, ATP level, and ECAR and decreased OCR (Figure 4D,E and Figure S4D,E, Supporting Information). NCoR re‐expression in NCoR KD cells rescued these effects. Importantly, NCoR KD abrogated the ability of OVOL2 to regulate these effects, suggesting that OVOL2 modulates aerobic glycolysis via NCoR.

### 2.5 Aerobic Glycolysis is Critical for OVOL2 Modulation of Cancer Cell Proliferation, Invasion, and Metastasis

**Figure 5:**
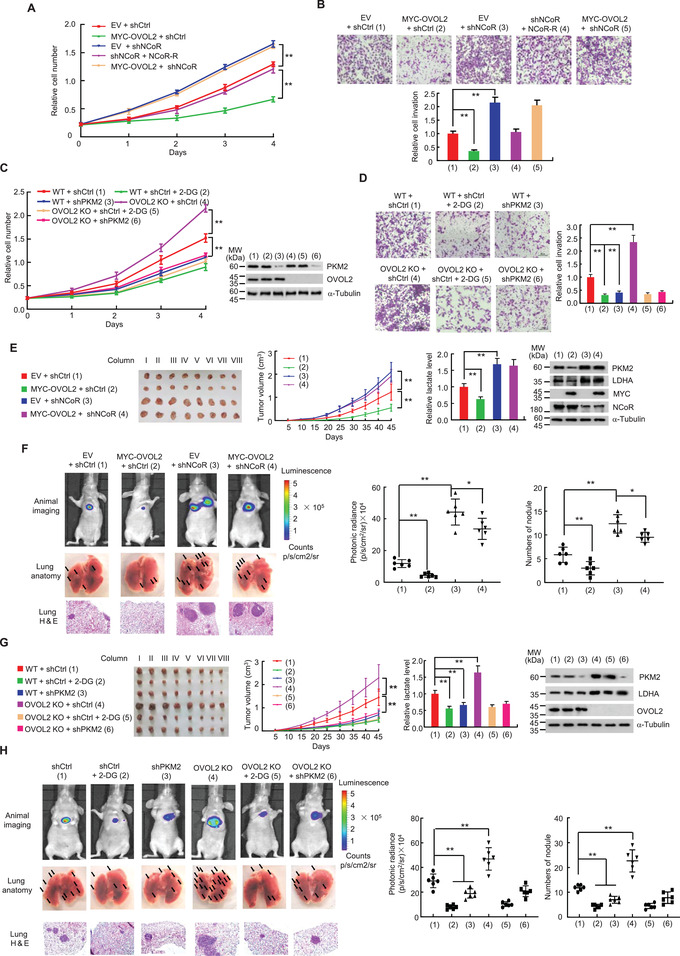
Aerobic glycolysis is responsible for OVOL2 modulation of cancer cell proliferation, invasion, and metastasis. A) MDA‐MB‐231 cells stably expressing shNCoR or shCtrl were stably transfected with MYC‐OVOL2 or NCoR‐R as indicated, and the cell proliferation curve was then determined. B) Cell invasion assay of MDA‐MB‐231 cells as described in (A). The relative cell invasions are shown in the lower panel. C) OVOL2 WT or KO MDA‐MB‐231 cells were stably transfected with PKM2 shRNA (shPKM2) or shCtrl as indicated. The cells were treated with or without 2.5 mm 2‐DG, and the cell proliferation curve was then assessed. Representative immunoblot reveals expression of PKM2 and OVOL2. D) Cell invasion assay of MDA‐MB‐231 cells as described in (C). The relative cell invasions are shown in the right panel. E) Tumor growth curve of MDA‐MB‐231 cells stably expressing MYC‐OVOL2, shNCoR, or MYC‐OVOL2 plus shNCoR as indicated. Images of xenograft tumors are shown in the right panel. The column indicates the designated number of each mouse, with each column corresponding to different mice in different groups. The lactate level of representative tumor tissues (the fourth column) was determined. Representative immunoblot shows expression of PKM2 and LDHA in representative tumor tissues (the fourth column). F) Representative bioluminescence image, lung tissues and H&E‐stained sections of the lung tissues at 30 days from nude mice injected by tail vein with MDA‐MB‐231 cells expressing firefly luciferase and the indicated constructs as described in (E) (*n* = 6). The luminescence signal is represented by an overlaid false‐color image with the signal intensity indicated by the scale (middle panel) and the number of tumor nodules shown (right panel). G) Tumor growth curve of OVOL2 WT or KO MDA‐MB‐231 cells stably expressing shPKM2 or shCtrl and treated with or without 2‐DG as indicated. The lactate level of representative tumor tissues (the fourth column) was examined. Representative immunoblot indicates expression of PKM2 and LDHA in representative tumor tissues (the fourth column). H) Representative bioluminescence image, lung tissues and H&E‐stained sections of the lung tissues at 30 days from nude mice injected by tail vein with MDA‐MB‐231 cells expressing firefly luciferase and the indicated constructs as described in (G) (*n* = 6). The luminescence signals and the number of tumor nodules are shown (middle and right panels). Statistical significance was assessed by one‐way ANOVA. **p <0.01.

As OVOL2 regulates aerobic glycolysis in an NCoR‐dependent manner and glycolysis plays an important role in modulating cancer cell proliferation, invasion, and metastasis, we first examined whether OVOL2 modulates cancer cell proliferation through NCoR in cells cultured on regular medium. OVOL2 overexpression reduced the proliferation of MDA‐MB‐231 and MCF7 cells, whereas NCoR KD increased their proliferation (**Figure** **5**A and Figure S5A, Supporting Information). Importantly, NCoR KD almost completely abolished the ability of OVOL2 to inhibit cancer cell proliferation, suggesting that OVOL2 primarily regulates cancer cell proliferation through NCoR.

Conversely, we examined the effect of OVOL2 KO on cancer cell proliferation and ATP levels using a galactose or glucose‐containing medium. As expected, cancer cells grown in a galactose‐treated medium exhibited similar proliferation behavior to those cultured in a high glucose‐treated medium (Figure S5B, Supporting Information). However, OVOL2 KO cells cultured in glucose grew faster than those cultured in galactose. The glycolytic inhibitor 2‐deoxy‐D‐glucose (2‐DG), but not the OXPHOS inhibitor oligomycin, almost completely abrogated this effect. Consistently, OVOL2 KO cells cultured in glucose produced more ATP than those cultured in galactose (Figure S5B, Supporting Information). Furthermore, 2‐DG, but not oligomycin, almost completely abolished this effect. These data suggested that increased glycolysis in OVOL2 KO cells drives enhanced ATP production, facilitating proliferation.

Similar to the results of OVOL2 modulation of cancer cell proliferation via NCoR, OVOL2 regulated the invasion of MDA‐MB‐231 and MCF7 cells in an NCoR‐dependent manner (Figure 5B and Figure S5C, Supporting Information). Interestingly, OVOL2 KO‐mediated promotion of proliferation and invasion was almost abolished by the glycolytic inhibitor 2‐deoxy‐D‐glucose (2‐DG) and KD of PKM2, a rate‐limiting glycolytic enzyme (Figure 5C,D and Figure S5D,E, Supporting Information), suggesting that glycolysis is responsible for OVOL2‐mediated modulation of cancer cell proliferation and invasion.

Consistent with cell proliferation and invasion results in vitro, OVOL2 overexpression inhibited breast tumor growth and metastasis in nude mice, whereas NCoR KD promoted breast tumor growth and metastasis (Figure 5E,F and Figure S5F, Supporting Information). NCoR KD almost abrogated the ability of OVOL2 to suppress tumor growth and metastasis, indicating that OVOL2 regulates tumor growth and metastasis predominantly through NCoR. Tumors with OVOL2 overexpression showed decreased glycolytic gene expression and lactate production, whereas those with NCoR KD showed increased glycolytic gene expression and lactate production (Figure 5E and Figure S5F, Supporting Information). NCoR KD abolished the ability of OVOL2 to regulate glycolytic gene expression and lactate production. Moreover, 2‐DG or PKM2 KD almost completely abolished the OVOL2 KO‐mediated enhancement of metastasis (Figure 5G,H and Figure S5G, Supporting Information). Overall, these data suggested that OVOL2 modulates tumor growth and metastasis mainly through NCoR‐dependent glycolysis.

### 2.6 OVOL2 Represses Glycolysis under Hypoxic Conditions

Given that hypoxia is a major feature of solid tumors and regulates OVOL2 expression, we examined whether OVOL2 modulates glycolysis under hypoxic conditions. OVOL2 KO enhanced the transcription of glycolytic genes in MDA‐MB‐231 and MCF7 cells under both normoxic and hypoxic conditions (Figure S6A, Supporting Information). Consistent with this finding, OVOL2 was recruited to glycolytic gene promoters under hypoxic conditions (Figure S6B, Supporting Information). As expected, OVOL2 KO increased glucose uptake, pyruvate levels, lactate production, and ATP levels under both normoxic and hypoxic conditions (Figure S6C, Supporting Information). Moreover, hypoxia increased cancer cell proliferation, and OVOL2 overexpression almost completely abrogated hypoxia‐stimulated cancer cell proliferation (Figure S6D, Supporting Information), suggesting a key role for OVOL2 in inhibiting hypoxia‐induced cancer cell proliferation. Overall, these data suggested that OVOL2 regulates glycolysis and cancer cell proliferation under hypoxic conditions.

### 2.7 OVOL2 is Conversely Regulated by the MDM2 Oncoprotein and p53 Tumor Suppressor Protein

**Figure 6:**
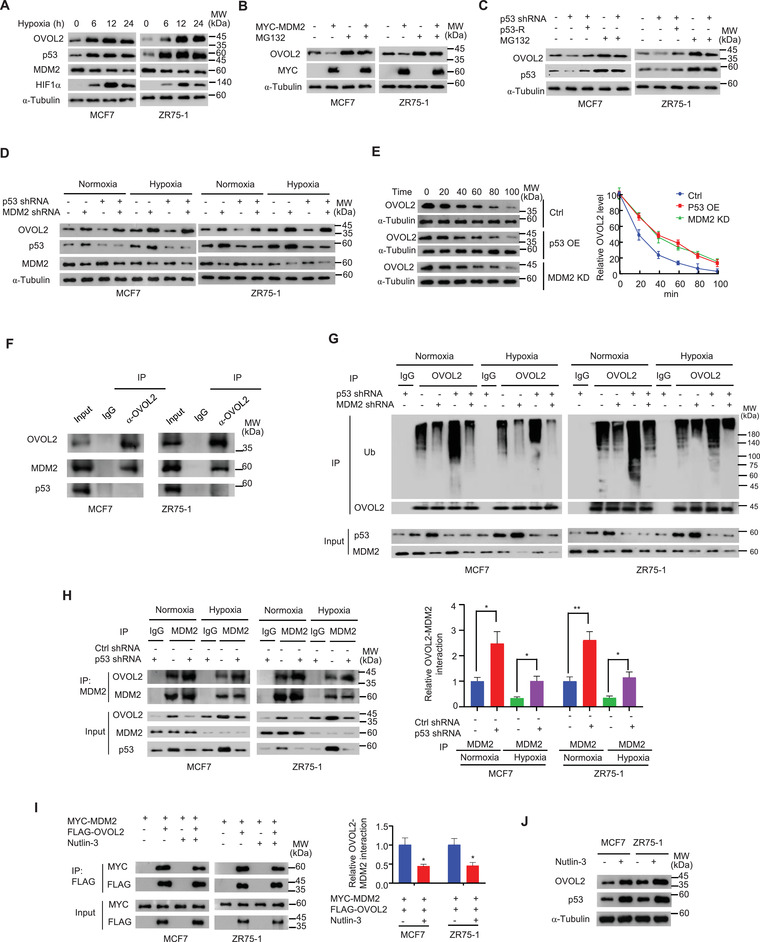
OVOL2 is conversely modulated by the MDM2 oncoprotein and p53 tumor suppressor protein. A) Immunoblot analysis of OVOL2, p53, MDM2, and HIF1*α* expression in MCF7 and ZR75‐1 cells under hypoxic conditions (1% O_2_) for indicated times. B) Immunoblot analysis of OVOL2 expression in MCF7 or ZR75‐1 cells transfected with MYC‐MDM2 and treated with 10 µm MG132 as indicated. C) Immunoblot analysis of OVOL2 expression in MCF7 and ZR75‐1 cells stably transfected with p53 shRNA or control shRNA or p53 shRNA plus shRNA‐resistant p53 (p53‐R) and treated with 10 µm MG132 as indicated. D) Immunoblot analysis of OVOL2 expression in MCF7 and ZR75‐1 cells stably transfected with control shRNA, p53 shRNA, MDM2 shRNA, or p53 shRNA plus MDM2 shRNA under hypoxic conditions (1% O_2_) for 12 h. E) Immunoblot analysis of OVOL2 expression in MCF7 cells stably transfected with p53 (p53‐OE) or MDM2 shRNA (MDM2 KD) at indicated times after exposure to the protein synthesis inhibitor cycloheximide (20 mg mL^−1^). Graphs represent the quantification of immunoblot data. F) MCF7 or ZR75‐1 cells were immunoprecipitated with anti‐OVOL2 or normal IgG, and precipitates were analyzed by IB with indicated antibodies. G) Cell lysates from MCF‐7 or ZR75‐1 cells stably transfected with p53 shRNA, MDM2 shRNA or p53 shRNA plus MDM2 shRNA and treated with 10 µm MG132 under normoxic or hypoxic conditions (1% O_2_) were immunoprecipitated with anti‐OVOL2 or normal IgG, followed by IB with the indicated antibodies. Ub, ubiquitin. H) Cell lysates from MCF‐7 or ZR75‐1 cells stably transfected with p53 shRNA or control shRNA (Ctrl shRNA) under normoxic or hypoxic conditions (1% O_2_) were immunoprecipitated with anti‐MDM2 or normal IgG, followed by IB with indicated antibodies. Representative results are shown (*n* = 3). Graphs represent the quantification of immunoblot data (the ratio of a OVOL2 band to the corresponding MDM2 band in IP). I) MCF7 or ZR75‐1 cells transiently transfected with MYC‐MDM2 or MYC‐MDM2 plus FLAG‐OVOL2 and treated with or without 10 µm nutlin‐3 were immunoprecipitated with anti‐FLAG, followed by IB with indicated antibodies. Representative results are shown (*n* = 3). Graphs represent the quantification of immunoblot data (the ratio of a MYC band to corresponding FLAG band in IP). J) Immunoblot analysis of OVOL2 expression in MCF7 and ZR75‐1 cells treated with 10 µm nutlin‐3. Data shown are mean ± SD of three independent experiments (H,I). Data were analyzed using two‐tailed Student's *t*‐test and one‐way ANOVA. *p < 0.05, **p < 0.01.

As OVOL2 is a critical glycolysis inhibitor, we examined the mechanism underlying OVOL2 expression. Based on our observation that hypoxia inhibited OVOL2 mRNA expression, we examined how hypoxia regulates OVOL2 expression. Unexpectedly, similar to the well‐known hypoxia‐inducible protein, hypoxia‐inducible factor 1*α* (HIF1*α*), hypoxia could induce the expression of OVOL2 protein in MCF7, ZR75‐1 and MDA‐MB‐231 breast cancer cells (**Figure** **6**A and Figure S7A, Supporting Information), suggesting that OVOL2 expression is regulated at the protein level. Unlike MCF7 and ZR75‐1 cells, MDA‐MB‐231 cells harbor a p53 missense mutation (expressing p53 R280K), indicating that p53 is not essential for OVOL2 induction. As expected, OVOL2 mRNA levels were not consistent with their observed protein levels (Figure S7B,C, Supporting Information). Thus, we investigated whether the ubiquitin‐proteasome pathway was involved in regulating OVOL2 protein expression. The ubiquitin ligase enzyme E3 performs the last step in the ubiquitination cascade and targets specific substrates for proteasome‐mediated degradation. To identify E3 ubiquitin ligases potentially responsible for OVOL2 degradation, we revisited our Co‐IP/mass spectrometry (MS) analysis results. We found MDM2, an E3 ubiquitin ligase known to degrade p53. As previously reported,^[^ ^36^, ^37^, ^38^, ^39^ ^]^ MDM2 was repressed, and p53 was induced by hypoxia (Figure 6A). It should be noted that hypoxia‐mediated induction of p53 did not occur in a mutant hepatoma cell line that failed to stimulate HIF1*α* or embryonic stem cells derived from mice lacking HIF1*α*,^[^ ^40^ ^]^ suggesting that the hypoxic induction of p53 is HIF1*α*‐and cell type‐dependent. In breast cancer cells, MDM2 overexpression or p53 KD reduced OVOL2 protein expression, and the proteasome inhibitor MG132 blocked the MDM2 overexpression‐ or p53 KD‐mediated reduction in OVOL2 expression (Figure 6B,C). The effect of p53 KD on OVOL2 expression was rescued by p53 re‐expression in p53 KD cells. In contrast, MDM2 KD increased OVOL2 expression, which was rescued by MDM2 re‐expression in MDM2 KD cells (Figure S7D, Supporting Information). p53 or MDM2 KD did not alter OVOL2 mRNA expression (Figure S7E, Supporting Information). Although hypoxia could induce OVOL2 protein in p53 R280K‐expressing MDA‐MB‐231 cells, WT p53, but not p53 R280K, activated OVOL2 expression (Figure S7F, Supporting Information), suggesting that OVOL2 protein can be induced by hypoxia in a p53‐dependent and ‐independent manner. Under both normoxic and hypoxic conditions, MDM2 KD abolished the ability of p53 KD to decrease OVOL2 expression, whereas p53 KD did not affect the ability of MDM2 KD to increase OVOL2 expression (Figure 6D), suggesting that p53 regulates OVOL2 expression via MDM2 under normoxic and hypoxic conditions. Moreover, p53 overexpression or MDM2 KD increased the OVOL2 protein half‐life (the half‐life of OVOL2 was ≈20 min) (Figure 6E).

Next, we examined how MDM2 and p53 differently regulate OVOL2 expression. Accordingly, we first determined whether OVOL2 interacted with MDM2 and p53. As previously reported, MDM2 interacts with p53 (Figure S7G, Supporting Information). Importantly, OVOL2 was associated with MDM2, but not with p53, in mammalian cells (Figure 6F and Figure S7G,H, Supporting Information) and in vitro (Figure S7i, Supporting Information). As MDM2 is an E3 ubiquitin ligase, we investigated whether MDM2 ubiquitinated OVOL2. Under both normoxic and hypoxic conditions, MDM2 KD reduced OVOL2 ubiquitination, whereas p53 KD increased OVOL2 ubiquitination (Figure 6G). MDM2 KD abolished the ability of p53 KD to increase OVOL2 ubiquitination, whereas p53 KD did not affect the ability of MDM2 KD to decrease OVOL2 ubiquitination; these findings suggested that p53 regulates OVOL2 ubiquitination via MDM2 and MDM2 is an E3 ubiquitin ligase for OVOL2. Thus, we hypothesized that p53 regulates OVOL2 expression by competing with OVOL2 to bind to MDM2. Indeed, p53 overexpression dose‐dependently inhibited the interaction between OVOL2 and MDM2 (Figure S7J, Supporting Information). In contrast, p53 KD enhanced the OVOL2–MDM2 interaction under normoxic and hypoxic conditions (Figure 6H). Nutlin‐3 is a small‐molecule inhibitor that inhibits MDM2 binding to p53 and prevents p53 degradation. Interestingly, nutlin‐3 also reduced MDM2 binding to OVOL2 (Figure 6I). As expected, nutlin‐3 increased OVOL2 expression (Figure 6J). Overall, these data indicated that p53 regulates OVOL2 expression via MDM2.

We next examined whether the interaction between p53 and MDM2 can impact p53‐mediated modulation of OVOL2 expression. Accordingly, we used a p53 (L14Q,F19S) mutant known that defectively binds to MDM2.^[^ ^41^, ^42^ ^]^ p53 (L14Q,F19S) failed to decrease the OVOL2–MDM2 interaction and to increase OVOL2 expression (Figure S7K,L, Supporting Information). To exclude the possibility that p53 downstream target proteins play a role in disrupting the OVOL2–MDM2 interaction, we used a p53 (S15A) mutant, which markedly reduced p53 transcriptional activity but had no effect on the p53–MDM2 interaction.^[^ ^43^, ^44^ ^]^ Similar to WT p53, p53 (S15A) reduced the OVOL2–MDM2 interaction and enhanced OVOL2 expression (Figure S7K,L, Supporting Information).

### 2.8 Clinical Relevance of OVOL2 Expression and Glycolysis in Breast Cancer

**Figure 7:**
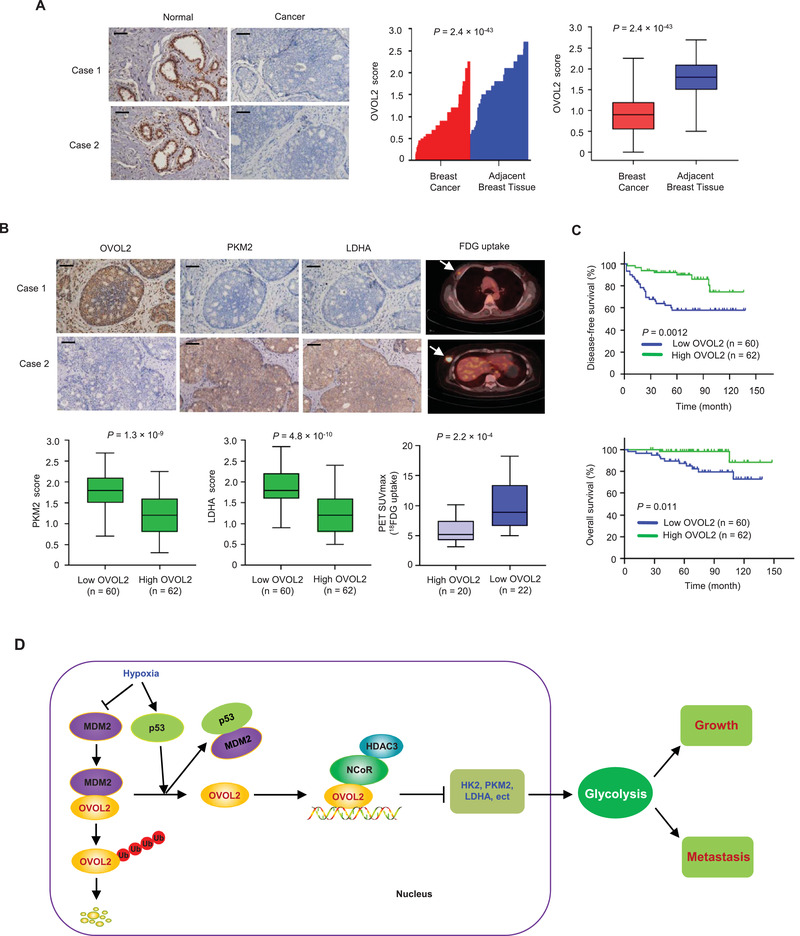
Clinical relevance of OVOL2 expression and glycolysis in patients with breast cancer. A) OVOL2 expression in 122 cancerous breast tissues and matched adjacent normal breast tissues was examined by immunohistochemistry (IHC). Relative OVOL2 expression levels were plotted and compared between normal and cancer tissues (Paired‐sample *t*‐test). Scale bar, 100 µm. B) OVOL2, PKM2, and LDHA expression in 122 patients with breast cancer from (A) were assessed by IHC, and ^18^FDG uptake in 42 patients with breast cancer by FDG PET scans. The correlation between OVOL2 and PKM2 and LDHA was determined by independent‐sample *t*‐test, while the correlation between OVOL2 and ^18^FDG uptake was determined using the Mann–Whitney U test. Case 1 and case 2 refer to two representative samples categorized by low and high OVOL2 expression. Arrows show tumor glucose uptake. Scale bar, 100 µm. C) The disease‐free and overall survival curves related to low and high expression of OVOL2 were analyzed in 122 patients with breast cancer from (A) using the Kaplan–Meier method. D) A proposed model underlying the role of OVOL2 in cancer glycolysis and tumor growth and metastasis. OVOL2 inhibits the transcription of glycolytic genes (e.g., HK2) by recruiting NCoR and HDAC3. OVOL2 can be repressed by MDM2‐mediated ubiquitination and degradation and induced under hypoxia by p53, which binds MDM2 and blocks the MDM2–OVOL2 interaction. Thus, the p53/MDM2/OVOL2 axis links glycolytic gene expression to glycolysis and tumor growth and metastasis.

It has been reported that OVOL2 expression is negatively correlated with human breast cancer progression, and patients with breast cancer exhibiting high OVOL2 expression have longer relapse‐free survival (RFS) than patients with low OVOL2 expression.^[^ ^15^ ^]^ However, a comparison of OVOL2 expression between breast cancer and normal breast tissues has not been performed. We found that OVOL2 protein expression was significantly downregulated in breast cancer tissues when compared with normal tissues (**Figure** **7**A). Patients with breast cancer exhibiting low OVOL2 expression had shorter disease‐free survival (DFS) and overall survival (OS) than those with high OVOL2 expression (Figure 7B). Importantly, OVOL2 expression was inversely correlated with PKM2 and LDHA expression (Figure 7C). Moreover, patients with breast tumors exhibiting increased glucose uptake, as assessed by ^18^F‐fluoro‐2‐deoxy‐D‐glucose positron emission tomography (^18^FDG PET) scans, revealed reduced OVOL2 expression. We confirmed the specificity of OVOL2, PKM2, and LDHA antibodies by immunohistochemical staining of breast cancer tissues or immunoblotting with cell lysates (Figure S8A–F, Supporting Information). External datasets from TCGA (The Cancer Genome Atlas) revealed that OVOL2 mRNA expression was significantly upregulated in breast cancer tissues when compared with that observed in normal tissues (Figure S8G, Supporting Information). Given the unavailability of data for OVOL2 protein expression in normal breast tissues from external Clinical Proteomic Tumor Analysis Consortium (CPTAC) datasets, we could not compare OVOL2 expression between breast cancer tissues and normal breast tissues. The discrepancy between expression results for OVOL2 mRNA and protein levels in breast tissues may be attributed to our observation that OVOL2 mRNA levels are inconsistent with their protein levels (Figure S7B,C, Supporting Information).

## 3 Discussion

Aerobic glycolysis is a hallmark of cancer cells. A comprehensive understanding of the roles of regulatory factors in aerobic glycolysis will ultimately lead to novel therapeutic approaches for cancer treatment. Transcription factors play key roles in the regulation of aerobic glycolysis. The transcription factor p53, a well‐known tumor suppressor, can inhibit aerobic glycolysis by directly repressing the transcription of GLUT1 and GLUT4.^[^ ^10^ ^]^ Except for p53, how aerobic glycolysis is directly transcriptionally inhibited is rarely known. In the present study, we identify OVOL2 as a critical transcriptional repressor of aerobic glycolysis. OVOL2 directly inhibits the expression of several key glycolytic genes that facilitate aerobic glycolysis, tumor growth, and metastasis. Using different cancer cell lines, OVOL2 KO MEFs, OVOL2 conditional KO mice and tumor specimens, we revealed that OVOL2 modulated glycolytic gene expression, glucose uptake and lactate production. Our study demonstrated that OVOL2 is a master transcription factor controlling aerobic glycolysis, suggesting a causal role for OVOL2 in glycolysis inhibition (Figure 7D).

Most eukaryotic transcription factors recruit cofactors, including co‐activators and co‐repressors, to activate or repress target gene transcription.^[^ ^45^ ^]^ Herein, OVOL2 interacted with the NCoR co‐repressor to inhibit glycolytic gene transcription. It has been reported that OVOL2 acts as a tumor suppressor. OVOL2 expression is down‐regulated in various cancers, including hepatocellular carcinoma,^[^ ^13^ ^]^ colorectal cancer^[^ ^14^ ^]^ and nasopharyngeal carcinoma,^[^ ^16^ ^]^ and correlates with the clinical stage and/or histological grade in some cancers, including colorectal cancer,^[^ ^14^ ^]^ lung adenocarcinoma,^[^ ^46^ ^]^ osteosarcoma^[^ ^47^ ^]^ and hepatocellular carcinoma.^[^ ^13^ ^]^ Low OVOL2 levels are associated with poor OS in patients with hepatocellular carcinoma, colorectal cancer or nasopharyngeal carcinoma. OVOL2 can inhibit epithelial‐to‐mesenchymal transition and cancer cell migration, invasion, and metastasis. A previous study has shown that high OVOL2 expression predicts a prolonged RFS in patients with breast cancer and OVOL2 inhibits breast tumor metastasis.^[^ ^15^ ^]^ We demonstrated that OVOL2 expression is down‐regulated in breast cancer and that patients with high OVOL2 expression show prolonged DFS and OS. Moreover, our findings revealed that OVOL2 regulates breast cancer cell migration and metastasis, as well as modulates breast cancer cell proliferation in vitro and breast tumor growth in vivo. NCoR and SMRT are well‐investigated co‐repressors, with ≈45% amino acid sequence identity. Deregulated functions of NCoR and SMRT have been observed in various cancers, including breast and prostate cancer.^[^ ^48^, ^49^, ^50^ ^]^ For instance, the levels of NCoR/SMRT, co‐repressors of the transcription factor estrogen receptor *α* (ER*α*), are critical for the repression of ER*α* transcriptional activity and ER*α* target gene transcription mediated by tamoxifen, a well‐known drug used for breast cancer therapy. NCoR levels were down‐regulated in breast invasive ductal carcinomas. NCoR knockdown increased the invasive capacity of breast cancer cells and breast tumor growth in vivo.^[^ ^51^ ^]^ However, the role of NCoR in glucose metabolism is unknown. Our study indicates that OVOL2 interacts with NCoR, but not SMRT. Moreover, like OVOL2, NCoR represses the transcription of several glycolytic genes, and recruitment of NCoR to glycolytic gene promoters requires OVOL2. OVOL2 could inhibit glycolytic gene expression, glycolysis and breast cancer cell proliferation, invasion and metastasis in vitro and in vivo, mainly through NCoR. Unlike OVOL2, NCoR failed to inhibit GPI expression, suggesting that OVOL2 regulates GPI expression through other co‐repressors. Glycolysis is critical for OVOL2‐mediated regulation of breast cancer cell proliferation, invasion and metastasis. Given that OVOL2 is down‐regulated in cancer, correlates with clinical outcomes and represses glycolysis, OVOL2 is expected to be a promising target for cancer therapy.

NCoR represses gene transcription via the recruitment of histone‐modifying enzymes, especially HDACs.^[^ ^27^, ^28^, ^29^ ^]^ HDACs deacetylate lysine forms residues on histone tails, thereby inducing chromatin compaction and gene silencing. HDAC3 is reportedly the primary HDAC in NCoR/SMRT complexes although other HDACs, including HDAC1, 4, 5 and 7, can interact with NCoR/SMRT. NCoR and SMRT are considered to bind HDAC3 competitively. However, NCoR and SMRT do not co‐exist in the same HDAC3 complex.^[^ ^52^, ^53^ ^]^ We found that recruitment of NCoR‐HDAC3 to glycolytic gene promoters requires OVOL2 and NCoR is required for recruiting HDAC3 to glycolytic gene promoters. HADC1 is not involved in the OVOL2‐mediated regulation of glycolytic gene transcription. However, whether other HDACs are responsible for OVOL2 modulation of glycolytic gene transcription needs to be determined. In contrast to OVOL2, HDAC3 is up‐regulated in many cancers including breast cancer.^[^ ^54^ ^]^ High HDAC3 expression correlates with poor prognosis in patients with cancer. HDAC3 promotes cancer cell proliferation and metastasis. Moreover, HDAC3 enhances aerobic glycolysis in cancer cells. Our observation that HDAC3 KD increased OVOL2 expression might explain the HDAC3‐mediated enhancement of glycolysis. Another possible explanation is that HDAC3 interacts with proteins other than OVOL2. Indeed, the glycolytic enzyme PGK1 is a substrate for HDAC3. HDAC3 deacetylates PGK1, resulting in enhanced PGK1 activity and glycolysis.^[^ ^55^ ^]^

The p53–MDM2 axis regulates OVOL2 expression; however, we cannot exclude the possibility that other factors could modulate OVOL2 expression. The E3 ubiquitin ligase MDM2 ubiquitinates and degrades the OVOL2 protein. p53 reduces the MDM2–OVOL2 interaction, thereby increasing OVOL2 levels. Nutlin‐3,^[^ ^56^, ^57^ ^]^ a small‐molecule inhibitor that induces cancer cell cycle arrest and apoptosis by blocking the p53–MDM2 interaction and activating p53, inhibits the OVOL2–MDM2 interaction and increases OVOL2 levels. Several small‐molecule inhibitors that target the p53–MDM2 interaction are currently in clinical trials. The potential side effects of p53 activation in normal tissues are under investigation. It is conceivable that p53‐activating agents may also stimulate OVOL2 expression. We showed that OVOL2 could be activated in a p53‐dependent and ‐independent manner. Thus, p53‐independent activation of OVOL2 by small molecules may also be an excellent strategy for cancer treatment.

## 4 Experimental Section

### Plasmids, Small‐Interfering RNA (siRNA), Short Hairpin RNA (shRNA), Lentiviruses, and Reagents

Eukaryotic expression plasmids encoding FLAG‐ or MYC‐tagged or untagged proteins were constructed by cloning the PCR‐amplified fragments into pcDNA3 (Invitrogen, Carlsbad, CA). Prokaryotic vectors encoding His‐fusion proteins were constructed using the pET32a vector (Novagen, Darmstadt, GER). Glycolytic gene promoter luciferase reporters were generated by cloning PCR‐amplified promoter fragments from genomic DNA into the pGL4‐basic vector (Promega, Madison, WI). siRNA/shRNA‐resistant expression vectors and mutants for luciferase reporters were constructed using recombinant PCR. cDNA target sequences of siRNAs and/or shRNAs for OVOL2, NCoR, HDAC3, PKM2, LDHA, and p53 are listed in Table S2, Supporting Information. Lentiviral vectors for gene overexpression were generated by cloning PCR‐amplified gene fragments into pCDH (System Biosciences, Johnstown, PA). Lentiviral shRNA vectors were constructed by inserting the corresponding fragments into the pSIH‐H1‐Puro vector (System Biosciences, Johnstown, PA).

Anti‐GPI (sc‐33777), anti‐PFKL (sc‐292523), anti‐ENO1 (sc‐15343), anti‐MDM2 (sc‐965), and anti‐ubiquitin (sc‐8017HRP) antibodies were purchased from Santa Cruz Biotechnology (Dallas, TX). Anti‐FLAG (A8592), anti‐FLAG M2 agarose (A2220), anti‐c‐MYC gel (E6654), anti‐c‐MYC‐peroxidase (A5598), anti‐GAPDH (G9295), anti‐NCoR (17‐10260), anti‐p53 (17‐613), anti‐HDAC1 (17‐608), anti‐HDAC3 (17‐10238), anti‐H3K4ac (17‐10050), and anti‐H3K27ac (17‐683) antibodies were purchased from Sigma‐Aldrich(Saint Louis, MO). The anti‐GST (RPN1236) and anti‐His (27‐4710‐01) antibodies were obtained from GE Healthcare (Chicago, IL). Anti‐GLUT1 (21829‐1‐AP), anti‐ALDOA (11217‐1‐AP), anti‐PGAM1 (16126‐1‐AP), anti‐PGK1 (17811‐1‐AP), anti‐LDHA (19987‐1‐AP), anti‐HIF1*α* (20960‐1‐AP), anti‐*α*‐tubulin (11224‐1‐AP), and anti‐SMRT (20017‐1‐AP) antibodies were obtained from Proteintech (Chicago, IL). Anti‐PKM2 (4053S), anti‐HK2 (2867S), and anti‐MDM2 (86 934) antibodies were purchased from Cell Signaling Technology (Danvers, MA). Anti‐OVOL2 (PA5‐115700) for immunohistochemistry (IHC) or immunoblotting and anti‐EGFP (CAB4211) were purchased from Invitrogen (Carlsbad, CA). Anti‐OVOL2 (ab169469) for immunoblotting was purchased from Abcam (Cambridge, UK). Anti‐OVOL2 (TA345332) for immunoprecipitation and immunoblotting was obtained from Origene (Rockville, MD).

### Cell Lines, Transfection, and Infection

Human embryonic kidney HEK293T cells, human breast cancer MCF7, ZR75‐1 and MDA‐MB‐231 cells, and human colorectal cancer HCT116 cells were purchased from American Type Culture Collection (Rockefeller, MD) and previously tested for mycoplasma contamination. The MDA‐MB‐231 cell line with the luciferase label was a gift from Prof. Yongfeng Shang at the Capital Medical University (Beijing, China). Cells were routinely cultured in Dulbecco's Modified Eagle Medium (DMEM) containing 25 mm glucose (Invitrogen, Carlsbad, CA) and 10% fetal bovine serum (FBS; Hyclone) at 37 °C. Lipofectamine 3000 reagent (Invitrogen, Carlsbad, CA) and Lipofectamine RNAiMAX (Invitrogen, Carlsbad, CA) were used to transfect plasmids and siRNAs, respectively. For plasmid transfection only, cells were collected 24 h after transfection for further analysis. For siRNA transfection, cells were harvested 48 h post‐transfection for further study. For the transfection of siRNAs and plasmids, cells were first transfected with siRNAs. Twenty‐four hours later, the cells were transfected with the plasmids and collected 24 h after transfection. Lentiviruses were produced by co‐transfecting HEK293T cells with recombinant lentiviral vectors and pPACK Packaging Plasmid Mix (System Biosciences, Johnstown, PA) using Megatran reagent (Origene, Rockville, MD), according to the manufacturer's protocols. Viral supernatants were harvested 48 h after transfection, and titers were detected. The target cells were then infected with lentiviral constructs containing 8 µg mL^−1^ polybrene (Sigma‐Aldrich, Saint Louis, MO). To establish stable cell lines, infected MDA‐MB‐231 and MCF7 cells were selected in 1 µg mL^−1^ puromycin, and transfected MDA‐MB‐231 and MCF7 cells were selected in 600 µg mL^−1^ G418 and 800 µg mL^−1^ G418, respectively.

### OVOL2 Knockout Cancer Cell Lines

*OVOL2* KO cancer cells were generated using the CRISPR/Cas9 system. The CRISPRs were designed using the CRISPR design web tool (http://crispr.mit.edu). The single guide RNA (sgRNA) sequence targeted by *OVOL2* is TTCGCTCTCGGGGGCGTG. Single guide RNA (sgRNA) was cloned into the lentiCRISPRv2 vector (Addgene #52 961). Recombinant lentiviruses were produced by co‐transfecting HEK293T cells with a lentiviral vector using the Megatran reagent (Origene, Rockville, MD). MDA‐MB‐231 and MCF7 cells were infected with purified lentiviruses combined with 8 µg mL^−1^ polybrene (Sigma‐Aldrich, Saint Louis, MO) and then selected with 1 µg mL^−1^ puromycin. Stable KO cell lines were confirmed by PCR amplification of genomic sequences, DNA sequencing, and immunoblotting. CRISPR cell lines were clonal. Rescue experiments were performed to avoid off‐target effects.

### OVOL2 Conditional Knockout Mice

All animal experiments were performed in compliance with the Guide for the Care and Use of Laboratory Animals and were approved by the Institutional Animal Care Committee of the Beijing Institute of Biotechnology (IACUC‐DWZX‐2020‐768). *OVOL2‐*floxed KO mice, which possess a loxP site flanking exon 3 in the *OVOL2* gene, were generated using the TurboKnockout approach (Cyagen Biosciences, Inc., Sunnyvale, CA). Positive female founder and WT male mice were bred to obtain F1 *OVOL2* heterozygous mice (*OVOL2* ^fl/+^). Male and female *OVOL2* ^fl/+^ mice were crossed to generate *OVOL2* homozygote mice (*OVOL2* ^fl/fl^). *OVOL2* ^fl/fl^ mice were bred with MMTV‐Cre mice that express Cre recombinase under the control of the mouse mammary tumor virus (MMTV) promoter to obtain MMTV‐Cre; *OVOL2* ^fl/+^ mice. Finally, the MMTV‐Cre; *OVOL2* ^fl/+^ mice were crossed to obtain MMTV‐Cre; *OVOL2* ^fl/fl^ mice. MEFs were isolated as described previously.^[^ ^58^ ^]^ Briefly, tissues from fetal mice were incubated in trypsin solution to obtain a single‐cell suspension. The suspension was then washed twice in MEF medium and incubated at 37 °C. Cre‐expressing adenovirus AD‐Cre‐EGFP (BioWit Technologies, Shenzhen, China) was added to *OVOL2* ^fl/fl^ MEFs to knock out OVOL2.

### Transcriptome Sequencing (RNA‐Seq)

A minimum of 3 µg of total RNA was oligo (dT)‐selected using the Dynabeads mRNA purification kit (Invitrogen, Carlsbad, CA). mRNA extracted from the total RNA was fragmented into short fragments with a fragmentation buffer (Ambion, Austin, TX), and these short fragments were used as templates to synthesize double‐stranded cDNA. The cDNA library was constructed and sequenced on an Illumina HiSeq 2000 sequencing platform (Berry Genomics, Beijing, China). The gene expression levels for each transcript were assessed as the number of reads per kilobase of exon model per million mapped reads (RPKM) using only uniquely mapped reads in the exonic regions. A gene was considered significantly and differentially expressed if its expression differed between any two samples with a fold change >2 and a *p*‐value < 0.05, as calculated by Cufflinks. RNA‐Seq data are available in the Gene Expression Omnibus database (www.ncbi.nlm.nih.gov/geo/, accession numbers GSE166203 and GSE189947).

### Reverse Transcription‐Quantitative PCR (RT‐qPCR)

Total RNA was extracted using TRIzol reagent, according to the manufacturer's instructions (Invitrogen, Carlsbad, CA). Cells were homogenized with TRIzol reagent, vortexed for 1 min with 200 µL chloroform, and centrifuged at 1.3 × 10^4^ rpm for 10 min at 4 °C, thus generating two phases. The upper aqueous phase (containing RNA) was precipitated with isopropanol at −20 °C for 1 h and centrifuged at 1.2 × 10^4^ rpm for 15 min. RNA pellets were washed with 70% (v/v) ethanol and 100% (v/v) ethanol in succession, air‐dried, and dissolved in 100–200 µL of nuclease‐free water. Then, 2 µg of total RNA was reverse transcribed into first‐strand cDNA with oligo (dT) primers using moloney murine leukemia virus reverse transcriptase (Promega, Madison, WI). Subsequently, 1 µL of the first‐strand cDNA synthesis reaction mixture was used for PCR amplification in a total volume of 50 µL. qPCR was performed in triplicate in a 20 µL reaction mixture containing 10 µL of SYBR Premix Ex Taq Master Mix (2×) (Takara, Osaka, Japan), 0.5 µm of each primer, and 10 ng cDNA. The relative expression was calculated using the comparative Ct method. The primers used for real‐time PCR analysis are listed in Table S3, Supporting Information.

### Luciferase Reporter Assay

Luciferase reporter assays were performed according to the manufacturer's instructions (Promega, Madison, WI). Briefly, cells were transfected with 1 µg of promoter luciferase reporter, 0.5 µg of OVOL2 expression plasmid or empty vector, and 0.1 µg of a *β*‐galactosidase reporter. Twenty‐four hours after transfection, the cells were harvested, lysed, and centrifuged to obtain supernatants. Then, 10 µL of the supernatant was mixed with 10 µL of the Luciferase Assay Reagent per tube, and luciferase activity was determined using a luminometer. For the *β*‐galactosidase activity assay, supernatants were incubated in assay buffer, and the optical density (OD) values were assessed at 420 nm using a microplate reader.

### Chromatin Immunoprecipitation (ChIP) and Re‐ChIP

ChIP experiments were performed using the Magna ChIP G Assay Kit (Millipore, Boston, MA), according to the manufacturer's protocol. Briefly, cells were cross‐linked, pelleted, and resuspended in lysis buffer. The cells were sonicated and centrifuged to collect the supernatants. After incubating supernatants with indicated antibodies and Protein G magnetic beads, the beads were washed, and the precipitated chromatin complexes were harvested, purified, and de‐crosslinked at 62 °C for 2 h, followed by incubation at 95 °C for 10 min. The precipitated DNA fragments were examined using real‐time PCR. For re‐ChIP experiments, complexes were eluted from the primary immunoprecipitation by incubation with 10 mm DTT at 37 °C for 30 min and diluted 1:50 in re‐ChIP buffer (150 mm NaCl, 1% Triton X‐100, 2 mm EDTA, 20 mm Tris‐HCl; pH 8.1). The diluted complexes were immunoprecipitated with secondary antibodies and analyzed by real‐time PCR. The primers used for ChIP and re‐ChIP are listed in Table S4, Supporting Information.

### Mass Spectrometry (MS)

The FLAG‐tagged OVOL2 complex was obtained by immunoprecipitation with anti‐FLAG from 10^8^ MCF7 cells stably expressing FLAG‐OVOL2, according to the manufacturer's protocol (Sigma‐Aldrich, Saint Louis, MO). Cells were lysed in IP buffer (20 mm Tris at pH 8.0, 0.25 m NaCl, 0.5% NP‐40, 5 mm EDTA) and centrifuged to obtain supernatants. The supernatants were immunoprecipitated using anti‐FLAG agarose beads for 4 h at 4 °C. The beads were washed four times with IP buffer, and the FLAG‐tagged OVOL2 complex was eluted with the FLAG peptide. Gradient sodium dodecyl sulfate (SDS)‐polyacrylamide gel electrophoresis (PAGE) was performed to separate protein complexes, followed by silver staining and MS sequencing. In‐solution and in‐gel digestions were conducted according to a previously published approach.^[^ ^59^ ^]^ Briefly, the gel bands were minced and destained with 50% acetonitrile in 50 mm ammonium bicarbonate. Proteins were reduced with 10 mm DTT at 56 °C and alkylated with 55 mm iodoacetamide at room temperature. Trypsin digestion was performed overnight at 37 °C with gentle shaking. Digested peptides were isolated using 1% trifluoroacetic acid in 50% acetonitrile, vacuum‐dried, and reconstituted in 0.1% formic acid. The treated samples were analyzed using nanoLC‐MS/MS (nanoACQUITY UPLC and SYNAPT G2 HD mass spectrometer, Waters Milford, MA). MS/MS data were generated with a data‐dependent analysis mode and analyzed using PLGS 2.4 software (Waters, Milford, MA), and the resulting peak list was searched against the NCBI database with the MASCOT search engine.

### Co‐Immunoprecipitation (Co‐IP) and His Pull‐Down

For Co‐IP, cells were lysed in 500 µL of lysis buffer (50 mm Tris at pH 8.0, 500 mm NaCl, 0.5% Nonidet P‐40, 1 mm dithiothreitol, and protease inhibitor tablets; Roche Applied Science, Basel, Switzerland), and immunoprecipitated with anti‐FLAG or Myc‐agarose beads for 4 h at 4 °C or indicated antibodies overnight at 4 °C. After washing thrice with lysis buffer, immunoprecipitates were eluted in SDS sample buffer and resolved by SDS‐PAGE. The immunocomplexes were analyzed by immunoblotting with indicated antibodies. For His pull‐down experiments, His‐fusion proteins were expressed and purified according to the manufacturer's protocol (QIAGEN, Dusseldorf, Germany). His‐fusion proteins were induced in *Escherichia coli* (BL21) with 0.5 mm IPTG at 20 °C for 20 h. *E. coli* were harvested, resuspended in lysis buffer, sonicated, and centrifuged. Supernatants were incubated with nickel beads (QIAGEN, Dusseldorf, Germany) for 4 h at 4 °C.

The beads were washed three times with lysis buffer. The bound His‐ or His‐fusion proteins were eluted with imidazole and dialyzed against a lysis buffer. The expression vectors for NCoR, SMRT, HDAC1, HDAC3, MDM2, or p53 were used for in vitro translation using the TNT Quick Coupled Transcription/Translation System (Promega, Madison, WI). Purified His‐ or His‐fusion protein bound to nickel beads was incubated with in vitro translated NCoR, SMRT, HDAC1, HDAC3, MDM2, or p53 for 4 h at 4 °C. After washing, the bound proteins were examined by immunoblotting.

### Glucose Uptake, Pyruvate, Lactate, ATP, HK, PFK, ALDO, PK, and LDH Assays

Glucose Uptake Colorimetric Assay Kit, Pyruvate Colorimetric Assay Kit, Lactate Assay Kit II, ATP Colorimetric Assay Kit, Hexokinase Colorimetric Assay Kit, PFK Activity Colorimetric Assay Kit, Aldolase Activity Colorimetric Assay Kit, Pyruvate Kinase Activity Colorimetric Assay Kit and Lactate Dehydrogenase Activity Assay Kit were used to measure glucose uptake, levels of pyruvate, lactate and ATP, and activities of HK, PFK, ALDO, PK and LDH, respectively, according to the manufacturer's instructions (Biovision, Palo Alto, CA). Data were normalized to cell numbers unless specified otherwise.

For the glucose uptake assay, 10 000 cells were seeded into 96‐well plates and incubated in DMEM containing 10% FBS for 10 h, at which time the cell numbers for each group were very similar. Cells were washed three times with phosphate‐buffered saline (PBS) and then subjected to glucose starvation by incubation with 100 µL Krebs‐Ringer‐Phosphate‐HEPES buffer containing 2% bovine serum albumin for 40 min. Subsequently, 10 µL of 10 mm 2‐DG, a glycolytic inhibitor, was added to the medium for 20 min. The cells were lysed with extraction buffer, frozen/thawed, heated, and neutralized with neutralization buffer. After centrifugation at 1.2 × 10^4^ rpm for 5 min, the supernatant was used to measure glucose uptake at 412 nm in a microplate reader.

For pyruvate and ATP level assays, as well as PK activity assays, cells (5 × 10^5^) were harvested and extracted with the corresponding assay buffer provided in respective kits. The cells were centrifuged, and the supernatant was assayed at 570 nm using a microplate reader. For HK and ALDO activity assays, cells (5 × 10^5^) were collected and homogenized in the corresponding assay buffer. The cells were centrifuged, and the supernatant was assessed at 450 nm using a microplate reader. For the PFK activity assay, cells (2 × 10^6^) were harvested and extracted with the PFK activity assay buffer. The cells were centrifuged, and the absorbance of the supernatant was measured at 450 nm using a microplate reader. For the LDH activity assay, cells (2 × 10^5^) were harvested and homogenized in the LDH assay buffer. The cells were centrifuged, and the absorbance of the supernatant was measured at 450 nm using a microplate reader. For the lactate level assay, 10 000 cells were seeded into 12‐well plates and incubated in DMEM containing 10% FBS for 10 h. To assess lactate secretion, the media were replaced with DMEM without FBS. After incubation for 1 h, the supernatant was collected, and lactate production was measured at 450 nm using a microplate reader. To assess lactate levels in mouse tumors, 10 mg of tumor tissue was homogenized in the assay buffer and centrifuged. The supernatant was examined and normalized to the protein concentration.

### Extracellular Acidification and Oxygen Consumption Rate Assays

The ECAR and cellular OCR were determined using a Seahorse XF^e^ 96 Extracellular Flux Analyzer (Seahorse Bioscience, Billerica, MA), according to the manufacturer's protocol. The Seahorse XF Glycolysis Stress Test Kit and Seahorse XF Cell Mito Stress Test Kit (Agilent Technologies, Santa Clara, CA) were used to measure ECAR and OCR levels, respectively. Briefly, 10 000 cells per well were plated into a Seahorse XF^e^ 96 cell culture microplate for 10 h, at which time the cell number for each group was very similar. For ECAR, glucose, the oxidative phosphorylation inhibitor oligomycin, and the glycolytic inhibitor 2‐DG were sequentially injected into each well at indicated time points. For OCR, oligomycin, the reversible inhibitor of oxidative phosphorylation p‐trifluoromethoxy carbonyl cyanide phenylhydrazone (FCCP), and the mitochondrial complex I inhibitor rotenone plus the mitochondrial complex III inhibitor antimycin A (Rote/AA) were sequentially injected. Data were assessed using Seahorse XF^e^ 96 Wave software and normalized based on cell numbers.

### Cell Proliferation and Invasion Assays

For the cell proliferation assay, cells were seeded in 96‐well plates at a density of 3000 cells per well. Cell proliferation was determined using the CCK‐8 assay, performed five times over 24 h, according to the manufacturer's instructions (Dojindo Laboratories, Kumamoto, Japan). Briefly, CCK‐8 solution was added to cultured cells in each well at 37 °C for 1 h. The OD values were measured at 450 nm using a microplate reader. The cell invasion assay was performed using Matrigel invasion chambers according to the manufacturer's protocol (BD Biosciences, Franklin Lakes, NJ). Cells were plated on the upper surface of transwell inserts. The lower surface was washed with PBS, fixed with 4% paraformaldehyde, and stained with 0.5% crystal violet. The number of invasive cells was determined in five randomly selected microscopic fields and photographed.

### Analysis of Tumor Growth and Metastasis In Vivo

Animal experiments were approved by the Institutional Animal Care Committee of the Beijing Institute of Biotechnology. The human metastatic and estrogen‐independent breast cancer cell line MDA‐MB‐231 was used to examine the role of OVOL2 in tumor growth and metastasis, and the human non‐metastatic and estrogen‐dependent breast cancer cell line MCF7 was used to examine the role of OVOL2 in tumor growth. For MCF7 cells, six‐week‐old BALB/c female nude mice were supplemented with 60‐day slow‐release estradiol pellets (0.72 mg; Innovative Research of America, Sarasota, FL). For the tumor growth assessment, 10^7^ MDA‐MB‐231 or MCF7 cells harboring different constructs were subcutaneously inoculated into the second mammary fat pad on the right side of the nude mice. The length and width of the observable tumors were measured at indicated times using calipers. Tumor volume was calculated using the following formula: volume = (longest diameter × shortest diameter^2^)/2. Mice were euthanized at indicated time points. The excised tumors were frozen in liquid nitrogen for further analysis. For lung metastasis analysis, 1–2 × 10^6^ MDA‐MB‐231 cells carrying different constructs with luciferase labels were injected into the lateral tail vein of each BALB/c female mouse. After 30 days, mice were imaged using an IVIS200 imaging system (Xenogen Corporation, Alameda, CA). After euthanasia, all lungs were harvested for metastatic foci measurement.

### Human Clinical Samples and Immunohistochemistry (IHC)

Samples of 122 female patients with primary breast cancer were obtained from the Chinese PLA General Hospital, with the informed consent of patients and approval of the Institutional Review Committees of the Chinese PLA General Hospital (80106). The clinical features of patients are presented in Table S5, Supporting Information. Similar experiments were previously performed to estimate the sample size. No patient with breast cancer received chemotherapy or radiation therapy prior to surgery. Of the 122 patients, ^18^FDG PET scans of 42 patients were analyzed. The age of the 122 patients ranged between 30 and 83 years (mean age: 53.8 years), and that of patients selected for analyzing ^18^FDG PET scans ranged from 32 to 72 (mean age: 50.4 years). For the clinical outcome analysis, the follow‐up time was 3–148 months (mean: 74.5 months). Normal distribution was performed using SPSS13.0.

IHC analysis of formalin‐fixed paraffin‐embedded specimens was performed as previously described.^[^ ^60^ ^]^ Briefly, formalin‐fixed paraffin samples were deparaffinized, rehydrated, and pre‐treated with 3% H_2_O_2_ for 20 min. Antibody‐binding epitopes of the antigens were retrieved by microwave treatment, and sections were preincubated with 10% goat serum to block nonspecific binding. Rabbit anti‐OVOL2 (PA5‐115700, Invitrogen), rabbit anti‐PKM2 (4053S, Cell Signaling Technology) and rabbit anti‐LDHA (19987‐1‐AP, Proteintech), diluted 1:100, 1:200 and 1:200, respectively, were used as the primary antibodies. The sections were incubated with primary antibodies for 1 h at room temperature, followed by the addition of biotinylated anti‐rabbit secondary antibody and streptavidin‐horseradish peroxidase. DAB was used as a chromogen, and hematoxylin was used for counterstaining. Immunoreactivity for OVOL2, PKM2, and LDHA was scored by multiplying the percentage of stained cells in 5% increments (0, 5, 10, …) by the staining intensity (low, 1+; medium, 2+; strong, 3+). The optimal cut‐off value of the IHC scores was estimated using receiver operating characteristic curve analysis. We defined the scores ≤0.85 and >0.85 as low and high OVOL2, respectively.

### Statistical Analysis

Trial experiments or similar experiments performed previously were used to estimate the sample size with adequate statistical power. Data are presented as mean ± standard deviation (SD) (*n* = 3, unless otherwise specified). The Student's *t*‐test was used to compare the means of two groups. When more than two groups were compared, a one‐way ANOVA was performed. Comparisons were performed using independent sample *t*‐test and Mann–Whitney U tests for parametric and non‐parametric data, respectively. DFS and OS were estimated using the Kaplan–Meier method, and differences between survival curves were examined using the log‐rank test. All statistical tests were two‐sided. Statistical analyses were performed using SPSS Statistics 21 and GraphPad Prism 9. A *p*‐value of <0.05 was considered statistically significant.

## Conflict of Interest

The authors declare no conflict of interest.

## Author Contributions

X.Z., F.L., S.L., L.L., X.R., and J.L. contributed equally to this work. Q.Y. conceived the project, designed the experiments, analyzed the data, and wrote the manuscript. X.Z., F.L., S.L., L.L., X.R., and J.L. designed the experiments and analyzed the data, aided by Y.L., C.M., D.Z., T.Y., Y.L. and B.J. L.D., and S.G. analyzed the data.

## Supporting information

Supporting Information

Supporting Information

Supporting Information

Supporting Information

Supporting Information

## Data Availability

The data that support the findings of this study are available in the supplementary material of this article.
