# Correction to “Transcriptional Repression of Aerobic Glycolysis by OVOL_2_ in Breast Cancer”

**DOI:** 10.1002/advs.75526

**Published:** 2026-05-08

Zhang X, Luo F, Luo S, Li L, Ren X, Lin J, Liang Y, Ma C, Ding L, Zhang D, Ye T, Lin Y, Jin B, Gao S, Ye Q. Transcriptional Repression of Aerobic Glycolysis by OVOL2 in Breast Cancer. Adv Sci (Weinh). 2022, 9(27):e2200705. https://doi.org/10.1002/advs.202200705

In the originally published version of this article, errors were identified in Figures 3G, 5D, 7A, and Figure S8C. In Figure 3G, the Western blot image for ALDOA was incorrectly reused from Figure S3D. In Figure 5D, the images for the group labeled “WT + shCtrl + 2‐DG” were inadvertently replaced with images corresponding to the second group in Figure 5B. In Figure 7A, the image designated for “case 1” was mistakenly applied to ‘case 2’. In Figure S8C, the image labeled “Normal IgG” was incorrectly applied to the “α‐LDHA + GST‐LDHA” lane. The correct figures are presented below. These changes do not alter the conclusions of the article.


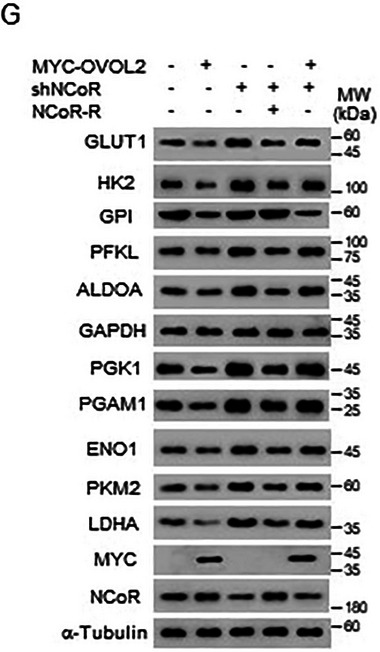


Figure 3G.


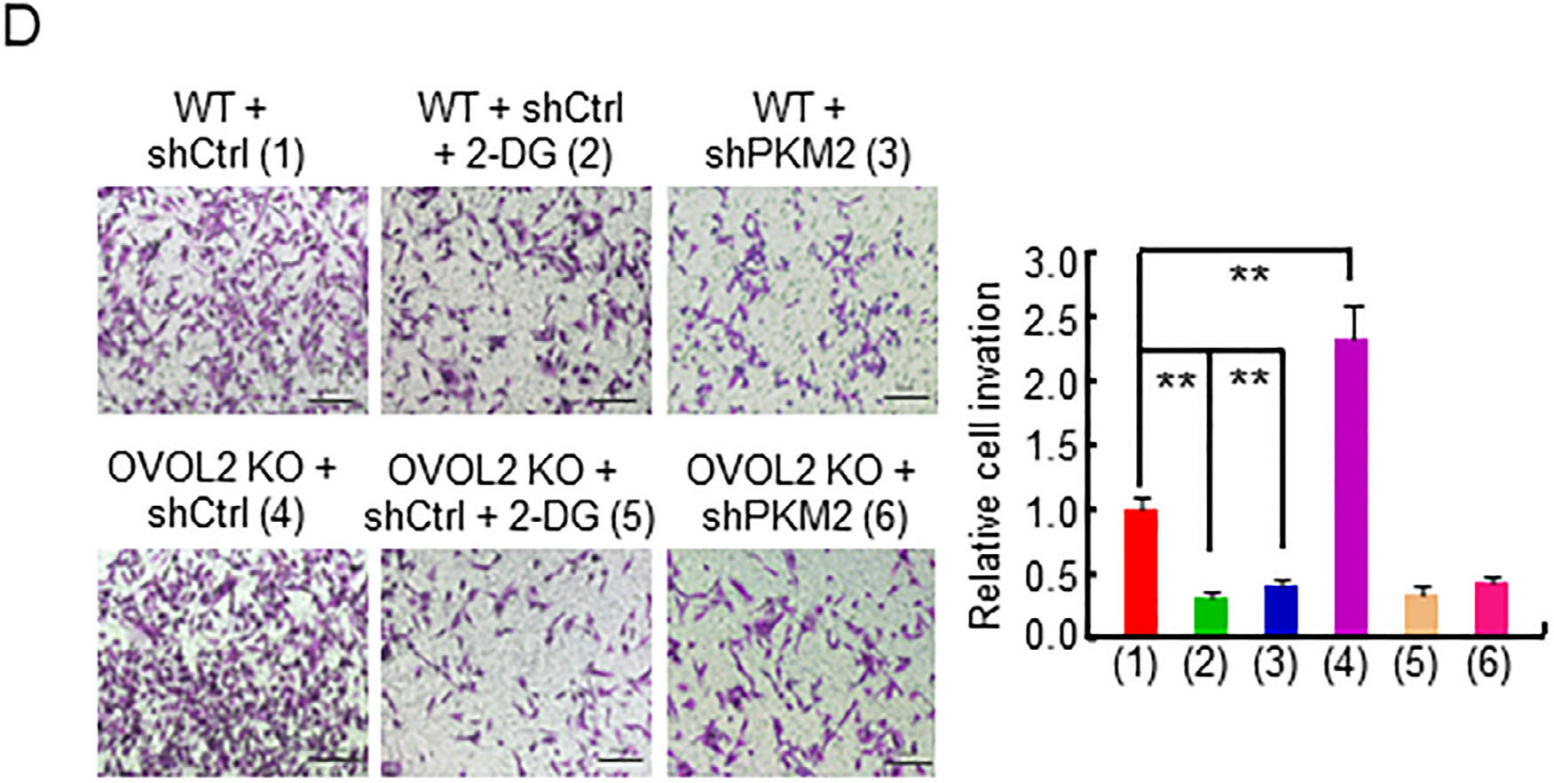


Figure 5D.


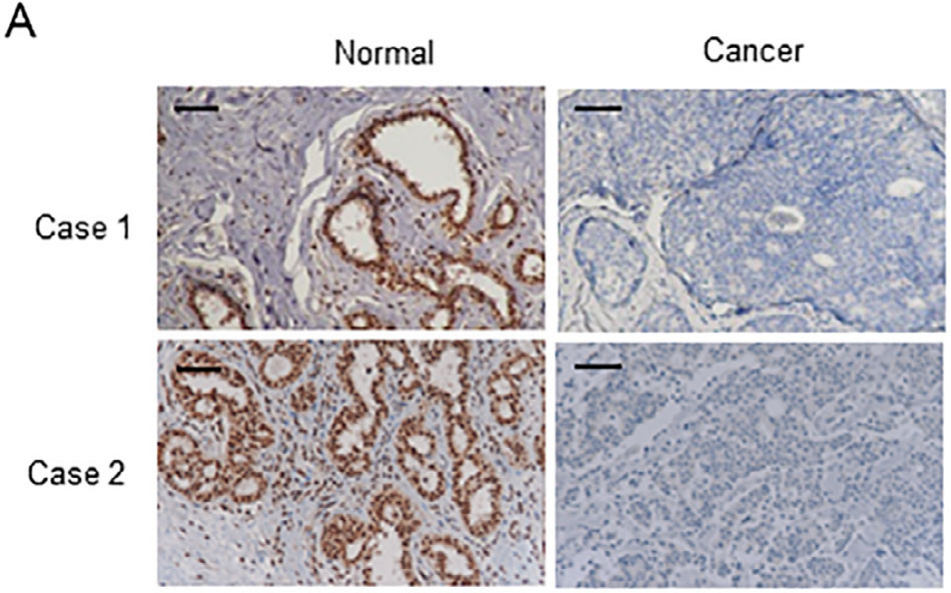


Figure 7A.


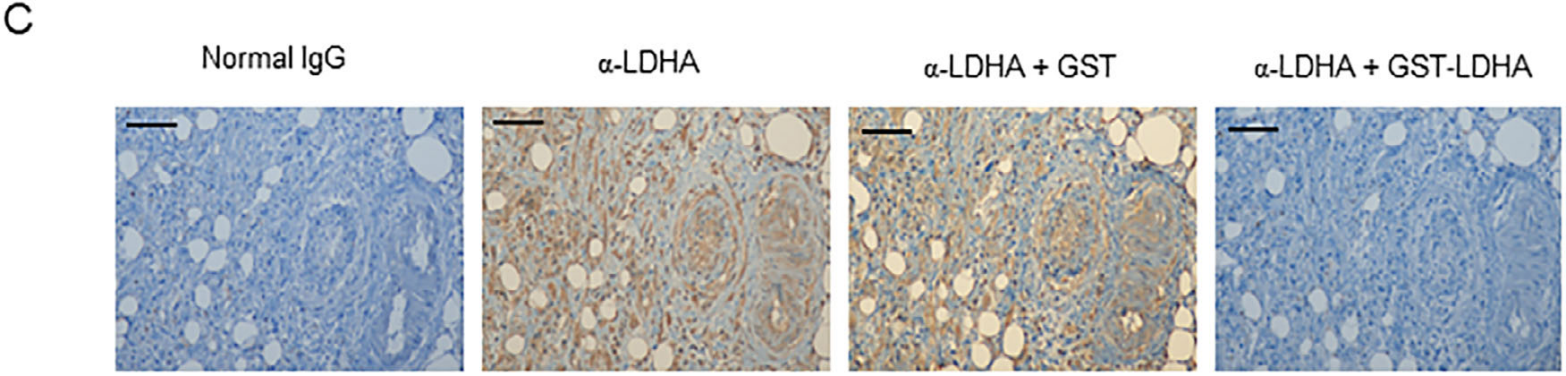


Supplementary Figure S8C

We apologize for these errors.

