# Supplementary material for: Transcriptional Repression of Aerobic Glycolysis by OVOL2 in Breast Cancer
Source: Adv Sci (Weinh). 2022 Jul 27;9(27):2200705. doi: 10.1002/advs.202200705 (PMC9507357; doi:10.1002/advs.202200705)
Supplement: Supplementary file 1 — Supporting Information [file ADVS-9-2200705-s004.pdf]

ADVANCED SCIENCE
Open Access

Supporting Information

for *Adv. Sci.*, DOI 10.1002/advs.202200705

Transcriptional Repression of Aerobic Glycolysis by OVOL2 in Breast Cancer

*Xiujuan Zhang, Fei Luo, Shaliu Luo, Ling Li, Xinxin Ren, Jing Lin, Yingchun Liang, Chao Ma, Lihua Ding, Deyu Zhang, Tianxing Ye, Yanni Lin, Bilian Jin, Shan Gao and Qinong Ye**

Supplementary Table 1. Potential OVOL2-interacting proteins identified by Co-IP and mass spectrometry

Protein name	Description	Mass (Dalton)	Score
NCoR	Nuclear receptor corepressor	270212	126
β-catenin	Catenin beta-1	86069	421
α-catenin	Catenin alpha-1	100693	127
δ-catenin	Catenin delta-1	108674	38
MDM2	Murine double minute 2, human homologue	56323	89
HDAC1	Histone deacetylase 1	55638	97
HDAC3	Histone deacetylase 3	48848	106
SVIL	Supervillin	249417	791
ZO1	Tight junction protein ZO-1	195682	671
LIMA1	LIM domain and actin-binding protein 1	85630	436
PRMT5	Protein arginine N-methyltransferase 5	73322	312
RPS27A	Ubiquitin-40S ribosomal protein S27a	18296	205
FLII	Protein flightless-1 homolog	146142	182
HSP90AA1	Heat shock protein HSP 90-alpha	85006	153
SCYL2	SCY1-like protein 2	104327	101
EFHD2	EF-hand domain-containing protein D2	26794	96
MISP	Mitotic interactor and substrate of PLK1	75482	85
TMOD3	Tropomodulin-3	39741	84
EPSTI1	Epithelial-stromal interaction protein 1	36942	79
SIPA1L1	Signal-induced proliferation-associated 1-like protein 1	201102	79
N4BP3	NEDD4-binding protein 3	60889	72
TRIP11	Thyroid receptor-interacting protein 11	228131	48
THRAP3	Thyroid hormone receptor-associated protein 3	108658	195
BCLAF1	Bcl-2-associated transcription factor 1	106173	95
SMARCA5	SWI/SNF-related matrix-associated actin-dependentregulator of chromatin subfamily A member 5	122513	79
MEP50	Methylosome protein 50	37442	71
PPP1R12A	Protein phosphatase 1 regulatory subunit 12A	115610	69
IPO9	Importin-9	116858	51
NEB2	Neurabin-2	89309	120
IMB1	Importin subunit beta-1	98420	81
SSRP1	FACT complex subunit SSRP1	81367	60
UNC45A	Protein unc-45 homolog A	104266	308
MIC60	MICOS complex subunit MIC60	84026	239
MCM7	DNA replication licensing factor MCM7	81884	226
NUP93	Nuclear pore complex protein Nup93	93943	196

XRCC5	X-ray repair cross-complementing protein 5	83222	190
DHX15	Pre-mRNA-splicing factor ATP-dependent RNA helicase DHX15	91673	174
IQGAP1	Ras GTPase-activating-like protein IQGAP1	189761	101
MLH1	DNA mismatch repair protein Mlh1	85175	76
STAT1	Signal transducer and activator of transcription1-alpha/beta	87850	74
CAND2	Cullin-associated NEDD8-dissociated protein 2	136653	45
PPM1B	Protein phosphatase 1B	53180	44
LSR	Lipolysis-stimulated lipoprotein receptor	72534	396
HSPA9	Stress-70 protein	73920	334
AIFM1	Apoptosis-inducing factor 1	67144	196
TOM1L2	TOM1-like protein 2	55864	174
DDX5	Probable ATP-dependent RNA helicase DDX5	69618	137
DDX17	Probable ATP-dependent RNA helicase DDX17	80906	85
KARS	Lysine--tRNA ligase	68461	79
IVNS1ABP	Influenza virus NS1A-binding protein	72937	73
RUVBL1	RuvB-like 1	50538	117
DDX39A	ATP-dependent RNA helicase DDX39A	49611	85
CAMK2D	Calcium/calmodulin-dependent protein kinase type IIsubunit delta	56961	56
GIPC1	PDZ domain-containing protein GIPC1	36141	225
HNRNPH3	Heterogeneous nuclear ribonucleoprotein H3	36960	126
H2AFY	Core histone macro-H2A.1	39764	59
H4	Histone H4	11360	53
DCAF7	DDB1- and CUL4-associated factor 7	39528	51
PID1	PTB-containing, cubilin and LRP1-interacting protein	28766	41
TRA2A	Transformer-2 protein homolog alpha	32726	167
HNRNPA3	Heterogeneous nuclear ribonucleoprotein A3	39799	149
HNRNPA1	Heterogeneous nuclear ribonucleoprotein A1	38837	148
POLDIP2	Polymerase delta-interacting protein 2	42235	119
U2AF1	Splicing factor U2AF 35 kDa subunit	28368	86
DCD	Dermcidin	11391	66
RALY	RNA-binding protein Raly	32501	55
H3F3C	Histone H3.3C	15318	46
CDK9	Cyclin-dependent kinase 9	43149	45
ARF4	ADP-ribosylation factor 4	20612	137
AIF1L	Allograft inflammatory factor 1-like	17114	132
ARF1	ADP-ribosylation factor 1	20741	118
ARF3	ADP-ribosylation factor 3	20645	118
JAGN1	Protein jagunal homolog 1	21111	97
TAF12	Transcription initiation factor TFIID subunit 12	18027	76

UBE2V2	Ubiquitin-conjugating enzyme E2 variant 2	16409	74
RBM3	RNA-binding protein 3	17160	70
TMED2	Transmembrane emp24 domain-containing protein 2	22860	70
H2B1B	Histone H2B type 1-B	13942	69
NME2	Nucleoside diphosphate kinase B	17401	65
ARL8B	ADP-ribosylation factor-like protein 8B	21753	62
CDC42	Cell division control protein 42 homolog	21587	50
CALM1	Calmodulin	16827	478
ARF5	ADP-ribosylation factor 5	20631	220
SEC11A	Signal peptidase complex catalytic subunit SEC11A	20612	80
REEP5	Receptor expression-enhancing protein 5	21707	76
LAMTOR1	Ragulator complex protein LAMTOR1	17848	69
PDZD11	PDZ domain-containing protein 11	16121	69
ARF6	ADP-ribosylation factor 6	20183	66
TCP4	Activated RNA polymerase II transcriptional coactivatorp15	14386	63
PDCD6	Programmed cell death protein 6	21912	62
NRBF2	Nuclear receptor-binding factor 2	32529	55
H2AV	Histone H2A.V	13501	49
H2AZ	Histone H2A.Z	13545	49

Supplementary Table 2. The cDNA target sequences of shRNAs or siRNAs.

Gene	Target sequence (5'→3')
p53 (siRNA/shRNA)	AAGACUCCAGUGGUAAUCUAC
MDM2 (siRNA/shRNA)	GCUUCGGAACAAGAGACUC
PKM2 (shRNA)	CCAUAAUCGUCCUCACCAA
LDHA (shRNA)	GGAGAAAGCCGUCUUAAUU
NCoR (shRNA)	GCUCCUCUCAGCACAGUAU
HDAC3 (shRNA)	CCAAGAGUCUUAAUGCCUU

Supplementary Table 3. Primers used for real-time PCR.

Gene	Species	Forward (5'→3')	Reverse (5'→3')
GLUT1	Human	CATCCCATGGTTCATCGTGGCTGAACT	GAAGTAGGTGAAGATGAAGAACAGAAC
HK2	Human	GCCATCCTGCAACACTTAGGGCTTGAG	GTGAGGATGTAGCTTGTAGAGGGTCCC
GPI	Human	TATTGTGTTCACCAAGCTCACACC	TGGTAGAAGCGTCGTGAGAGGTC
PFKL	Human	GGAGAAGCTGCGCGAGGTTTAC	ATTGTGCCAGCATCTTCAGCATGAG
ALDOA	Human	AGGCCATGCTTGCACTCAGAAGT	AGGGCCCAGGGCTTCAGCAGG
GAPDH	Human	TTCCGTGTCCCCACTGCCAACGT	CAAAGGTGGAGGAGTGGGTGTCGC
PGK1	Human	ATGTCGCTTTCTAACAAGCTGA	GCGGAGGTTCTCCAGCA
PGAM1	Human	GGAAACGTGTACTGATTGCAGCCC	TTCCATGGCTTTGCGCACCGTCT
ENO1	Human	GACTTGGCTGGCAACTCTG	GGTCATCGGGAGACTTGAA
PKM2	Human	GCCCGTGAGGCAGAGGCTGC	TGGTGAGGACGATTATGGCCC
LDHA	Human	ATGGCAACTCTAAAGGATCA	GCAACTTGCAGTTCGGGC
α-Tubulin	Human	CCAAGCTGGAGTTCTCTA	CAATCAGAGTGCTCCAGG
ASCL1	Human	CGCGTCAAGTTGGTCAACCTG	GGCCATGGAGTTCAAGTCGTTG
CREB3L1	Human	TTGATGACCCTGTGCTGGATGAG	GCTCCTGCTTCACCATGATGGAG
OVOL2	Human	TCACCTCAAGTGCCACAACCAG	CCGCTGCTTATAGGCATACTGC
MSX1	Human	TCAAGCTGCCAGAAGATGCGC	GGTACTGCTTCTGGCGGAACTTG
MSX2	Human	CTCATGTCCGACAAGAAGCCG	CGGCTTCCGATTGGTCTTGTG
ATF3	Human	TCTGCGCTGGAATCAGTCACTG	GAGCCTTCAGTTCAGCATTCACAC
KLF7	Human	CCAGGAGCTACAACTTGTCCAC	TCTTCTCACAGATGGCCGCTTC
GLUT1	Mouse	CATGGTTCATTGTGGCCGAGCTGT	TCGGCCTTTGGTCTCAGGGACTTT
HK2	Mouse	CCTGCTACAGGTCCGAGCCATCTT	GAGGATGAAGCTTGTACAGTGTCC
GPI	Mouse	AACCGGCCGACCAACTCAAT TGTG	TGCCGTCCAGCTCTGGCTCAATTT
PFKL	Mouse	AATGTGCTGGGCCACTTGCAGCAG	TGACCGGACTGAAGGCCACTACCT
ALDOA	Mouse	ATGAGGAGATTGCCATGGCAACGG	TTTAGAGCAGAGGCCTGCAGGGCT
GAPDH	Mouse	GGGACAAGGATAGTCATTTTGGGG	TGTCATTGAGAGCAATGCCAGCCC
PGK1	Mouse	AGACTGGCCAAGCTACTGTGGCCT	GAAGTGGCTTTCACCACCTCATCC
PGAM1	Mouse	TACGCAGACCTTACTGAAGACCAG	AGCTCCATGATGGCCTCTTCTGAG
ENO1	Mouse	ACCAACCCTAAGCGGATTGCCAAG	AGTCTTGATCTGCCCAGTGCAGAG
PKM2	Mouse	TCGCATGCAGCACCTGATT	CCTCGAATAGCTGCAAGTGGTA
LDHA	Mouse	GCAGACAAGGAGCAGTGGAAGGAG	ACACTGAGGAAGACATCCTCATTG
α-Tubulin	Mouse	AACCAGATGGTGAAATGTGACCCT	CACAGTGGGAGGCTGGTAGTTAAT

Supplementary Table 4. Primers used for ChIP.

Gene	Forward (5'→3')	Reverse (5'→3')
GLUT1/2	GTGAAACCCCGTCTCTACTAA	TCTCAGACAGAGTCTCGCTCTG
GLUT3	TTGGTCTTTCTACAACCCTACGAG	CTTCTCTAGCAAAATGGTGGAGCC
HK2-1	GAAATAAGCCTGCAACTCCAGGA	GAGTCCCTGTTTGCTCTATTGG
HK2-2	AGAGGGTGGAGGGCAGCCCCGAAT	GGGATGTTGAGGACAGTTAATCAC
GPI	GACAAGGTGTTGCTGTGTCA	AGGTGGGATAGCTTGACG
PFKL-1	GCCATCCTGTTTTCCACAGCAGCT	AGGCAGAGGTTGCAGTGAGTGGAG
PFKL-2	TGCCTCAGCCTCCTGCATAGCT	ATAATCCCAGAACTTTGGG
ALDOA-1	TCATGCCAGTAATCCTAGCAC	CTCCTGGGTTCAAGCGATT
ALDOA-2	AGCACTTTGGGAAGCTGAG	TGTTCAAGTGATTCACCTGCC
ALDOA-3	GACAGAGCGAGACTCCGTCTCA	GACAGAGCGAGACTCCGTCTCA
GAPDH	GGTCCCAGCTTAGGTTCATCAGGT	TGGCCAGGAAGACGCTTGAAAAGG
PGK1-1	TGAAACTCTGGTCTCCCGC	ACGAAAGTGTAACCGCCCA
PGK1-2	AGCGGCGCCGACCCTGGGTCT	CGAAGATCCGGGTGACGCTGC
PGK1-3	CGCAGCGTCACCCGGATCTTCG	TTTGTCACGTCCGGCACGCCGC
PGK1-4	AAGGTTCCTTGCGGTTCGC	TGCTGAGCAGCCGCTATTG
PGAM1-1	GGCACTGTGGCTCACGCTGGTAAT	GTTTAAGCAATTCTGCTGCCTCAG
PGAM1-2	AGATCGCACCACTGCACTCCAGTCT	CTTTCACACACGTTAAGTCAGTGC
ENO1-1	TCCACGGAATATGACCCGT	CACGTGCTTCCCCAGTGTT
ENO1-2	CCAGCGCCCCAACCCCGGAGTG	CCCTCGACCTTGCTGACAACTTTG
PKM2-1	TTATTCACATGGCCCCAACC	AGTAGGGAGAACTAGGGACT
PKM2-2	TCCGAAGGCGGCCAGGACCTCCAA	ACTCCTCCGGGTGTCTGCTCTGG
LDHA-1	TGGAATCAGCAAGAATACAGGCCC	CTGAGAAGATGGGATTACAGCCAG
LDHA-2	TGAGGCAGGAGATTCGCTTGAACC	TTCTGGCTCAAGCGATCCTCCCA
GLUT1-Upstream	TGCATTTGCAGAGGGTCGGA	ACAAGAGGGGATAGCAGGGG
HK2-Upstream	TGATTATTATCCGGTTCACATGAAA	AAATGAAGAGGAGGATGCCTGC
GPI-Upstream	ACCGTGCCCGGCCTCATTTCAC	CTACCAACACGGCCACTACTCC
PFKL-Upstream	TCACTCTGTCCCGCAGCACA	GCTGTTTACAGCCGAGCACT
ALDOA-Upstream	TCACACCTGTAATCCCAGCA	GTGTGATCTCAGCTCACTGC
PGK1-Upstream	GCCAACAAGGGTGTCTCTCTAGAG	CAGAAGCAACCCCCAGTGATCCAC
PGAM1-Upstream	AGGCAAGTCTTCCCCTACCA	GAGGCAGAGGTTGCAGTGAG
ENO1-Upstream	AAGCGGGATGTGGGTACACA	TGTGAAAGAGGGTCTCCAGC
PKM2-Upstream	CTTCTGAAATGGGGGTCTTGCAAC	AGCAGGCCTCAGAGAACAGAATTC
LDHA-Upstream	GTAGTCCCAGTCACTTCGGG	CTGCACTTTCTACTGCCCAC

Supplementary Table 5. Clinicopathological data of breast cancer samples.

	No. of cases	No. of disease-free survival (%)	No. of overall survival (%)
Age years			
<40	14	6 (18.2%)	8 (9.0%)
40-49	47	6 (18.2%)	41 (46.1%)
50-59	20	7 (21.2%)	13 (14.6%)
60-69	20	5 (15.1%)	15 (16.8%)
≥70	21	9 (27.3%)	12 (13.5%)
Total	122	33 (100.0%)	89 (100.0%)
ER			
Negative	38	12 (36.4%)	26 (29.2%)
Positive	84	21 (63.6%)	63 (70.8%)
Total	122	33 (100.0%)	89 (100.0%)
PR			
Negative	55	19 (57.6%)	36 (40.4%)
Positive	67	14 (42.4%)	53 (59.6%)
Total	122	33 (100.0%)	89 (100.0%)
HER2			
Negative	97	22 (66.7%)	75 (84.3%)
Positive	25	11 (33.3%)	14 (15.7%)
Total	122	33 (100.0%)	89 (100.0%)
