# Supplementary material for: Transcriptional Repression of Aerobic Glycolysis by OVOL2 in Breast Cancer
Source: Adv Sci (Weinh). 2022 Jul 27;9(27):2200705. doi: 10.1002/advs.202200705 (PMC9507357; doi:10.1002/advs.202200705)
Supplement: Supplementary file 2 — Supporting Information [file ADVS-9-2200705-s005.ppt]

## Slide 1
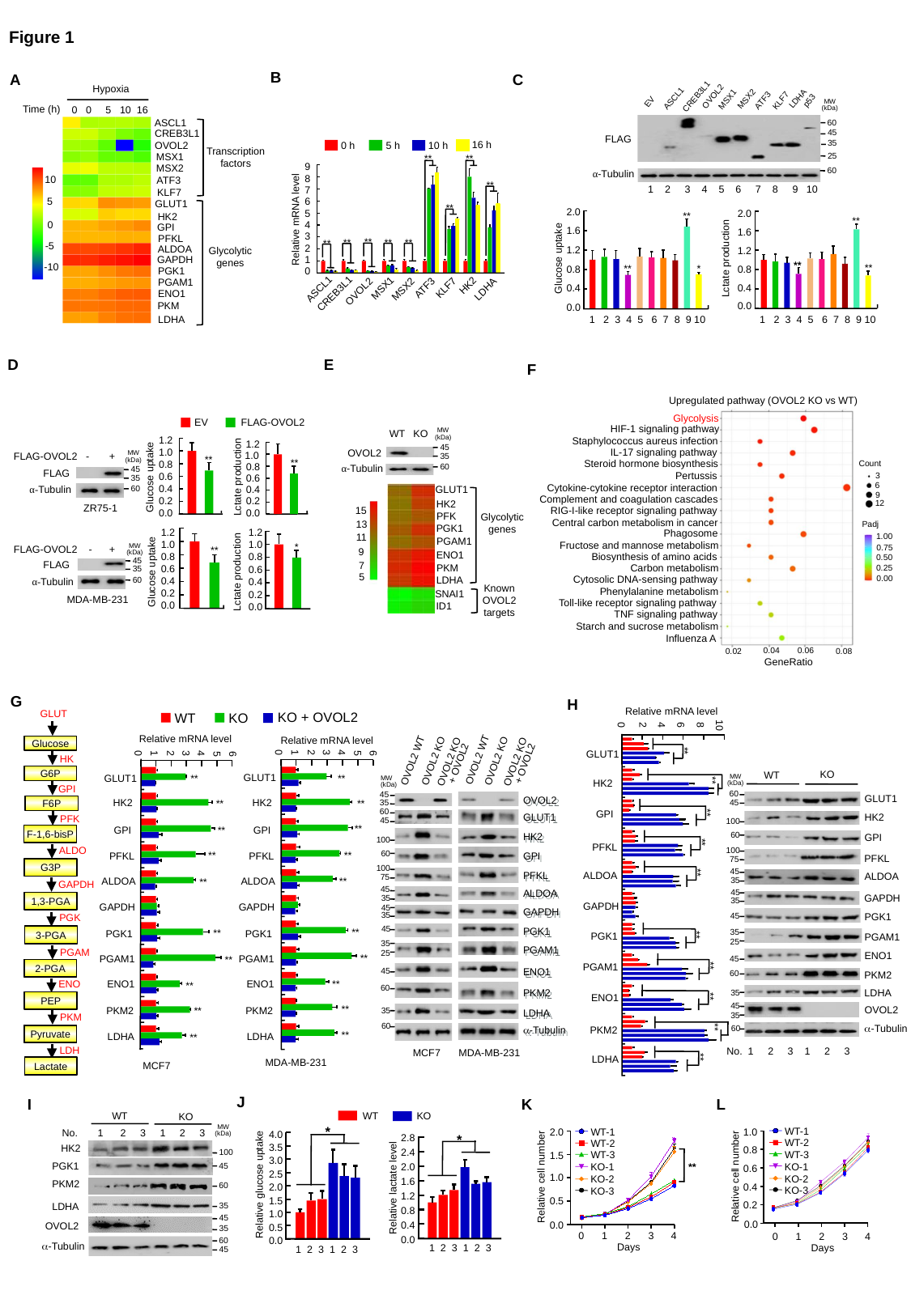

Figure 1
B
A
C
Hypoxia
OVOL2
CREB3L1
MSX2
ASCL1
MSX1
ATF3
KLF7
p53
LDHA
EV
MW
(kDa)
Time (h)
0 0 5 10 16
ASCL1
60
CREB3L1
45
FLAG
35
OVOL2
16 h
0 h
10 h
5 h
Transcription
factors
MSX1
25
**
**
Relative mRNA level
MSX2
60
9
α-Tubulin
10
ATF3
8
**
1 2 3 4 5 6 7 8 9 10
KLF7
7
5
GLUT1
6
**
**
**
**
**
**
*
HK2
2.0
5
2.0
Glucose uptake
Lctate production
0
GPI
4
1.6
1.6
PFKL
**
**
**
3
**
**
-5
ALDOA
Glycolytic genes
2
1.2
1.2
GAPDH
**
1
-10
PGK1
0.8
0.8
0
PGAM1
HK2
ENO1
KLF7
0.4
MSX1
ATF3
0.4
ASCL1
MSX2
LDHA
OVOL2
CREB3L1
PKM
0.0
0.0
LDHA
1 2 3 4 5 6 7 8 9 10
1 2 3 4 5 6 7 8 9 10
D
E
F
Upregulated pathway (OVOL2 KO vs WT)
Glycolysis
EV
FLAG-OVOL2
HIF-1 signaling pathway
MW
(kDa)
WT KO
Glucose uptake
Staphylococcus aureus infection
Lctate production
1.2
45
**
1.2
IL-17 signaling pathway
OVOL2
**
FLAG-OVOL2 - +
MW
(kDa)
35
1.0
1.0
Steroid hormone biosynthesis
Count
α-Tubulin
60
0.8
45
0.8
FLAG
Pertussis
3
35
0.6
0.6
6
Cytokine-cytokine receptor interaction
GLUT1
α-Tubulin
60
0.4
0.4
9
Complement and coagulation cascades
HK2
12
0.2
0.2
ZR75-1
RIG-I-like receptor signaling pathway
15
PFK
Glycolytic genes
0.0
0.0
Central carbon metabolism in cancer
13
Padj
PGK1
Phagosome
Glucose uptake
11
Lctate production
1.00
1.2
1.2
**
PGAM1
*
Fructose and mannose metabolism
MW
(kDa)
0.75
FLAG-OVOL2 - +
1.0
1.0
9
ENO1
Biosynthesis of amino acids
0.50
45
0.8
0.8
FLAG
7
PKM
Carbon metabolism
0.25
35
0.6
0.6
5
Cytosolic DNA-sensing pathway
LDHA
0.00
α-Tubulin
60
0.4
0.4
Known OVOL2 targets
Phenylalanine metabolism
SNAI1
0.2
MDA-MB-231
0.2
Toll-like receptor signaling pathway
ID1
0.0
0.0
TNF signaling pathway
Starch and sucrose metabolism
Influenza A
0.04
0.06
0.08
0.02
GeneRatio
G
H
GLUT
Relative mRNA level
KO + OVOL2
WT
KO
0
2
4
6
8
10
Relative mRNA level
Relative mRNA level
Glucose
GLUT1
**
HK
OVOL2 KO
OVOL2 KO
0
1
2
3
4
5
6
0
1
2
3
4
5
6
OVOL2 WT
OVOL2 KO
 + OVOL2
OVOL2 KO
 + OVOL2
OVOL2 WT
KO
WT
**
G6P
**
MW
(kDa)
MW
(kDa)
HK2
GLUT1
GLUT1
**
GPI
60
45
GLUT1
OVOL2
**
**
35
45
F6P
HK2
HK2
60
GPI
HK2
GLUT1
**
PFK
45
100
**
**
GPI
GPI
HK2
60
F-1,6-bisP
GPI
100
PFKL
**
ALDO
100
**
60
**
GPI
PFKL
75
PFKL
PFKL
100
G3P
45
ALDOA
PFKL
ALDOA
**
75
**
**
35
GAPDH
ALDOA
ALDOA
45
ALDOA
45
GAPDH
35
35
1,3-PGA
GAPDH
45
GAPDH
GAPDH
GAPDH
35
PGK1
45
PGK
45
PGK1
**
**
35
PGK1
PGAM1
3-PGA
PGK1
PGK1
**
25
35
PGAM1
PGAM
25
ENO1
**
**
45
PGAM1
PGAM1
PGAM1
**
ENO1
2-PGA
45
PKM2
60
**
ENO
**
ENO1
ENO1
60
PKM2
LDHA
35
ENO1
**
PEP
45
**
OVOL2
**
35
LDHA
PKM2
PKM2
35
PKM
60
α-Tubulin
PKM2
α-Tubulin
60
**
**
Pyruvate
**
LDHA
LDHA
LDH
No. 1 2 3 1 2 3
MCF7
MDA-MB-231
LDHA
**
MDA-MB-231
Lactate
MCF7
J
K
L
I
WT
KO
WT
KO
*
MW
(kDa)
Relative glucose uptake
No. 1 2 3 1 2 3
*
WT-1
WT-2
WT-3
KO-1
KO-2
KO-3
WT-1
WT-2
WT-3
KO-1
KO-2
KO-3
1.0
2.0
Relative lactate level
4.0
2.8
HK2
3.5
100
0.8
2.4
1.5
PGK1
3.0
**
45
2.0
0.6
Relative cell number
Relative cell number
2.5
1.0
PKM2
60
1.6
0.4
2.0
1.2
LDHA
1.5
0.5
35
0.2
0.8
45
1.0
OVOL2
0.0
35
0.4
0.0
0.5
60
0
1
2
3
4
0
1
2
3
4
0.0
α-Tubulin
0.0
Days
Days
1 2 3 1 2 3
1 2 3 1 2 3
45

## Slide 2
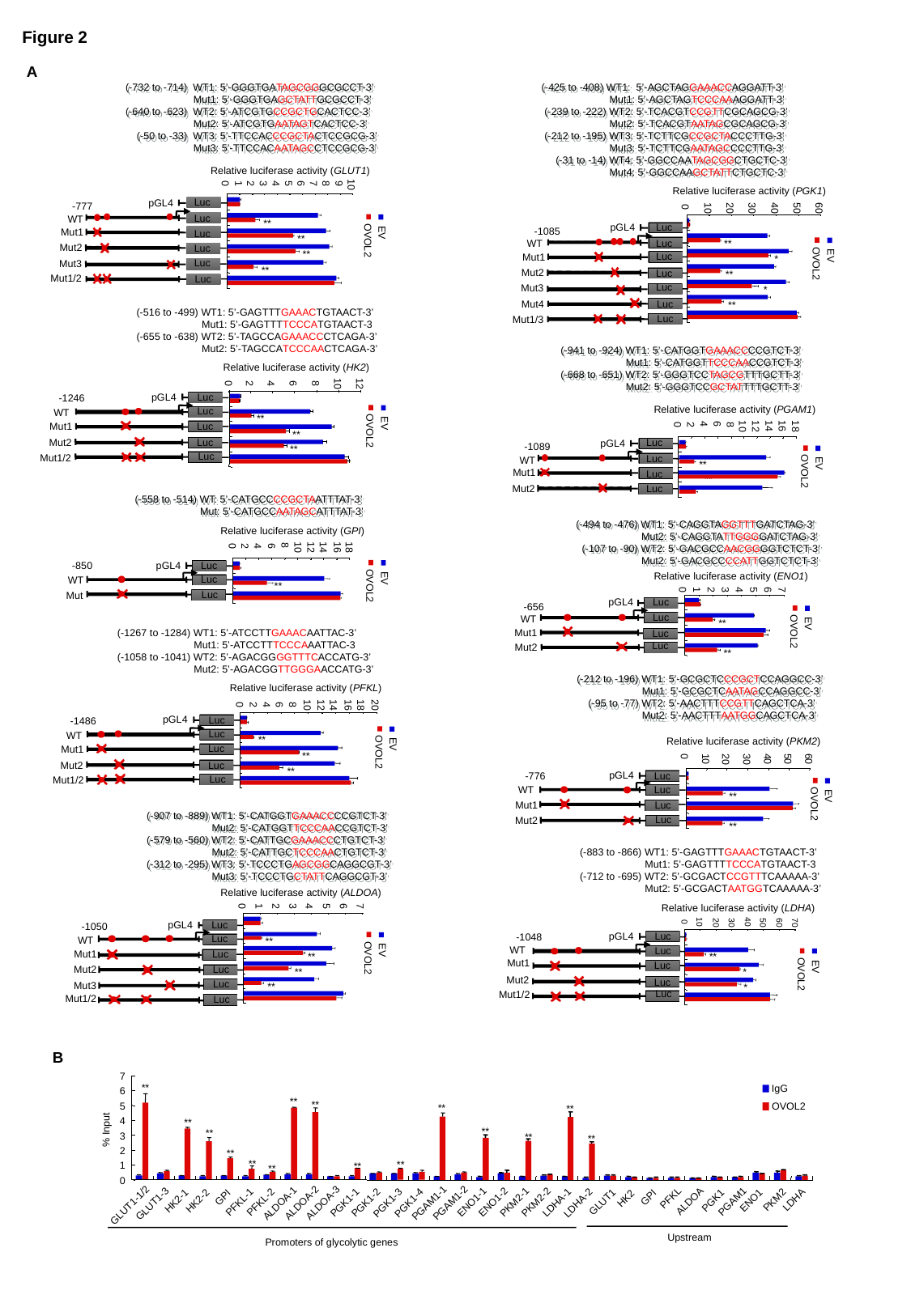

Figure 2
A
(-732 to -714) WT1: 5’-GGGTGATAGCGGGCGCCT-3’
 Mut1: 5’-GGGTGAGCTATTGCGCCT-3’
(-640 to -623) WT2: 5’-ATCGTGCCGCTGCACTCC-3’
 Mut2: 5’-ATCGTGAATAGTCACTCC-3’
 (-50 to -33) WT3: 5’-TTCCACCCGCTACTCCGCG-3’
 Mut3: 5’-TTCCACAATAGCCTCCGCG-3’
 (-425 to -408) WT1: 5’-AGCTAGGAAACCAGGATT-3’
 Mut1: 5’-AGCTAGTCCCAAAGGATT-3’
 (-239 to -222) WT2: 5’-TCACGTCCGTTCGCAGCG-3’
 Mut2: 5’-TCACGTAATAGCGCAGCG-3’
 (-212 to -195) WT3: 5’-TCTTCGCCGCTACCCTTG-3’
 Mut3: 5’-TCTTCGAATAGCCCCTTG-3’
 (-31 to -14) WT4: 5’-GGCCAATAGCGGCTGCTC-3’
 Mut4: 5’-GGCCAAGCTATTCTGCTC-3’
Relative luciferase activity (GLUT1)
Relative luciferase activity (PGK1)
0
1
2
3
4
5
6
7
8
9
10
Luc
pGL4
-777
0
10
20
30
40
50
60
Luc
WT
**
EV
OVOL2
Luc
pGL4
-1085
Mut1
Luc
**
**
Luc
WT
EV
OVOL2
Mut2
Luc
**
Luc
Mut1
*
Luc
Mut3
**
Mut2
Luc
**
Mut1/2
Luc
Luc
Mut3
*
Mut4
Luc
**
(-516 to -499) WT1: 5’-GAGTTTGAAACTGTAACT-3’
 Mut1: 5’-GAGTTTTCCCATGTAACT-3
(-655 to -638) WT2: 5’-TAGCCAGAAACCCTCAGA-3’
 Mut2: 5’-TAGCCATCCCAACTCAGA-3’
Luc
Mut1/3
 (-941 to -924) WT1: 5’-CATGGTGAAACCCCGTCT-3’
 Mut1: 5’-CATGGTTCCCAACCGTCT-3’
 (-668 to -651) WT2: 5’-GGGTCCTAGCGTTTGCTT-3’
 Mut2: 5’-GGGTCCGCTATTTTGCTT-3’
Relative luciferase activity (HK2)
0
2
4
6
8
10
12
pGL4
Luc
-1246
Relative luciferase activity (PGAM1)
Luc
WT
EV
OVOL2
**
Luc
Mut1
4
6
0
2
8
14
16
10
12
18
**
Mut2
Luc
Luc
pGL4
-1089
**
EV
OVOL2
Luc
Mut1/2
Luc
WT
**
Mut1
Luc
**
Mut2
Luc
(-558 to -514) WT: 5’-CATGCCCCGCTAATTTAT-3’
 Mut: 5’-CATGCCAATAGCATTTAT-3’
(-494 to -476) WT1: 5’-CAGGTAGGTTTGATCTAG-3’
 Mut2: 5’-CAGGTATTGGGGATCTAG-3’
 (-107 to -90) WT2: 5’-GACGCCAACGGGGTCTCT-3’
 Mut2: 5’-GACGCCCCATTGGTCTCT-3’
Relative luciferase activity (GPI)
8
0
4
6
2
10
12
14
16
18
pGL4
-850
Luc
EV
OVOL2
Relative luciferase activity (ENO1)
Luc
WT
**
Luc
Mut
0
1
2
3
4
5
6
7
pGL4
Luc
-656
EV
OVOL2
Luc
WT
**
(-1267 to -1284) WT1: 5’-ATCCTTGAAACAATTAC-3’
 Mut1: 5’-ATCCTTTCCCAAATTAC-3
(-1058 to -1041) WT2: 5’-AGACGGGGTTTCACCATG-3’
 Mut2: 5’-AGACGGTTGGGAACCATG-3’
Mut1
Luc
Luc
Mut2
**
(-212 to -196) WT1: 5’-GCGCTCCCGCTCCAGGCC-3’
 Mut1: 5’-GCGCTCAATAGCCAGGCC-3’
 (-95 to -77) WT2: 5’-AACTTTCCGTTCAGCTCA-3’
 Mut2: 5’-AACTTTAATGGCAGCTCA-3’
Relative luciferase activity (PFKL)
0
2
4
6
8
10
12
14
16
18
20
pGL4
Luc
-1486
Luc
WT
EV
OVOL2
**
Relative luciferase activity (PKM2)
Luc
Mut1
**
Mut2
Luc
0
10
20
30
40
50
60
**
pGL4
-776
Luc
Luc
Mut1/2
EV
OVOL2
Luc
WT
**
Luc
Mut1
(-907 to -889) WT1: 5’-CATGGTGAAACCCCGTCT-3’
 Mut2: 5’-CATGGTTCCCAACCGTCT-3’
(-579 to -560) WT2: 5’-CATTGCGAAACCCTGTCT-3’
 Mut2: 5’-CATTGCTCCCAACTGTCT-3’
(-312 to -295) WT3: 5’-TCCCTGAGCGGCAGGCGT-3’
 Mut3: 5’-TCCCTGCTATTCAGGCGT-3’
Luc
Mut2
**
(-883 to -866) WT1: 5’-GAGTTTGAAACTGTAACT-3’
 Mut1: 5’-GAGTTTTCCCATGTAACT-3
(-712 to -695) WT2: 5’-GCGACTCCGTTTCAAAAA-3’
 Mut2: 5’-GCGACTAATGGTCAAAAA-3’
Relative luciferase activity (ALDOA)
Relative luciferase activity (LDHA)
0
1
2
3
4
5
6
7
pGL4
Luc
-1050
0
10
40
20
30
50
60
70
pGL4
Luc
-1048
Luc
WT
**
EV
OVOL2
 WT
Luc
Mut1
Luc
**
**
EV
OVOL2
Mut1
Luc
Mut2
Luc
**
*
Mut2
Luc
Luc
Mut3
**
*
Luc
Mut1/2
Mut1/2
Luc
B
7
**
IgG
6
**
**
**
**
5
OVOL2
**
4
**
**
% Input
**
**
3
**
2
**
**
**
**
1
0
GPI
PFKL
HK2
LDHA
PKM2
ENO1
PGK1
ALDOA
PGAM1
GLUT1
GPI
HK2-1
HK2-2
PFKL-1
PFKL-2
ENO1-1
ENO1-2
PKM2-1
PKM2-2
LDHA-1
PGK1-1
PGK1-2
LDHA-2
PGK1-4
PGAM1-1
PGAM1-2
PGK1-3
ALDOA-2
ALDOA-3
ALDOA-1
GLUT1-3
GLUT1-1/2
Upstream
Promoters of glycolytic genes

## Slide 3
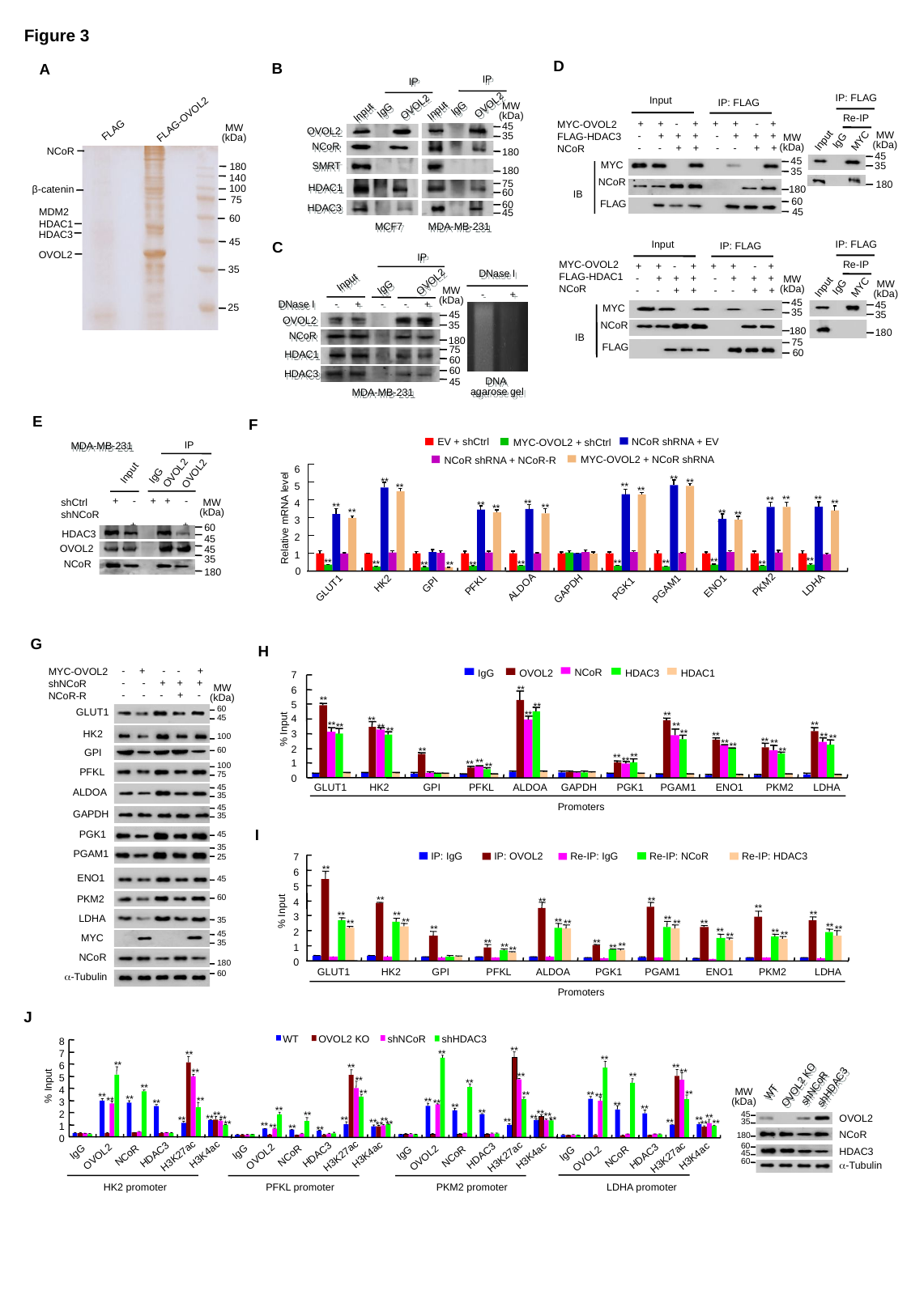

Figure 3
D
B
A
IP
IP
IP: FLAG
Input
IP: FLAG
 OVOL2
 OVOL2
 IgG
Input
 IgG
Input
MW
(kDa)
Re-IP
FLAG-OVOL2
 + + - + + + - +
 - + + + - + + +
 - - + + - - + +
MYC-OVOL2
FLAG-HDAC3
NCoR
45
FLAG
OVOL2
MW
(kDa)
35
Input
IgG
MYC
MW
(kDa)
MW
(kDa)
NCoR
180
45
NCoR
45
SMRT
35
180
180
35
MYC
140
75
180
HDAC1
100
180
NCoR
60
IB
β-catenin
75
60
60
HDAC3
MDM2
HDAC1
45
FLAG
45
60
MCF7 MDA-MB-231
HDAC3
45
C
Input
IP: FLAG
IP: FLAG
IP
OVOL2
Re-IP
MYC-OVOL2
FLAG-HDAC1
NCoR
 + + - + + + - +
 - + + + - + + +
 - - + + - - + +
35
DNase I
OVOL2
Input
Input
IgG
MYC
MW
(kDa)
IgG
MW
(kDa)
- +
MW
(kDa)
45
DNase I - + - - +
45
25
35
35
45
MYC
OVOL2
35
180
180
NCoR
NCoR
IB
180
75
75
60
HDAC1
FLAG
60
60
HDAC3
45
DNA
agarose gel
MDA-MB-231
E
F
IP
MDA-MB-231
NCoR shRNA + EV
EV + shCtrl
MYC-OVOL2 + shCtrl
Input
MYC-OVOL2 + NCoR shRNA
NCoR shRNA + NCoR-R
OVOL2
OVOL2
6
IgG
**
**
**
**
**
**
5
**
**
**
+ - + + -
- + - - +
shCtrl
shNCoR
**
**
**
**
**
**
4
MW
(kDa)
**
**
**
**
Relative mRNA level
3
60
HDAC3
45
2
OVOL2
45
35
**
1
**
**
**
**
**
**
**
NCoR
**
**
**
**
180
0
LDHA
HK2
GPI
PFKL
PKM2
ENO1
ALDOA
GLUT1
PGK1
PGAM1
GAPDH
G
H
- + - - +
- - + + +
- - - + -
MYC-OVOL2
shNCoR
NCoR-R
NCoR
HDAC3
HDAC1
IgG
OVOL2
7
**
MW
(kDa)
6
**
**
60
5
GLUT1
**
**
45
**
**
4
**
**
**
**
**
**
HK2
% Input
**
**
**
100
3
**
**
**
**
**
**
60
GPI
2
**
**
**
**
**
**
100
1
PFKL
75
0
45
ALDOA
GLUT1 HK2 GPI PFKL ALDOA GAPDH PGK1 PGAM1 ENO1 PKM2 LDHA
35
45
Promoters
GAPDH
35
I
PGK1
45
35
PGAM1
25
IP: IgG
Re-IP: NCoR
IP: OVOL2
Re-IP: IgG
Re-IP: HDAC3
7
**
6
ENO1
45
5
PKM2
60
**
**
**
**
4
**
**
**
% Input
LDHA
**
**
**
35
3
**
**
**
**
**
**
**
**
**
**
45
**
MYC
2
**
**
35
**
**
**
**
1
NCoR
180
0
60
α-Tubulin
GLUT1 HK2 GPI PFKL ALDOA PGK1 PGAM1 ENO1 PKM2 LDHA
Promoters
J
WT
OVOL2 KO
shNCoR
shHDAC3
8
**
**
**
**
7
**
**
**
6
**
**
**
**
**
**
5
OVOL2 KO
shHDAC3
**
shNCoR
WT
% Input
**
**
**
**
4
**
**
**
**
**
MW
(kDa)
**
**
**
**
**
3
**
**
**
**
**
**
**
45
**
**
**
OVOL2
**
**
**
**
**
2
**
**
**
**
**
**
**
35
**
**
**
**
**
**
1
NCoR
180
0
60
IgG
IgG
NCoR
NCoR
HDAC3
IgG
NCoR
IgG
NCoR
H3K4ac
H3K4ac
HDAC3
H3K4ac
HDAC3
H3K4ac
H3K27ac
H3K27ac
HDAC3
OVOL2
OVOL2
H3K27ac
HDAC3
45
H3K27ac
OVOL2
OVOL2
60
α-Tubulin
HK2 promoter PFKL promoter PKM2 promoter LDHA promoter

## Slide 4
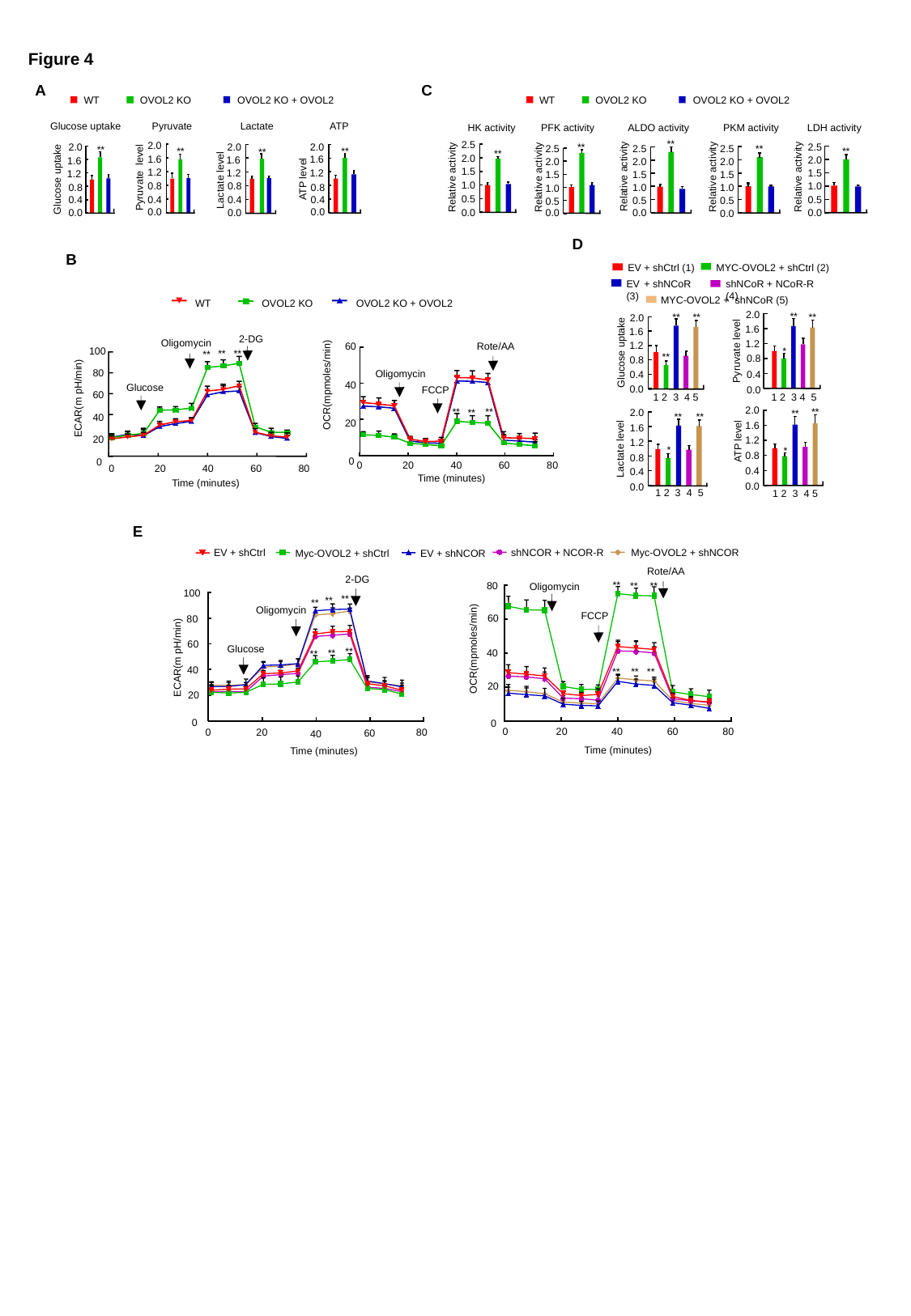

Figure 4
A
C
WT
WT
OVOL2 KO
OVOL2 KO + OVOL2
OVOL2 KO
OVOL2 KO + OVOL2
Glucose uptake Pyruvate Lactate ATP
HK activity PFK activity ALDO activity PKM activity LDH activity
**
 Glucose uptake
2.0
1.6
1.2
0.8
0.4
0.0
**
**
**
Pyruvate level
2.0
1.6
1.2
0.8
0.4
0.0
2.5
2.0
1.5
Relative activity
1.0
0.5
0.0
**
**
**
**
2.0
ATP level
1.6
1.2
0.8
0.4
0.0
2.0
 Lactate level
1.6
1.2
0.8
0.4
0.0
2.5
**
2.5
2.0
1.5
Relative activity
1.0
0.5
0.0
2.5
2.5
2.0
2.0
2.0
1.5
1.5
1.5
Relative activity
Relative activity
Relative activity
1.0
1.0
1.0
0.5
0.5
0.5
0.0
0.0
0.0
D
B
MYC-OVOL2 + shCtrl (2)
EV + shCtrl (1)
EV + shNCoR (3)
shNCoR + NCoR-R (4)
MYC-OVOL2 + shNCoR (5)
WT
OVOL2 KO + OVOL2
OVOL2 KO
**
 Glucose uptake
2.0
1.6
1.2
0.8
0.4
0.0
**
**
**
Pyruvate level
2.0
OCR(mpmoles/min)
1.6
2-DG
Oligomycin
1.2
*
Rote/AA
60
**
**
**
**
100
ECAR(m pH/min)
0.8
80
Oligomycin
0.4
40
Glucose
0.0
FCCP
60
1 2 3 4 5
1 2 3 4 5
**
**
**
**
**
2.0
**
**
2.0
ATP level
 Lactate level
40
20
1.6
1.6
20
1.2
1.2
*
*
0.8
0.8
0
0
0
20
40
60
80
0
20
40
60
80
0.4
0.4
Time (minutes)
Time (minutes)
0.0
0.0
1 2 3 4 5
1 2 3 4 5
E
Myc-OVOL2 + shNCOR
EV + shCtrl
shNCOR + NCOR-R
Myc-OVOL2 + shCtrl
EV + shNCOR
Rote/AA
**
OCR(mpmoles/min)
**
2-DG
**
80
Oligomycin
**
100
**
**
ECAR(m pH/min)
Oligomycin
FCCP
60
80
60
**
**
**
Glucose
40
**
**
**
40
20
20
0
0
0
20
40
60
80
0
20
80
60
40
Time (minutes)
Time (minutes)

## Slide 5
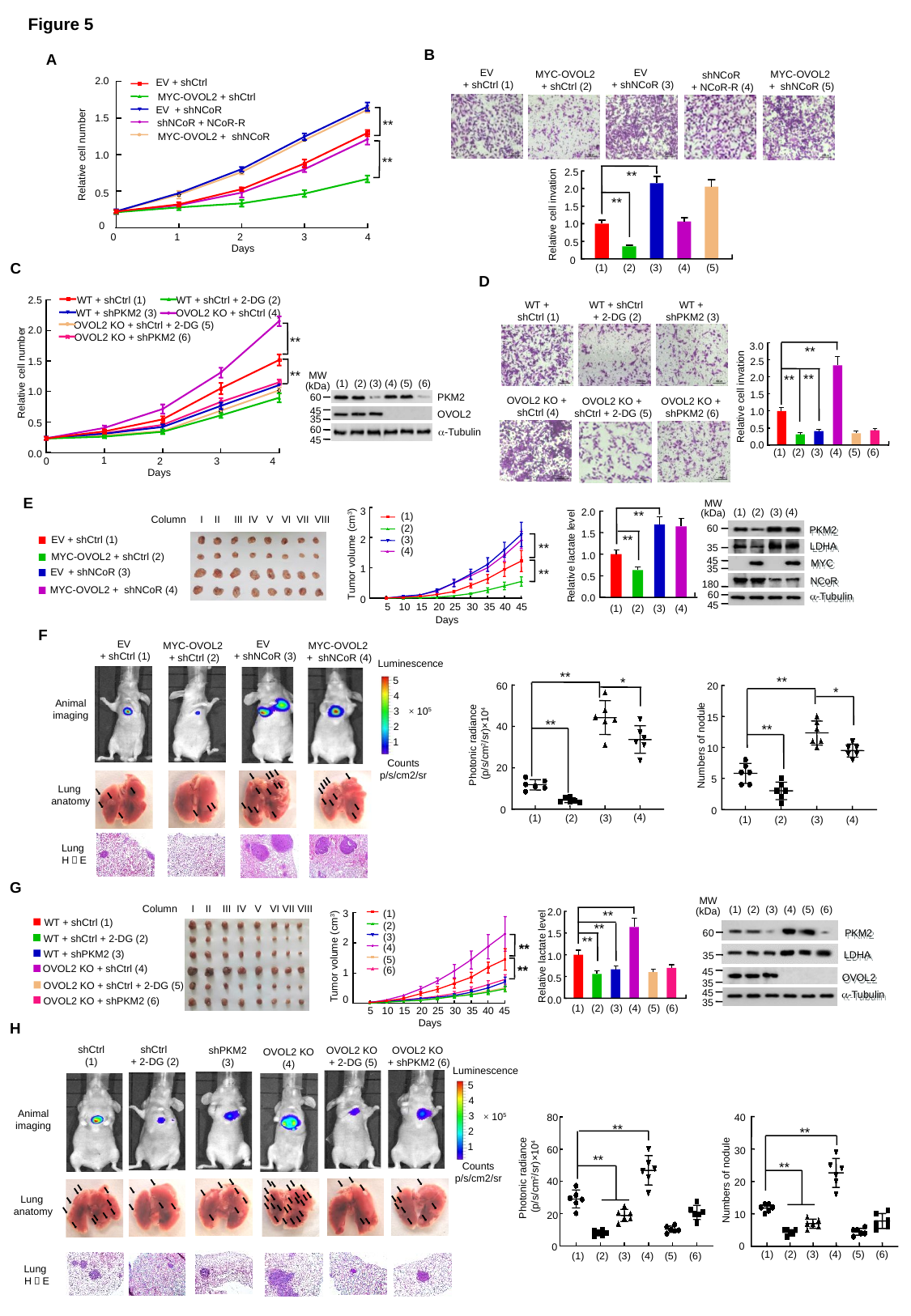

Figure 5
B
A
EV
+ shCtrl (1)
EV
 + shNCoR (3)
MYC-OVOL2
 + shCtrl (2)
MYC-OVOL2
 + shNCoR (5)
shNCoR
+ NCoR-R (4)
2.0
EV + shCtrl
MYC-OVOL2 + shCtrl
EV + shNCoR
**
1.5
shNCoR + NCoR-R
MYC-OVOL2 + shNCoR
Relative cell number
**
1.0
**
2.5
2.0
0.5
**
1.5
Relative cell invation
1.0
0
0
1
3
4
2
0.5
Days
C
0
 (1)
 (2)
 (3)
 (4)
 (5)
D
2.5
WT + shCtrl (1)
WT + shCtrl + 2-DG (2)
WT + shPKM2 (3)
OVOL2 KO + shCtrl (4)
OVOL2 KO + shCtrl + 2-DG (5)
OVOL2 KO + shPKM2 (6)
**
2.0
1.5
**
Relative cell number
1.0
0.5
0.0
1
0
2
3
4
Days
WT + shCtrl
 + 2-DG (2)
WT +
shPKM2 (3)
WT +
shCtrl (1)
**
3.0
2.5
**
**
2.0
MW
(kDa)
(1) (2) (3) (4) (5) (6)
Relative cell invation
60
1.5
PKM2
OVOL2 KO +
shCtrl (4)
OVOL2 KO +
shCtrl + 2-DG (5)
OVOL2 KO +
shPKM2 (6)
45
1.0
35
OVOL2
60
α-Tubulin
0.5
45
0.0
(1)
(2)
(3)
(4)
(5)
(6)
E
MW
(kDa)
**
3
2.0
(1) (2) (3) (4)
 (1)
 (2)
 (3)
 (4)
Column I II III IV V VI VII VIII
60
PKM2
**
1.5
EV + shCtrl (1)
LDHA
2
**
35
Tumor volume (cm3)
Relative lactate level
45
1.0
MYC-OVOL2 + shCtrl (2)
MYC
35
**
1
EV + shNCoR (3)
NCoR
0.5
180
60
MYC-OVOL2 + shNCoR (4)
α-Tubulin
0.0
45
0
5
10
15
20
25
30
35
40
45
(1)
(2)
(3)
(4)
Days
F
EV
+ shCtrl (1)
EV
 + shNCoR (3)
MYC-OVOL2
 + shCtrl (2)
MYC-OVOL2
 + shNCoR (4)
Luminescence
**
**
*
5
*
60
20
15
Numbers of nodule
10
5
0
(1)
(2)
(3)
(4)
4
Animal
imaging
3
× 105
**
**
2
40
Photonic radiance
(p/s/cm2/sr)×104
1
Counts
p/s/cm2/sr
20
Lung
 anatomy
0
(4)
(1)
(2)
(3)
Lung
 H＆E
G
MW
(kDa)
**
2.0
**
**
1.5
Relative lactate level
1.0
0.5
0.0
(1)
(2)
(3)
(4)
(5)
(6)
Column I II III IV V VI VII VIII
(1) (2) (3) (4) (5) (6)
3
(1)
(2)
(3)
(4)
(5)
(6)
WT + shCtrl (1)
PKM2
60
WT + shCtrl + 2-DG (2)
**
2
LDHA
35
Tumor volume (cm3)
WT + shPKM2 (3)
**
45
OVOL2 KO + shCtrl (4)
OVOL2
1
35
OVOL2 KO + shCtrl + 2-DG (5)
45
α-Tubulin
35
0
OVOL2 KO + shPKM2 (6)
5
10
15
20
25
30
35
40
45
H
Days
shCtrl
 + 2-DG (2)
shCtrl
(1)
shPKM2
(3)
OVOL2 KO
 + 2-DG (5)
OVOL2 KO
 + shPKM2 (6)
OVOL2 KO
(4)
Luminescence
5
4
Animal
imaging
3
× 105
80
40
**
**
2
1
60
30
**
**
Counts
p/s/cm2/sr
Photonic radiance
(p/s/cm2/sr)×104
Numbers of nodule
40
20
Lung
 anatomy
10
20
0
0
(1)
(2)
(3)
(4)
(5)
(6)
(1)
(2)
(3)
(4)
(5)
(6)
Lung
 H＆E

## Slide 6
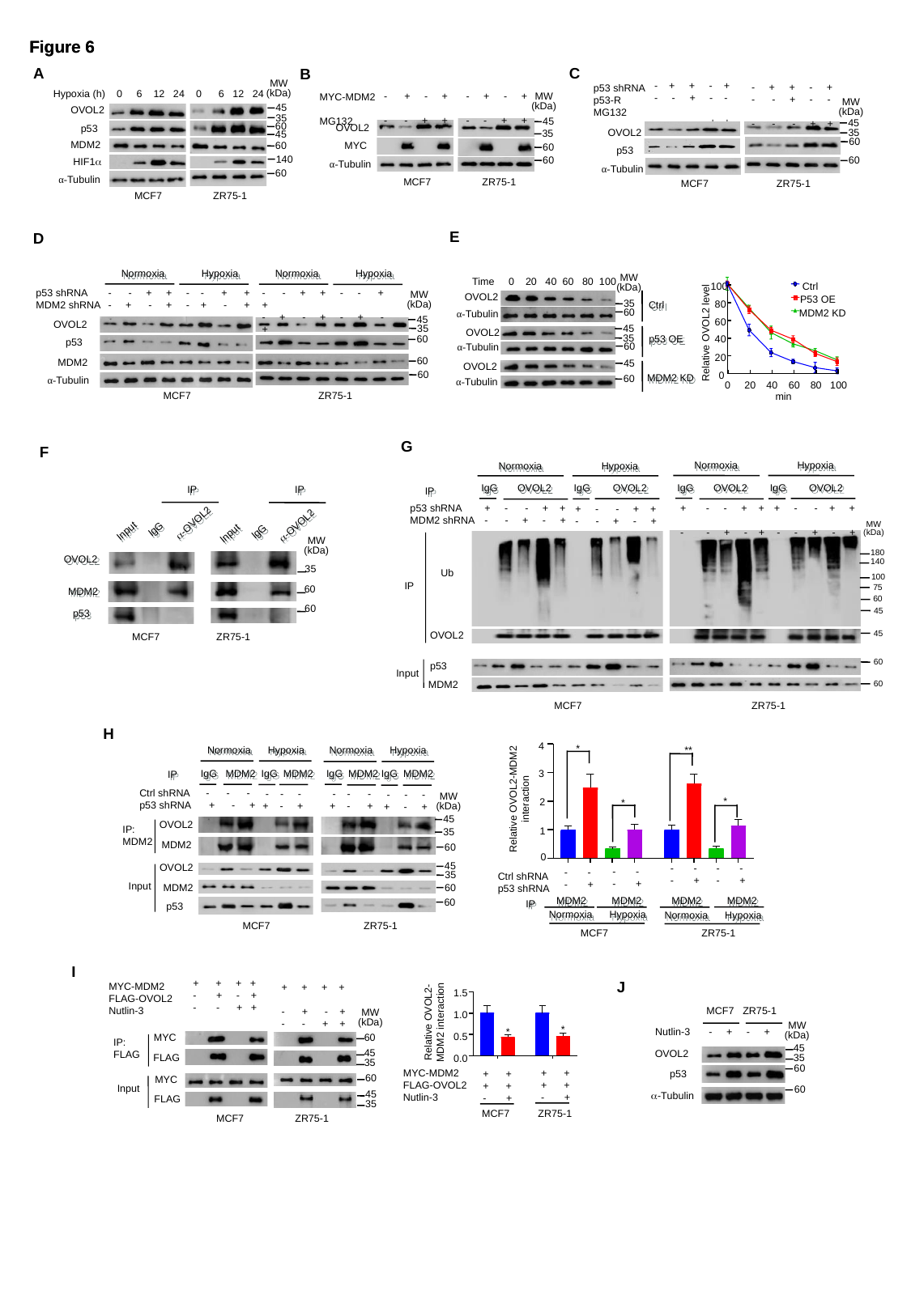

Figure 6
Figure 6
A
C
B
- + + - +
- - + - -
- - - + +
- + + - +
- - + - -
- - - + +
p53 shRNA
p53-R
MG132
MW
(kDa)
Hypoxia (h) 0 6 12 24 0 6 12 24
- + - +
- - + +
- + - +
- - + +
MYC-MDM2
MG132
MW
(kDa)
45
MW
(kDa)
OVOL2
35
45
45
60
OVOL2
p53
OVOL2
35
35
45
60
MDM2
60
MYC
60
p53
140
60
60
HIF1α
α-Tubulin
α-Tubulin
60
α-Tubulin
MCF7 ZR75-1
MCF7 ZR75-1
 MCF7 ZR75-1
E
D
Relative OVOL2 level
100
Ctrl
P53 OE
MDM2 KD
80
60
40
20
0
0
20
40
60
80
100
min
Normoxia Hypoxia
Normoxia Hypoxia
Time 0 20 40 60 80 100
MW
(kDa)
p53 shRNA
MDM2 shRNA
- - + + - - + +
- + - + - + - +
- - + + - - + +
- + - + - + - +
OVOL2
MW
(kDa)
35
Ctrl
60
α-Tubulin
45
OVOL2
35
45
OVOL2
35
60
p53 OE
p53
60
α-Tubulin
60
MDM2
45
OVOL2
60
MDM2 KD
60
α-Tubulin
α-Tubulin
MCF7 ZR75-1
G
F
Normoxia Hypoxia
Normoxia Hypoxia
IgG OVOL2 IgG OVOL2
IgG OVOL2 IgG OVOL2
IP
IP
 α-OVOL2
 α-OVOL2
Input
 IgG
Input
 IgG
MW
(kDa)
OVOL2
35
60
MDM2
60
p53
 MCF7 ZR75-1
IP
p53 shRNA
MDM2 shRNA
+ - - + +
- - + - +
+ - - + +
- - + - +
 + - - + +
 - - + - +
+ - - + +
- - + - +
MW
(kDa)
180
140
Ub
100
IP
75
60
45
45
OVOL2
60
p53
Input
MDM2
60
MCF7 ZR75-1
H
Relative OVOL2-MDM2 interaction
*
Normoxia Hypoxia
**
Normoxia Hypoxia
4
IgG MDM2 IgG MDM2
IgG MDM2 IgG MDM2
IP
3
Ctrl shRNA
p53 shRNA
 - - - + - +
 - - -
+ - +
 - - -
+ - +
 - - -
+ - +
*
*
MW
(kDa)
2
45
OVOL2
IP:
MDM2
35
1
MDM2
60
0
45
OVOL2
 - -
 - +
 - -
 - +
 - -
 - +
 - -
 - +
35
Ctrl shRNA
p53 shRNA
Input
60
MDM2
MDM2 MDM2
MDM2 MDM2
60
IP
p53
Normoxia Hypoxia
Normoxia Hypoxia
MCF7 ZR75-1
MCF7 ZR75-1
I
Relative OVOL2- MDM2 interaction
+ + + +
- + - +
- - + +
MYC-MDM2
FLAG-OVOL2
Nutlin-3
+ + + +
- + - +
- - + +
MW
(kDa)
60
MYC
IP:
FLAG
45
FLAG
35
60
MYC
Input
45
FLAG
35
MCF7 ZR75-1
J
1.5
MCF7 ZR75-1
Nutlin-3
- + - +
MW
(kDa)
45
OVOL2
35
60
p53
60
α-Tubulin
1.0
*
*
0.5
0.0
MYC-MDM2
FLAG-OVOL2
Nutlin-3
+ +
+ +
- +
+ +
+ +
- +
MCF7 ZR75-1

## Slide 7
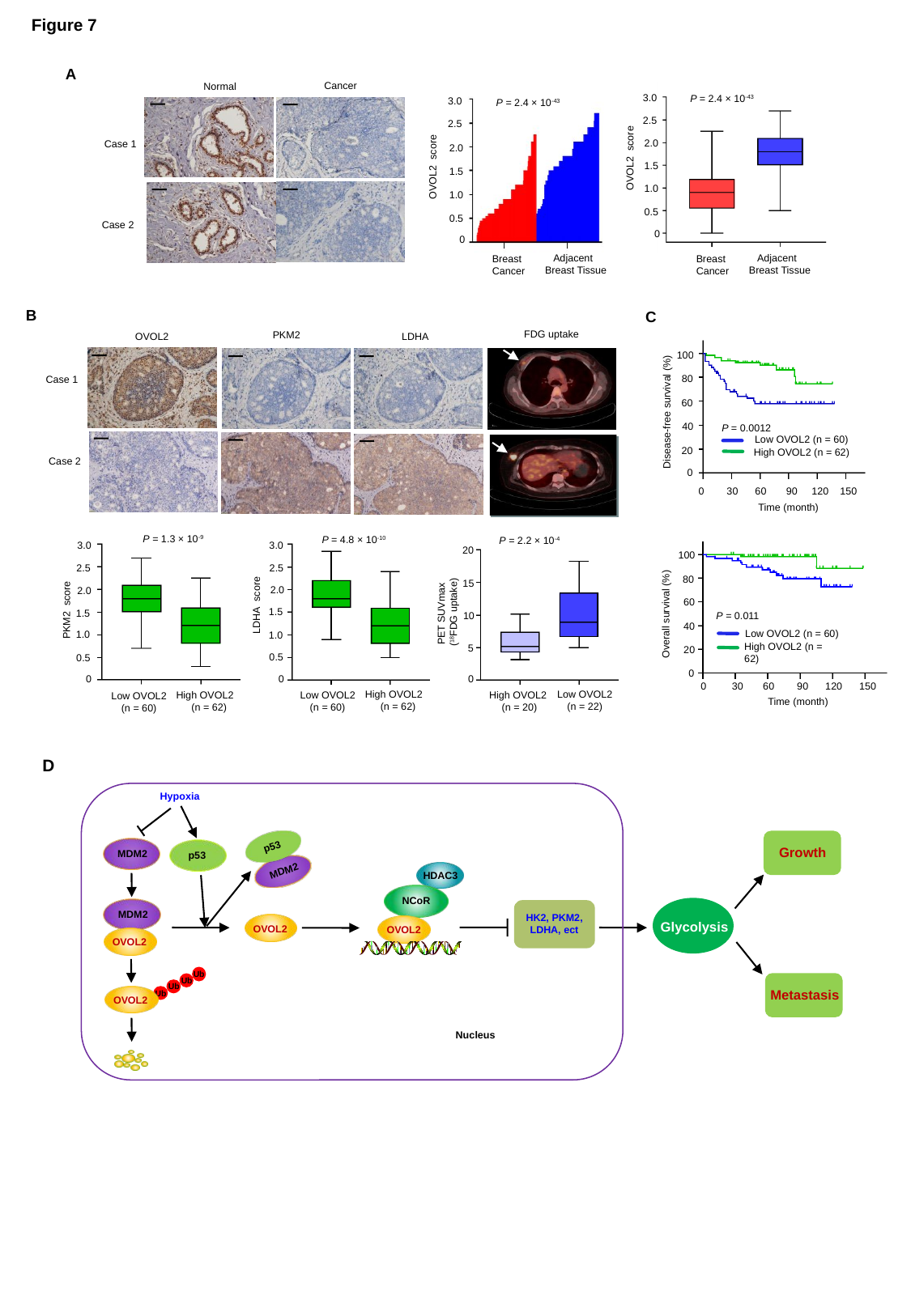

Figure 7
A
Cancer
Normal
3.0
P = 2.4 × 10^-43^
3.0
P = 2.4 × 10^-43^
2.5
2.5
OVOL2 score
2.0
Case 1
2.0
OVOL2 score
1.5
1.5
1.0
1.0
0.5
0.5
Case 2
 0
 0
Adjacent
Breast Tissue
Adjacent
Breast Tissue
Breast
Cancer
Breast
Cancer
B
C
FDG uptake
PKM2
LDHA
OVOL2
100
 80
Case 1
 60
Disease-free survival (%)
 40
P = 0.0012
Low OVOL2 (n = 60)
 20
High OVOL2 (n = 62)
Case 2
 0
0 30 60 90 120 150
Time (month)
P = 1.3 × 10^-9^
P = 4.8 × 10^-10^
P = 2.2 × 10^-4^
3.0
3.0
20
100
 80
2.5
2.5
15
LDHA score
2.0
2.0
PKM2 score
 60
PET SUVmax
(18FDG uptake)
Overall survival (%)
1.5
1.5
10
P = 0.011
 40
Low OVOL2 (n = 60)
1.0
1.0
High OVOL2 (n = 62)
5
 20
0.5
0.5
 0
0
 0
 0
0 30 60 90 120 150
Low OVOL2
(n = 22)
High OVOL2 (n = 62)
High OVOL2 (n = 62)
Low OVOL2
(n = 60)
High OVOL2
 (n = 20)
Low OVOL2
(n = 60)
Time (month)
D
Hypoxia
p53
MDM2
Growth
MDM2
p53
HDAC3
NCoR
HK2, PKM2,
LDHA, ect
MDM2
Glycolysis
OVOL2
OVOL2
OVOL2
Ub
Ub
Ub
Ub
Metastasis
OVOL2
Nucleus
