# Supplementary material for: Transcriptional Repression of Aerobic Glycolysis by OVOL2 in Breast Cancer
Source: Adv Sci (Weinh). 2022 Jul 27;9(27):2200705. doi: 10.1002/advs.202200705 (PMC9507357; doi:10.1002/advs.202200705)
Supplement: Supplementary file 3 — Supporting Information [file ADVS-9-2200705-s002.ppt]

## Slide 1
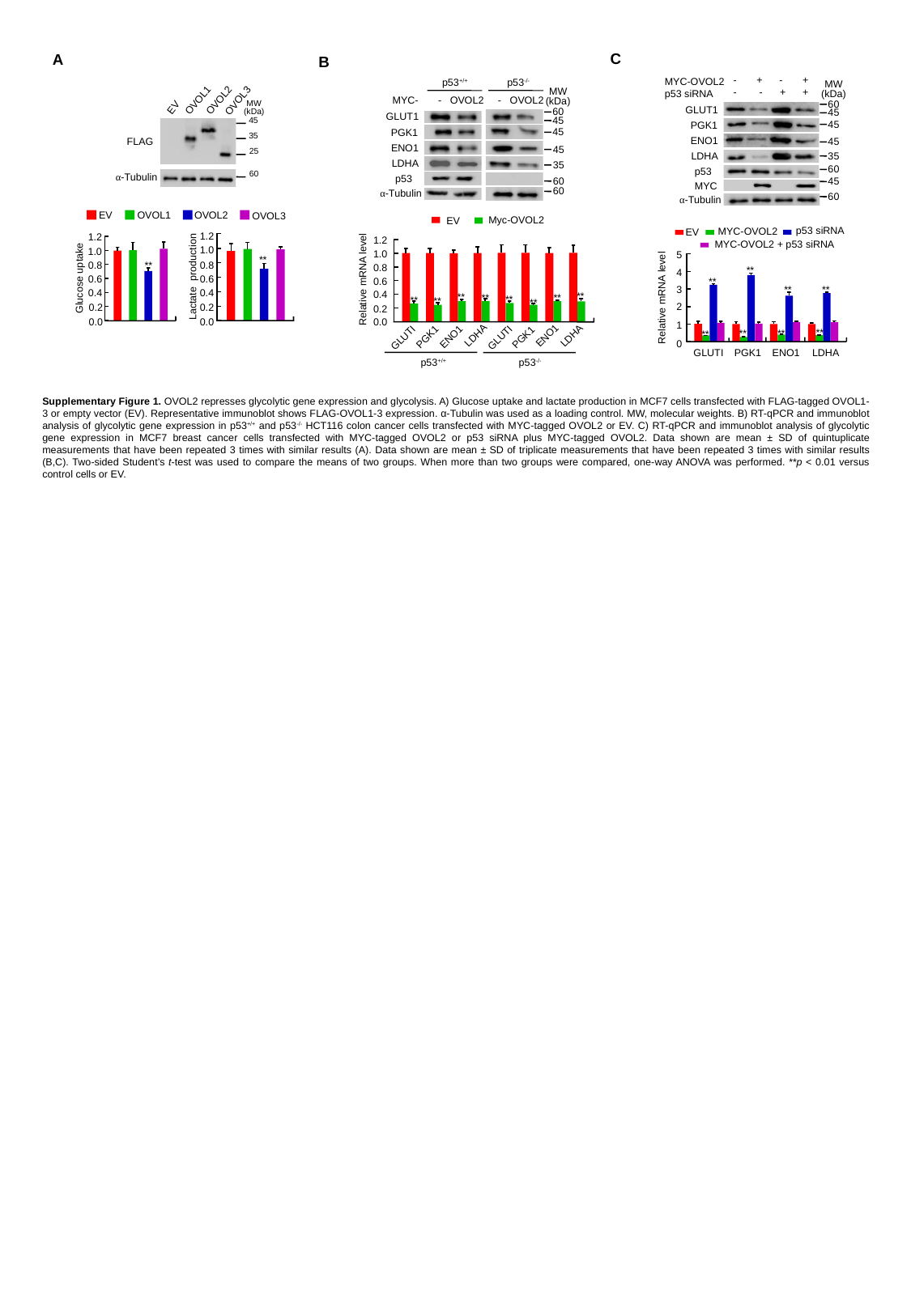

C
A
B
- + - +
- - + +
MYC-OVOL2
p53 siRNA
p53+/+
p53-/-
MW
(kDa)
OVOL1
OVOL2
OVOL3
EV
MYC- - OVOL2 - OVOL2
MW
(kDa)
60
MW
(kDa)
GLUT1
60
45
GLUT1
45
45
45
PGK1
45
PGK1
35
ENO1
FLAG
45
ENO1
45
25
35
LDHA
LDHA
35
60
p53
60
α-Tubulin
p53
60
45
MYC
60
α-Tubulin
60
α-Tubulin
EV
OVOL1
OVOL2
OVOL3
Myc-OVOL2
EV
Relative mRNA level
 Lactate production
p53 siRNA
MYC-OVOL2
EV
1.2
1.2
 Glucose uptake
1.2
MYC-OVOL2 + p53 siRNA
Relative mRNA level
1.0
1.0
**
1.0
5
**
**
0.8
0.8
0.8
4
**
0.6
0.6
0.6
**
**
3
**
**
**
**
0.4
**
0.4
**
0.4
**
**
2
0.2
0.2
0.2
0.0
0.0
0.0
1
**
**
**
**
LDHA
ENO1
LDHA
ENO1
PGK1
PGK1
GLUTI
GLUTI
0
GLUTI
PGK1
ENO1
LDHA
p53+/+
p53-/-
Supplementary Figure 1. OVOL2 represses glycolytic gene expression and glycolysis. A) Glucose uptake and lactate production in MCF7 cells transfected with FLAG-tagged OVOL1-3 or empty vector (EV). Representative immunoblot shows FLAG-OVOL1-3 expression. α-Tubulin was used as a loading control. MW, molecular weights. B) RT-qPCR and immunoblot analysis of glycolytic gene expression in p53+/+ and p53-/- HCT116 colon cancer cells transfected with MYC-tagged OVOL2 or EV. C) RT-qPCR and immunoblot analysis of glycolytic gene expression in MCF7 breast cancer cells transfected with MYC-tagged OVOL2 or p53 siRNA plus MYC-tagged OVOL2. Data shown are mean ± SD of quintuplicate measurements that have been repeated 3 times with similar results (A). Data shown are mean ± SD of triplicate measurements that have been repeated 3 times with similar results (B,C). Two-sided Student’s t-test was used to compare the means of two groups. When more than two groups were compared, one-way ANOVA was performed. **p < 0.01 versus control cells or EV.

## Slide 2
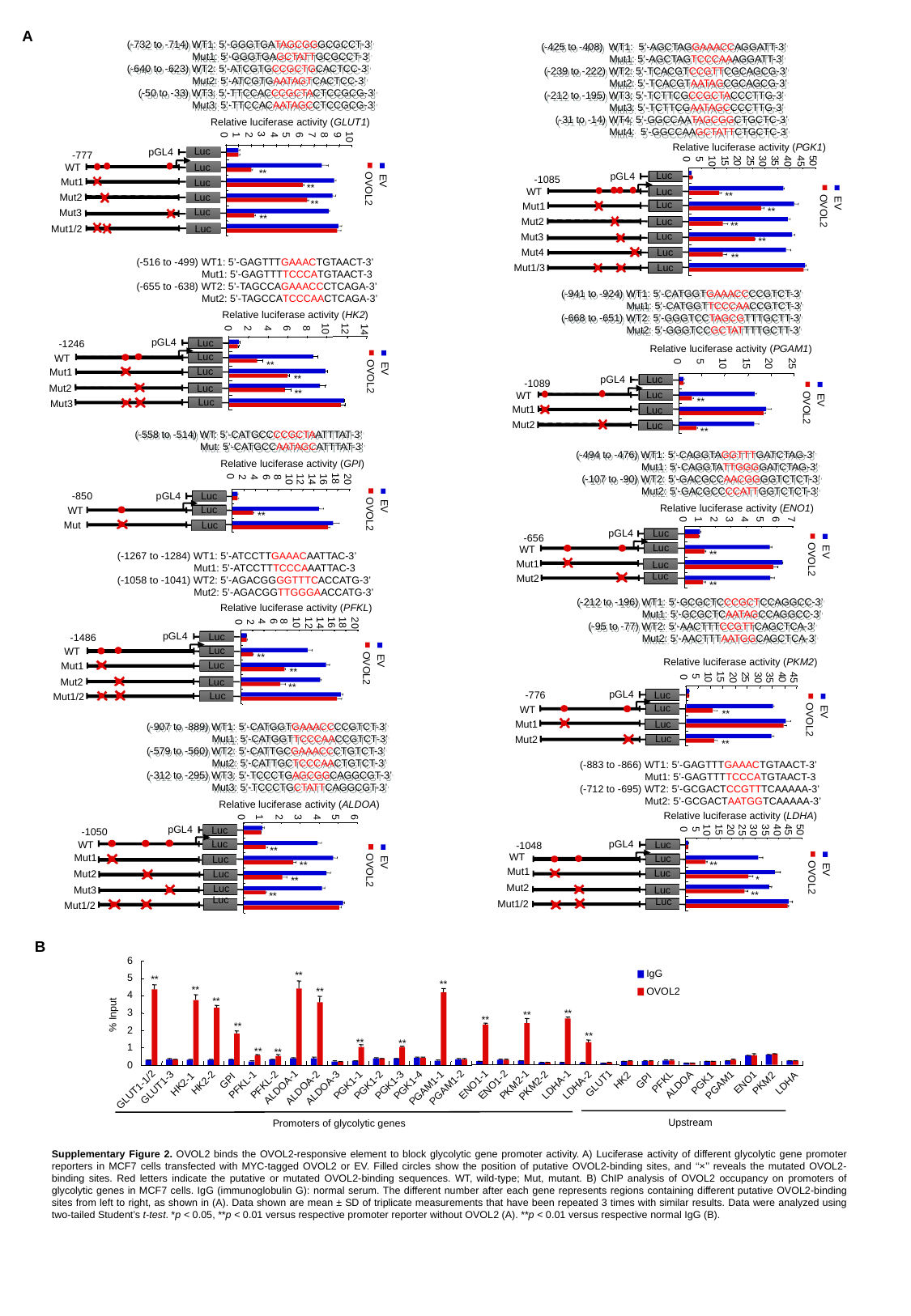

A
(-732 to -714) WT1: 5’-GGGTGATAGCGGGCGCCT-3’
 Mut1: 5’-GGGTGAGCTATTGCGCCT-3’
(-640 to -623) WT2: 5’-ATCGTGCCGCTGCACTCC-3’
 Mut2: 5’-ATCGTGAATAGTCACTCC-3’
 (-50 to -33) WT3: 5’-TTCCACCCGCTACTCCGCG-3’
 Mut3: 5’-TTCCACAATAGCCTCCGCG-3’
 (-425 to -408) WT1: 5’-AGCTAGGAAACCAGGATT-3’
 Mut1: 5’-AGCTAGTCCCAAAGGATT-3’
 (-239 to -222) WT2: 5’-TCACGTCCGTTCGCAGCG-3’
 Mut2: 5’-TCACGTAATAGCGCAGCG-3’
 (-212 to -195) WT3: 5’-TCTTCGCCGCTACCCTTG-3’
 Mut3: 5’-TCTTCGAATAGCCCCTTG-3’
 (-31 to -14) WT4: 5’-GGCCAATAGCGGCTGCTC-3’
 Mut4: 5’-GGCCAAGCTATTCTGCTC-3’
Relative luciferase activity (GLUT1)
3
0
2
6
7
8
9
1
4
5
10
Relative luciferase activity (PGK1)
Luc
pGL4
-777
WT
Luc
0
5
10
15
20
25
30
35
40
45
50
EV
OVOL2
**
Luc
pGL4
-1085
Mut1
Luc
**
WT
Luc
EV
OVOL2
**
Mut2
Luc
**
Luc
Mut1
**
Luc
Mut3
**
Mut2
Luc
**
Mut1/2
Luc
Luc
Mut3
**
Mut4
Luc
**
(-516 to -499) WT1: 5’-GAGTTTGAAACTGTAACT-3’
 Mut1: 5’-GAGTTTTCCCATGTAACT-3
(-655 to -638) WT2: 5’-TAGCCAGAAACCCTCAGA-3’
 Mut2: 5’-TAGCCATCCCAACTCAGA-3’
Luc
Mut1/3
 (-941 to -924) WT1: 5’-CATGGTGAAACCCCGTCT-3’
 Mut1: 5’-CATGGTTCCCAACCGTCT-3’
 (-668 to -651) WT2: 5’-GGGTCCTAGCGTTTGCTT-3’
 Mut2: 5’-GGGTCCGCTATTTTGCTT-3’
Relative luciferase activity (HK2)
0
4
2
6
8
10
12
14
pGL4
Luc
-1246
Relative luciferase activity (PGAM1)
Luc
WT
EV
OVOL2
**
5
0
10
15
20
25
Luc
Mut1
**
Luc
pGL4
-1089
Mut2
Luc
EV
OVOL2
**
Luc
WT
**
Luc
 Mut3
Mut1
Luc
Mut2
Luc
**
(-558 to -514) WT: 5’-CATGCCCCGCTAATTTAT-3’
 Mut: 5’-CATGCCAATAGCATTTAT-3’
(-494 to -476) WT1: 5’-CAGGTAGGTTTGATCTAG-3’
 Mut1: 5’-CAGGTATTGGGGATCTAG-3’
 (-107 to -90) WT2: 5’-GACGCCAACGGGGTCTCT-3’
 Mut2: 5’-GACGCCCCATTGGTCTCT-3’
Relative luciferase activity (GPI)
0
2
4
6
8
18
20
10
12
16
14
pGL4
-850
Luc
EV
OVOL2
Relative luciferase activity (ENO1)
Luc
WT
**
Mut
Luc
0
1
2
4
5
6
7
3
pGL4
Luc
-656
EV
OVOL2
Luc
WT
**
(-1267 to -1284) WT1: 5’-ATCCTTGAAACAATTAC-3’
 Mut1: 5’-ATCCTTTCCCAAATTAC-3
(-1058 to -1041) WT2: 5’-AGACGGGGTTTCACCATG-3’
 Mut2: 5’-AGACGGTTGGGAACCATG-3’
Mut1
Luc
Luc
Mut2
**
(-212 to -196) WT1: 5’-GCGCTCCCGCTCCAGGCC-3’
 Mut1: 5’-GCGCTCAATAGCCAGGCC-3’
 (-95 to -77) WT2: 5’-AACTTTCCGTTCAGCTCA-3’
 Mut2: 5’-AACTTTAATGGCAGCTCA-3’
Relative luciferase activity (PFKL)
4
6
8
2
0
18
16
12
10
14
20
pGL4
Luc
-1486
Luc
WT
EV
OVOL2
**
Relative luciferase activity (PKM2)
Luc
Mut1
**
Mut2
Luc
5
15
0
10
20
25
30
35
40
45
**
pGL4
-776
Luc
Luc
Mut1/2
EV
OVOL2
Luc
WT
**
Luc
Mut1
(-907 to -889) WT1: 5’-CATGGTGAAACCCCGTCT-3’
 Mut1: 5’-CATGGTTCCCAACCGTCT-3’
(-579 to -560) WT2: 5’-CATTGCGAAACCCTGTCT-3’
 Mut2: 5’-CATTGCTCCCAACTGTCT-3’
(-312 to -295) WT3: 5’-TCCCTGAGCGGCAGGCGT-3’
 Mut3: 5’-TCCCTGCTATTCAGGCGT-3’
Luc
Mut2
**
(-883 to -866) WT1: 5’-GAGTTTGAAACTGTAACT-3’
 Mut1: 5’-GAGTTTTCCCATGTAACT-3
(-712 to -695) WT2: 5’-GCGACTCCGTTTCAAAAA-3’
 Mut2: 5’-GCGACTAATGGTCAAAAA-3’
Relative luciferase activity (ALDOA)
Relative luciferase activity (LDHA)
3
0
1
2
4
5
6
pGL4
Luc
-1050
0
5
10
15
20
25
30
35
40
45
50
Luc
pGL4
Luc
WT
-1048
**
EV
OVOL2
WT
Mut1
Luc
Luc
EV
OVOL2
**
**
Mut1
Luc
Mut2
Luc
*
**
Mut2
Luc
Mut3
Luc
**
**
Luc
Luc
Mut1/2
Mut1/2
B
6
5
4
3
2
1
0
**
**
IgG
**
**
**
OVOL2
**
**
**
**
% Input
**
**
**
**
**
**
HK2
GPI
GPI
PFKL
ENO1
HK2-2
LDHA
GLUT1
PGK1
PKM2
HK2-1
ALDOA
PFKL-1
ENO1-1
LDHA-1
PGAM1
PGK1-3
PKM2-1
LDHA-2
PFKL-2
ENO1-2
PGK1-1
PGK1-2
PGK1-4
PKM2-2
ALDOA-1
GLUT1-3
ALDOA-2
PGAM1-2
ALDOA-3
PGAM1-1
GLUT1-1/2
Upstream
Promoters of glycolytic genes
Supplementary Figure 2. OVOL2 binds the OVOL2-responsive element to block glycolytic gene promoter activity. A) Luciferase activity of different glycolytic gene promoter reporters in MCF7 cells transfected with MYC-tagged OVOL2 or EV. Filled circles show the position of putative OVOL2-binding sites, and ‘‘×’’ reveals the mutated OVOL2-binding sites. Red letters indicate the putative or mutated OVOL2-binding sequences. WT, wild-type; Mut, mutant. B) ChIP analysis of OVOL2 occupancy on promoters of glycolytic genes in MCF7 cells. IgG (immunoglobulin G): normal serum. The different number after each gene represents regions containing different putative OVOL2-binding sites from left to right, as shown in (A). Data shown are mean ± SD of triplicate measurements that have been repeated 3 times with similar results. Data were analyzed using two-tailed Student’s t-test. *p < 0.05, **p < 0.01 versus respective promoter reporter without OVOL2 (A). **p < 0.01 versus respective normal IgG (B).

## Slide 3
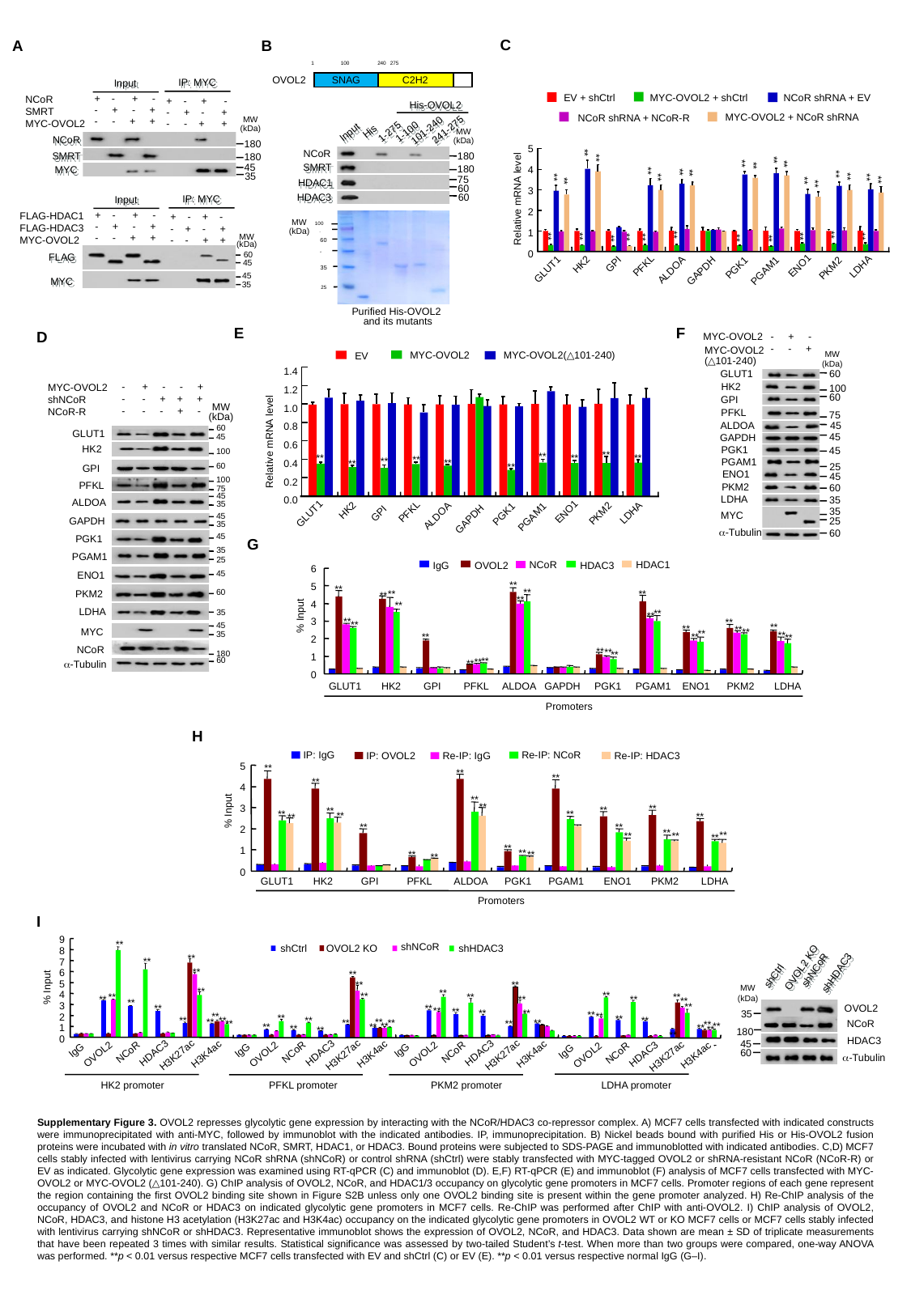

C
A
B
1 100 240 275
OVOL2
IP: MYC
Input
SNAG
C2H2
NCoR
SMRT
MYC-OVOL2
+ - + -
- + - +
- - + +
+ - + -
- + - +
- - + +
MYC-OVOL2 + shCtrl
EV + shCtrl
NCoR shRNA + EV
MYC-OVOL2 + NCoR shRNA
NCoR shRNA + NCoR-R
His-OVOL2
MW
(kDa)
241-275
101-240
His
1-100
1-275
Input
MW
(kDa)
NCoR
180
NCoR
Relative mRNA level
5
SMRT
180
180
**
**
**
45
SMRT
**
**
180
MYC
**
**
35
4
**
**
**
75
**
**
**
**
HDAC1
**
**
**
60
**
3
HDAC3
60
IP: MYC
Input
+ - + -
- + - +
- - + +
FLAG-HDAC1
FLAG-HDAC3
MYC-OVOL2
+ - + -
- + - +
- - + +
2
MW
(kDa)
100
75
MW
(kDa)
1
**
**
**
**
**
**
**
60
**
**
**
**
60
FLAG
45
0
HK2
GPI
45
LDHA
PFKL
ENO1
PGK1
PKM2
GLUT1
ALDOA
35
PGAM1
GAPDH
45
MYC
35
25
Purified His-OVOL2
 and its mutants
F
E
D
- + -
- - +
MYC-OVOL2
MW
(kDa)
MYC-OVOL2
(△101-240)
MYC-OVOL2
MYC-OVOL2(△101-240)
EV
GLUT1
60
1.4
HK2
- + - - +
- - + + +
- - - + -
MYC-OVOL2
shNCoR
NCoR-R
100
Relative mRNA level
1.2
60
GPI
PFKL
1.0
MW
(kDa)
75
ALDOA
45
60
0.8
GLUT1
45
GAPDH
45
HK2
PGK1
45
0.6
100
**
**
**
**
**
**
**
PGAM1
**
**
25
**
60
GPI
0.4
ENO1
45
100
PFKL
PKM2
60
0.2
75
45
LDHA
35
ALDOA
35
0.0
LDHA
HK2
35
ENO1
PFKL
GPI
PKM2
GLUT1
PGK1
MYC
ALDOA
PGAM1
45
GAPDH
GAPDH
25
35
α-Tubulin
60
45
PGK1
G
35
PGAM1
25
NCoR
HDAC1
HDAC3
IgG
OVOL2
6
ENO1
45
**
**
5
**
PKM2
60
**
**
**
**
**
4
LDHA
**
35
**
**
% Input
**
**
**
45
3
**
**
**
MYC
**
**
35
**
**
**
2
NCoR
**
**
**
180
**
**
60
1
**
α-Tubulin
0
GLUT1 HK2 GPI PFKL ALDOA GAPDH PGK1 PGAM1 ENO1 PKM2 LDHA
Promoters
H
IP: IgG
Re-IP: NCoR
IP: OVOL2
Re-IP: IgG
Re-IP: HDAC3
**
5
**
**
**
4
**
**
**
**
**
**
**
3
**
**
**
% Input
**
**
**
**
2
**
**
**
**
**
**
**
1
**
0
GLUT1 HK2 GPI PFKL ALDOA PGK1 PGAM1 ENO1 PKM2 LDHA
Promoters
I
**
9
shNCoR
shCtrl
OVOL2 KO
shHDAC3
8
**
**
OVOL2 KO
7
shNCoR
**
shHDAC3
**
shCtrl
6
**
**
MW
(kDa)
5
**
**
% Input
**
**
**
**
**
**
**
**
4
**
**
**
OVOL2
**
**
**
**
3
**
**
35
**
**
**
**
**
**
**
**
**
**
**
2
**
**
**
**
**
NCoR
**
**
**
**
**
**
**
**
**
180
1
**
HDAC3
45
0
NCoR
NCoR
NCoR
NCoR
IgG
IgG
IgG
IgG
HDAC3
HDAC3
HDAC3
H3K4ac
H3K4ac
H3K4ac
OVOL2
OVOL2
OVOL2
60
HDAC3
H3K4ac
OVOL2
H3K27ac
H3K27ac
H3K27ac
H3K27ac
α-Tubulin
HK2 promoter PFKL promoter PKM2 promoter LDHA promoter
Supplementary Figure 3. OVOL2 represses glycolytic gene expression by interacting with the NCoR/HDAC3 co-repressor complex. A) MCF7 cells transfected with indicated constructs were immunoprecipitated with anti-MYC, followed by immunoblot with the indicated antibodies. IP, immunoprecipitation. B) Nickel beads bound with purified His or His-OVOL2 fusion proteins were incubated with in vitro translated NCoR, SMRT, HDAC1, or HDAC3. Bound proteins were subjected to SDS-PAGE and immunoblotted with indicated antibodies. C,D) MCF7 cells stably infected with lentivirus carrying NCoR shRNA (shNCoR) or control shRNA (shCtrl) were stably transfected with MYC-tagged OVOL2 or shRNA-resistant NCoR (NCoR-R) or EV as indicated. Glycolytic gene expression was examined using RT-qPCR (C) and immunoblot (D). E,F) RT-qPCR (E) and immunoblot (F) analysis of MCF7 cells transfected with MYC-OVOL2 or MYC-OVOL2 (△101-240). G) ChIP analysis of OVOL2, NCoR, and HDAC1/3 occupancy on glycolytic gene promoters in MCF7 cells. Promoter regions of each gene represent the region containing the first OVOL2 binding site shown in Figure S2B unless only one OVOL2 binding site is present within the gene promoter analyzed. H) Re-ChIP analysis of the occupancy of OVOL2 and NCoR or HDAC3 on indicated glycolytic gene promoters in MCF7 cells. Re-ChIP was performed after ChIP with anti-OVOL2. I) ChIP analysis of OVOL2, NCoR, HDAC3, and histone H3 acetylation (H3K27ac and H3K4ac) occupancy on the indicated glycolytic gene promoters in OVOL2 WT or KO MCF7 cells or MCF7 cells stably infected with lentivirus carrying shNCoR or shHDAC3. Representative immunoblot shows the expression of OVOL2, NCoR, and HDAC3. Data shown are mean ± SD of triplicate measurements that have been repeated 3 times with similar results. Statistical significance was assessed by two-tailed Student’s t-test. When more than two groups were compared, one-way ANOVA was performed. **p < 0.01 versus respective MCF7 cells transfected with EV and shCtrl (C) or EV (E). **p < 0.01 versus respective normal IgG (G–I).

## Slide 4
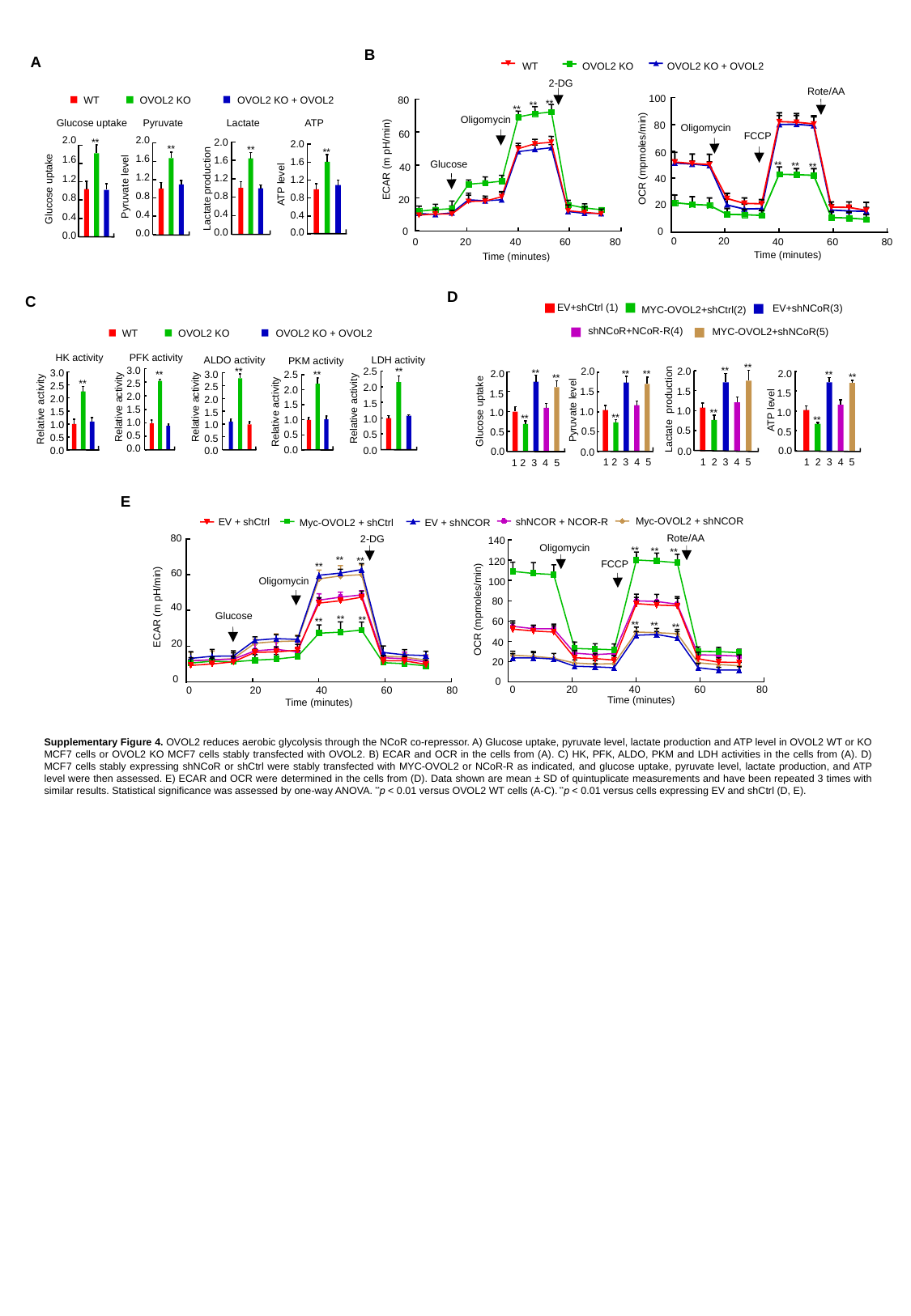

B
A
WT
OVOL2 KO + OVOL2
OVOL2 KO
2-DG
Rote/AA
**
**
100
WT
OVOL2 KO
OVOL2 KO + OVOL2
80
**
ECAR (m pH/min)
OCR (mpmoles/min)
Oligomycin
Glucose uptake
2.0
1.6
1.2
0.8
0.4
0.0
Lactate
Lactate production
2.0
1.6
1.2
0.8
0.4
0.0
ATP
Pyruvate
2.0
Pyruvate level
1.6
1.2
0.8
0.4
0.0
80
Oligomycin
60
**
FCCP
**
**
2.0
**
 Glucose uptake
60
ATP level
**
**
**
1.6
Glucose
40
40
1.2
0.8
20
20
0.4
0
0
0.0
0
20
40
60
80
0
20
40
60
80
Time (minutes)
Time (minutes)
D
C
EV+shCtrl (1)
EV+shNCoR(3)
MYC-OVOL2+shCtrl(2)
shNCoR+NCoR-R(4)
MYC-OVOL2+shNCoR(5)
WT
OVOL2 KO
OVOL2 KO + OVOL2
ATP level
PFK activity
3.0
2.5
2.0
Relative activity
1.5
1.0
0.5
0.0
HK activity
3.0
2.5
2.0
Relative activity
1.5
1.0
0.5
0.0
ALDO activity
3.0
2.5
2.0
Relative activity
1.5
1.0
0.5
0.0
LDH activity
2.5
2.0
1.5
Relative activity
1.0
0.5
0.0
PKM activity
2.5
2.0
1.5
Relative activity
1.0
0.5
0.0
**
Pyruvate level
**
Lactate production
**
**
**
**
**
**
**
**
 Glucose uptake
**
2.0
**
2.0
2.0
2.0
**
1.5
1.5
1.5
1.5
**
1.0
**
1.0
**
1.0
1.0
**
0.5
0.5
0.5
0.5
0.0
0.0
0.0
0.0
1 2 3 4 5
1 2 3 4 5
1 2 3 4 5
1 2 3 4 5
E
Myc-OVOL2 + shNCOR
EV + shCtrl
shNCOR + NCOR-R
Myc-OVOL2 + shCtrl
EV + shNCOR
80
Rote/AA
2-DG
140
**
**
**
Oligomycin
**
**
OCR (mpmoles/min)
**
120
FCCP
ECAR (m pH/min)
60
Oligomycin
100
80
40
**
**
Glucose
**
**
**
**
60
40
20
20
0
0
0
20
40
80
60
0
20
40
60
80
Time (minutes)
Time (minutes)
Supplementary Figure 4. OVOL2 reduces aerobic glycolysis through the NCoR co-repressor. A) Glucose uptake, pyruvate level, lactate production and ATP level in OVOL2 WT or KO MCF7 cells or OVOL2 KO MCF7 cells stably transfected with OVOL2. B) ECAR and OCR in the cells from (A). C) HK, PFK, ALDO, PKM and LDH activities in the cells from (A). D) MCF7 cells stably expressing shNCoR or shCtrl were stably transfected with MYC-OVOL2 or NCoR-R as indicated, and glucose uptake, pyruvate level, lactate production, and ATP level were then assessed. E) ECAR and OCR were determined in the cells from (D). Data shown are mean ± SD of quintuplicate measurements and have been repeated 3 times with similar results. Statistical significance was assessed by one-way ANOVA. **p < 0.01 versus OVOL2 WT cells (A-C). **p < 0.01 versus cells expressing EV and shCtrl (D, E).

## Slide 5
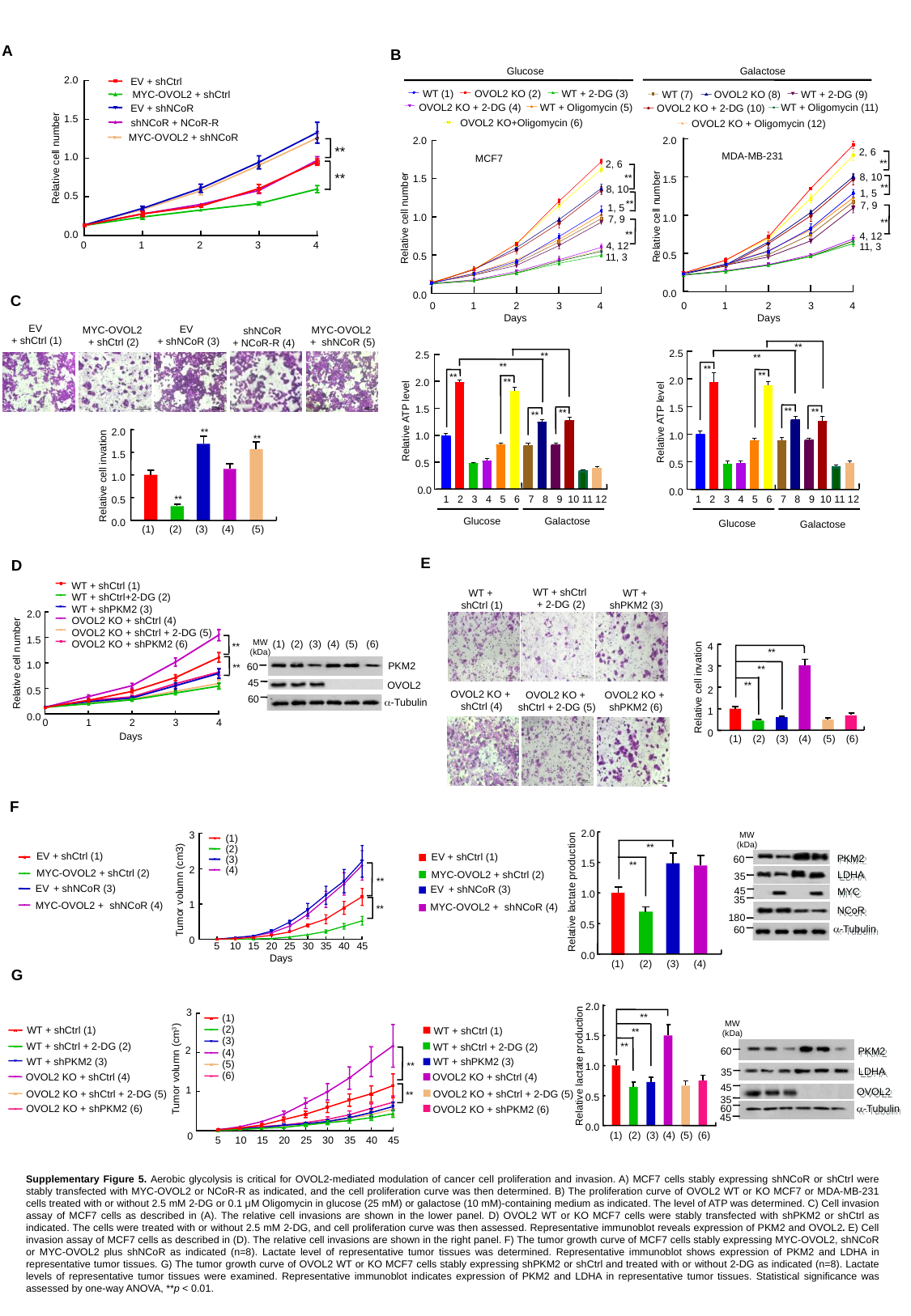

A
B
Glucose
Galactose
2.0
EV + shCtrl
WT (1)
WT + 2-DG (3)
OVOL2 KO (2)
WT (7)
WT + 2-DG (9)
MYC-OVOL2 + shCtrl
OVOL2 KO (8)
WT + Oligomycin (5)
OVOL2 KO + 2-DG (4)
WT + Oligomycin (11)
EV + shNCoR
OVOL2 KO + 2-DG (10)
1.5
shNCoR + NCoR-R
OVOL2 KO+Oligomycin (6)
OVOL2 KO + Oligomycin (12)
MYC-OVOL2 + shNCoR
2.0
2.0
**
2, 6
Relative cell number
MDA-MB-231
**
1.0
MCF7
2, 6
**
8, 10
**
1.5
1.5
**
8, 10
1, 5
0.5
**
7, 9
1, 5
Relative cell number
Relative cell number
7, 9
**
1.0
1.0
**
4, 12
0.0
4, 12
11, 3
0
1
2
3
4
11, 3
0.5
0.5
C
0.0
0.0
0
0
1
2
3
4
1
2
3
4
Days
Days
EV
+ shCtrl (1)
EV
 + shNCoR (3)
MYC-OVOL2
 + shNCoR (5)
MYC-OVOL2
 + shCtrl (2)
shNCoR
+ NCoR-R (4)
**
**
**
2.5
2.5
**
**
**
**
**
2.0
2.0
**
**
**
Relative ATP level
1.5
Relative ATP level
**
1.5
**
2.0
**
1.0
1.0
1.5
0.5
0.5
Relative cell invation
1.0
0.0
0.0
**
1 2 3 4 5 6 7 8 9 10 11 12
1 2 3 4 5 6 7 8 9 10 11 12
0.5
Glucose
Galactose
Glucose
Galactose
0.0
 (1)
 (2)
 (3)
(4)
 (5)
E
D
WT + shCtrl (1)
WT + shCtrl+2-DG (2)
WT + shPKM2 (3)
2.0
OVOL2 KO + shCtrl (4)
OVOL2 KO + shCtrl + 2-DG (5)
1.5
**
OVOL2 KO + shPKM2 (6)
Relative cell number
**
1.0
0.5
0.0
0
1
2
3
4
Days
WT + shCtrl
 + 2-DG (2)
WT +
shPKM2 (3)
WT +
shCtrl (1)
MW
(kDa)
(1) (2) (3) (4) (5) (6)
**
4
60
**
PKM2
3
45
**
OVOL2
Relative cell invation
2
60
OVOL2 KO +
shCtrl (4)
OVOL2 KO +
shCtrl + 2-DG (5)
OVOL2 KO +
shPKM2 (6)
α-Tubulin
1
0
(1)
(2)
(3)
(4)
(5)
(6)
F
MW
(kDa)
2.0
3
(1)
(2)
(3)
(4)
**
PKM2
60
EV + shCtrl (1)
EV + shCtrl (1)
MYC-OVOL2 + shCtrl (2)
EV + shNCoR (3)
MYC-OVOL2 + shNCoR (4)
**
1.5
LDHA
**
2
35
MYC-OVOL2 + shCtrl (2)
45
MYC
Tumor volumn (cm3)
Relative lactate production
EV + shNCoR (3)
35
1.0
**
NCoR
1
MYC-OVOL2 + shNCoR (4)
180
α-Tubulin
60
0.5
0
5
10
15
20
25
30
35
40
45
0.0
Days
(1)
(2)
(3)
(4)
G
2.0
**
**
1.5
**
Relative lactate production
1.0
0.5
0.0
(1)
(2)
(3)
(4)
(5)
(6)
3
2
Tumor volumn (cm3)
1
0
5
10
15
20
25
30
35
40
45
(1)
(2)
(3)
(4)
(5)
(6)
MW
(kDa)
WT + shCtrl (1)
WT + shCtrl (1)
WT + shCtrl + 2-DG (2)
WT + shPKM2 (3)
OVOL2 KO + shCtrl (4)
OVOL2 KO + shCtrl + 2-DG (5)
OVOL2 KO + shPKM2 (6)
60
PKM2
WT + shCtrl + 2-DG (2)
**
WT + shPKM2 (3)
LDHA
35
OVOL2 KO + shCtrl (4)
45
OVOL2
**
35
OVOL2 KO + shCtrl + 2-DG (5)
α-Tubulin
60
OVOL2 KO + shPKM2 (6)
45
Supplementary Figure 5. Aerobic glycolysis is critical for OVOL2-mediated modulation of cancer cell proliferation and invasion. A) MCF7 cells stably expressing shNCoR or shCtrl were stably transfected with MYC-OVOL2 or NCoR-R as indicated, and the cell proliferation curve was then determined. B) The proliferation curve of OVOL2 WT or KO MCF7 or MDA-MB-231 cells treated with or without 2.5 mM 2-DG or 0.1 μM Oligomycin in glucose (25 mM) or galactose (10 mM)-containing medium as indicated. The level of ATP was determined. C) Cell invasion assay of MCF7 cells as described in (A). The relative cell invasions are shown in the lower panel. D) OVOL2 WT or KO MCF7 cells were stably transfected with shPKM2 or shCtrl as indicated. The cells were treated with or without 2.5 mM 2-DG, and cell proliferation curve was then assessed. Representative immunoblot reveals expression of PKM2 and OVOL2. E) Cell invasion assay of MCF7 cells as described in (D). The relative cell invasions are shown in the right panel. F) The tumor growth curve of MCF7 cells stably expressing MYC-OVOL2, shNCoR or MYC-OVOL2 plus shNCoR as indicated (n=8). Lactate level of representative tumor tissues was determined. Representative immunoblot shows expression of PKM2 and LDHA in representative tumor tissues. G) The tumor growth curve of OVOL2 WT or KO MCF7 cells stably expressing shPKM2 or shCtrl and treated with or without 2-DG as indicated (n=8). Lactate levels of representative tumor tissues were examined. Representative immunoblot indicates expression of PKM2 and LDHA in representative tumor tissues. Statistical significance was assessed by one-way ANOVA, **p < 0.01.

## Slide 6
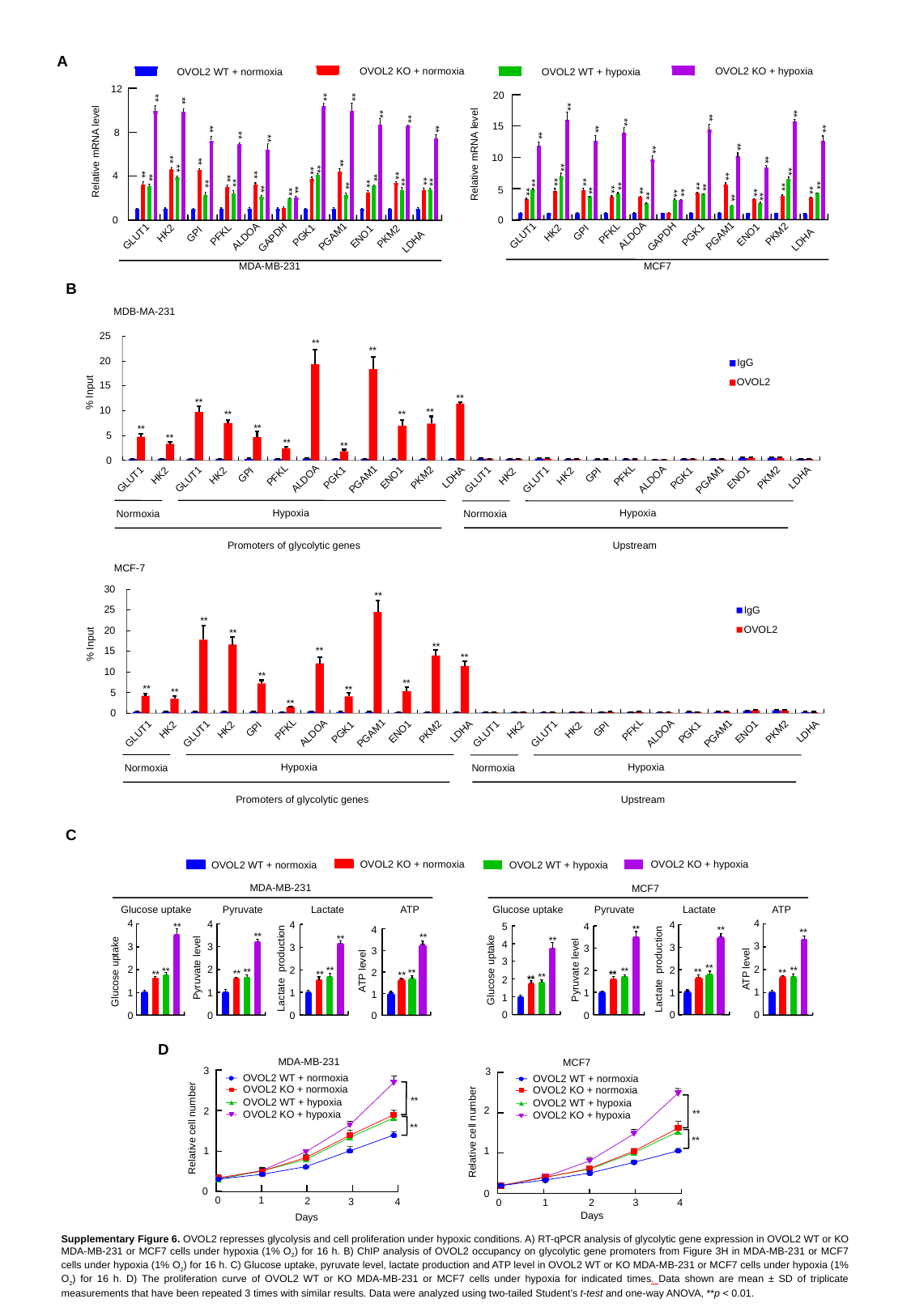

A
OVOL2 KO + normoxia
OVOL2 KO + hypoxia
Relative mRNA level
OVOL2 WT + hypoxia
OVOL2 WT + normoxia
12
**
**
**
Relative mRNA level
**
20
**
**
**
**
**
**
**
**
**
**
15
**
**
8
**
**
**
**
**
**
**
10
**
**
**
**
**
**
**
**
**
**
**
**
4
**
**
**
**
**
**
**
**
**
**
**
**
**
**
**
**
**
**
**
**
**
**
**
**
**
**
5
**
**
**
0
0
LDHA
LDHA
GPI
HK2
HK2
PFKL
PKM2
GPI
ENO1
PFKL
PGK1
PGK1
GLUT1
ALDOA
PKM2
PGAM1
ALDOA
PGAM1
GLUT1
ENO1
GAPDH
GAPDH
MDA-MB-231
MCF7
B
MDB-MA-231
25
20
15
10
5
0
IgG
OVOL2
**
**
**
% Input
**
**
**
**
**
**
**
**
**
GPI
PFKL
HK2
HK2
LDHA
PKM2
ENO1
PGK1
ALDOA
PGAM1
GLUT1
GLUT1
GPI
PFKL
HK2
HK2
LDHA
PKM2
ENO1
PGK1
ALDOA
PGAM1
GLUT1
GLUT1
Hypoxia
Hypoxia
Normoxia
Normoxia
Upstream
Promoters of glycolytic genes
MCF-7
30
25
20
15
10
5
0
IgG
OVOL2
**
**
**
**
% Input
**
**
**
**
**
**
**
**
GPI
PFKL
HK2
HK2
LDHA
PKM2
ENO1
PGK1
ALDOA
PGAM1
GLUT1
GLUT1
GPI
PFKL
HK2
HK2
LDHA
PKM2
ENO1
PGK1
ALDOA
PGAM1
GLUT1
GLUT1
Hypoxia
Hypoxia
Normoxia
Normoxia
Upstream
Promoters of glycolytic genes
C
OVOL2 KO + normoxia
OVOL2 KO + hypoxia
OVOL2 WT + hypoxia
OVOL2 WT + normoxia
MDA-MB-231
MCF7
Lactate production
Glucose uptake Pyruvate Lactate ATP
Glucose uptake Pyruvate Lactate ATP
Lactate production
ATP level
ATP level
**
**
4
4
**
4
4
4
Pyruvate level
5
**
4
 Glucose uptake
4
 Glucose uptake
**
Pyruvate level
**
**
**
4
3
3
3
3
3
3
3
3
**
**
**
**
**
**
**
**
**
**
**
**
**
**
**
2
2
2
2
2
**
2
2
**
**
2
1
1
1
1
1
1
1
1
0
0
0
0
0
0
0
0
D
MDA-MB-231
MCF7
3
3
OVOL2 WT + normoxia
OVOL2 WT + normoxia
**
OVOL2 KO + normoxia
OVOL2 KO + normoxia
**
OVOL2 WT + hypoxia
OVOL2 WT + hypoxia
2
2
OVOL2 KO + hypoxia
OVOL2 KO + hypoxia
**
Relative cell number
Relative cell number
**
1
1
0
0
0
1
2
4
3
0
1
2
3
4
Days
Days
Supplementary Figure 6. OVOL2 represses glycolysis and cell proliferation under hypoxic conditions. A) RT-qPCR analysis of glycolytic gene expression in OVOL2 WT or KO MDA-MB-231 or MCF7 cells under hypoxia (1% O2) for 16 h. B) ChIP analysis of OVOL2 occupancy on glycolytic gene promoters from Figure 3H in MDA-MB-231 or MCF7 cells under hypoxia (1% O2) for 16 h. C) Glucose uptake, pyruvate level, lactate production and ATP level in OVOL2 WT or KO MDA-MB-231 or MCF7 cells under hypoxia (1% O2) for 16 h. D) The proliferation curve of OVOL2 WT or KO MDA-MB-231 or MCF7 cells under hypoxia for indicated times. Data shown are mean ± SD of triplicate measurements that have been repeated 3 times with similar results. Data were analyzed using two-tailed Student’s t-test and one-way ANOVA, **p < 0.01.

## Slide 7
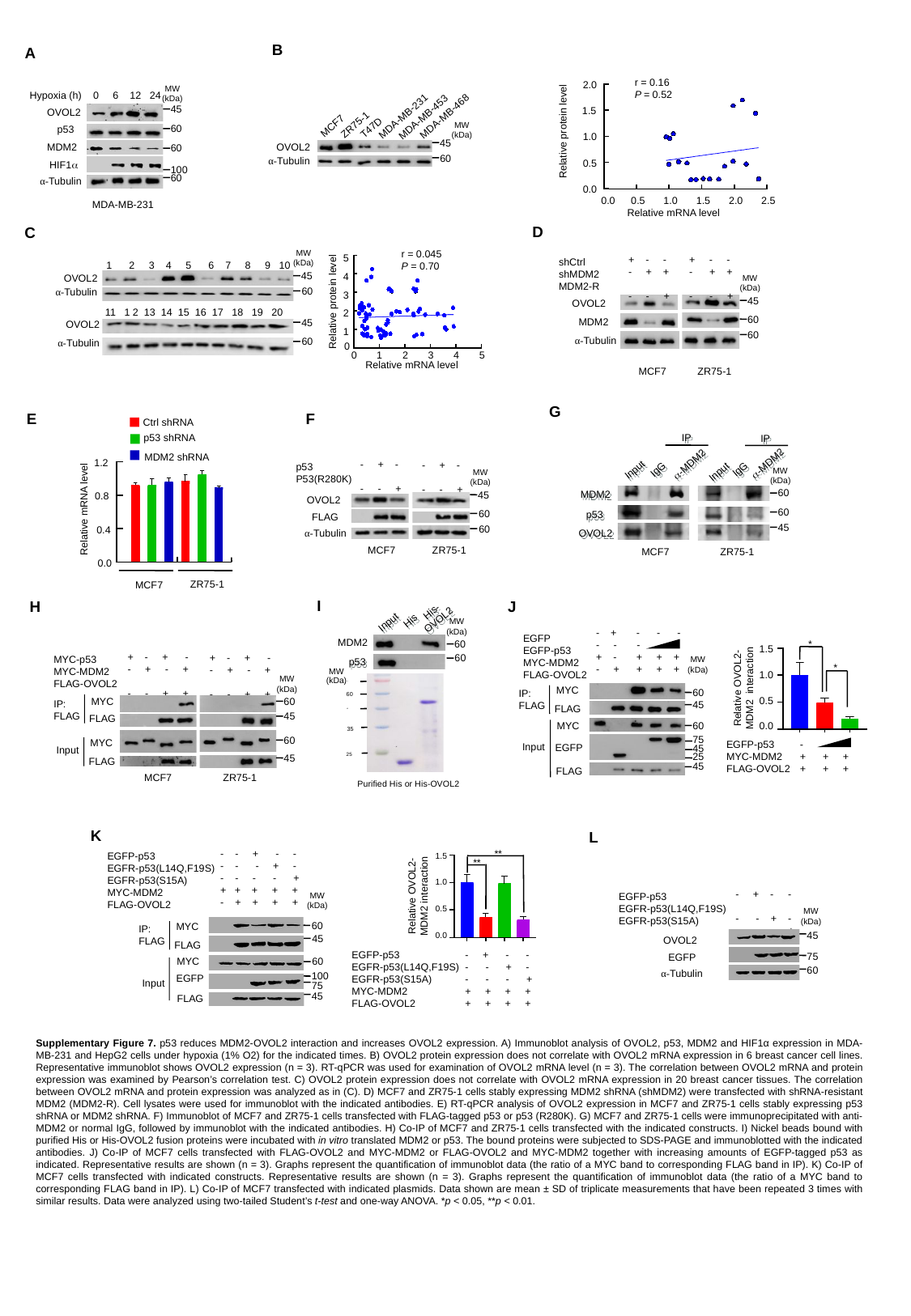

Relative protein level
r = 0.16
P = 0.52
2.0
1.5
Relative mRNA level
1.0
0.5
0.0
0.0
0.5
1.0
1.5
2.0
2.5
B
A
MDA-MB-231
MDA-MB-468
MDA-MB-453
MCF7
ZR75-1
T47D
OVOL2
α-Tubulin
MW
(kDa)
Hypoxia (h) 0 6 12 24
45
OVOL2
MW
(kDa)
60
p53
45
MDM2
60
60
HIF1α
100
60
α-Tubulin
MDA-MB-231
D
C
Relative protein level
r = 0.045
P = 0.70
5
4
3
2
1
0
0
1
2
3
4
5
MW
(kDa)
+ - -
- + +
- - +
+ - -
- + +
- - +
shCtrl
shMDM2
MDM2-R
1 2 3 4 5 6 7 8 9 10
45
OVOL2
MW
(kDa)
60
α-Tubulin
45
OVOL2
11 1 2 13 14 15 16 17 18 19 20
60
Relative mRNA level
MDM2
45
OVOL2
60
α-Tubulin
60
α-Tubulin
MCF7 ZR75-1
G
E
F
Ctrl shRNA
p53 shRNA
Relative mRNA level
MDM2 shRNA
1.2
0.8
0.4
0.0
ZR75-1
MCF7
IP
IP
 α-MDM2
 α-MDM2
Input
 IgG
 IgG
Input
MDM2
p53
OVOL2
MCF7 ZR75-1
- + -
- - +
- + -
- - +
p53
P53(R280K)
MW
(kDa)
MW
(kDa)
60
45
OVOL2
60
60
FLAG
45
60
α-Tubulin
MCF7 ZR75-1
I
J
H
His-
OVOL2
His
Input
MW
(kDa)
Relative OVOL2-
MDM2 interaction
- + - - -
- - -
+ - + + +
- + + + +
EGFP
EGFP-p53
MYC-MDM2
FLAG-OVOL2
MDM2
60
*
1.5
+ - + -
- + - +
- - + +
+ - + -
- + - +
- - + +
60
MYC-p53
MYC-MDM2
FLAG-OVOL2
MW
(kDa)
p53
*
MW
(kDa)
MW
(kDa)
1.0
75
MYC
60
IP:
FLAG
60
60
MYC
IP:
FLAG
45
0.5
FLAG
45
45
FLAG
MYC
60
35
EGFP-p53
MYC-MDM2
FLAG-OVOL2
0.0
75
60
MYC
-
+ + +
+ + +
Input
EGFP
45
Input
25
25
45
FLAG
45
FLAG
MCF7 ZR75-1
Purified His or His-OVOL2
K
L
Relative OVOL2-
MDM2 interaction
- - + - -
- - - + -
- - - - +
+ + + + +
- + + + +
EGFP-p53
EGFR-p53(L14Q,F19S)
EGFR-p53(S15A)
MYC-MDM2
FLAG-OVOL2
MYC
IP:
FLAG
FLAG
MYC
EGFP
Input
FLAG
**
**
1.5
1.0
0.5
0.0
- + - -
- - + -
- - - +
EGFP-p53
EGFR-p53(L14Q,F19S)
EGFR-p53(S15A)
OVOL2
EGFP
α-Tubulin
MW
(kDa)
MW
(kDa)
60
45
45
- + - -
- - + -
- - - +
+ + + +
+ + + +
EGFP-p53
EGFR-p53(L14Q,F19S)
EGFR-p53(S15A)
MYC-MDM2
FLAG-OVOL2
75
60
60
100
75
45
Supplementary Figure 7. p53 reduces MDM2-OVOL2 interaction and increases OVOL2 expression. A) Immunoblot analysis of OVOL2, p53, MDM2 and HIF1α expression in MDA-MB-231 and HepG2 cells under hypoxia (1% O2) for the indicated times. B) OVOL2 protein expression does not correlate with OVOL2 mRNA expression in 6 breast cancer cell lines. Representative immunoblot shows OVOL2 expression (n = 3). RT-qPCR was used for examination of OVOL2 mRNA level (n = 3). The correlation between OVOL2 mRNA and protein expression was examined by Pearson’s correlation test. C) OVOL2 protein expression does not correlate with OVOL2 mRNA expression in 20 breast cancer tissues. The correlation between OVOL2 mRNA and protein expression was analyzed as in (C). D) MCF7 and ZR75-1 cells stably expressing MDM2 shRNA (shMDM2) were transfected with shRNA-resistant MDM2 (MDM2-R). Cell lysates were used for immunoblot with the indicated antibodies. E) RT-qPCR analysis of OVOL2 expression in MCF7 and ZR75-1 cells stably expressing p53 shRNA or MDM2 shRNA. F) Immunoblot of MCF7 and ZR75-1 cells transfected with FLAG-tagged p53 or p53 (R280K). G) MCF7 and ZR75-1 cells were immunoprecipitated with anti-MDM2 or normal IgG, followed by immunoblot with the indicated antibodies. H) Co-IP of MCF7 and ZR75-1 cells transfected with the indicated constructs. I) Nickel beads bound with purified His or His-OVOL2 fusion proteins were incubated with in vitro translated MDM2 or p53. The bound proteins were subjected to SDS-PAGE and immunoblotted with the indicated antibodies. J) Co-IP of MCF7 cells transfected with FLAG-OVOL2 and MYC-MDM2 or FLAG-OVOL2 and MYC-MDM2 together with increasing amounts of EGFP-tagged p53 as indicated. Representative results are shown (n = 3). Graphs represent the quantification of immunoblot data (the ratio of a MYC band to corresponding FLAG band in IP). K) Co-IP of MCF7 cells transfected with indicated constructs. Representative results are shown (n = 3). Graphs represent the quantification of immunoblot data (the ratio of a MYC band to corresponding FLAG band in IP). L) Co-IP of MCF7 transfected with indicated plasmids. Data shown are mean ± SD of triplicate measurements that have been repeated 3 times with similar results. Data were analyzed using two-tailed Student’s t-test and one-way ANOVA. *p < 0.05, **p < 0.01.

## Slide 8
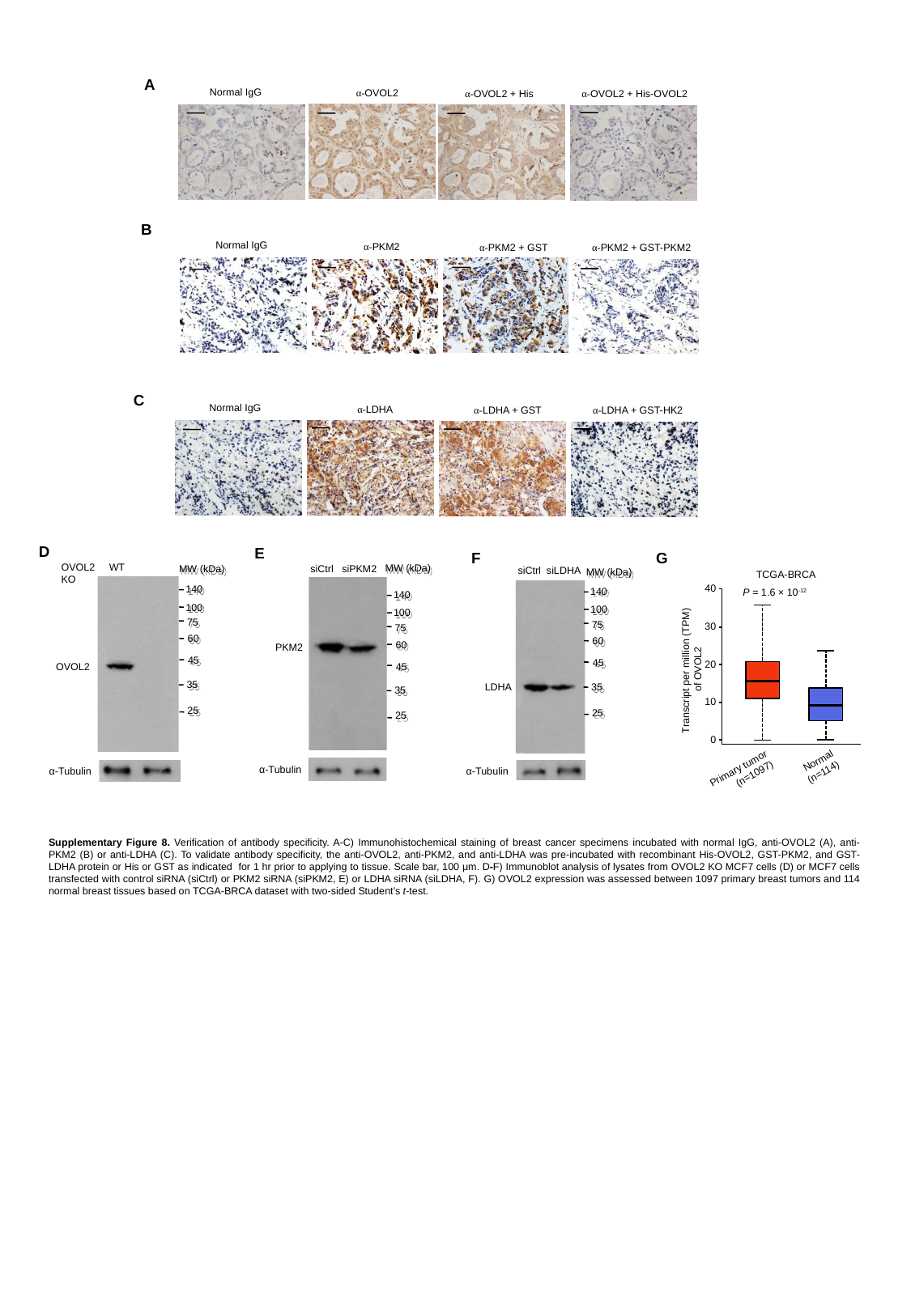

A
Normal IgG
α-OVOL2
α-OVOL2 + His
α-OVOL2 + His-OVOL2
B
Normal IgG
α-PKM2
α-PKM2 + GST
α-PKM2 + GST-PKM2
C
Normal IgG
α-LDHA
α-LDHA + GST
α-LDHA + GST-HK2
D
E
G
F
OVOL2 WT KO
MW (kDa)
siCtrl siPKM2
MW (kDa)
siCtrl siLDHA
MW (kDa)
TCGA-BRCA
40
P = 1.6 × 10^-12^
30
20
Transcript per million (TPM)
 of OVOL2
10
0
Primary tumor
 (n=1097)
Normal
(n=114)
140
140
140
100
100
100
75
75
75
60
60
60
PKM2
45
45
45
OVOL2
35
LDHA
35
35
25
25
25
α-Tubulin
α-Tubulin
α-Tubulin
Supplementary Figure 8. Verification of antibody specificity. A-C) Immunohistochemical staining of breast cancer specimens incubated with normal IgG, anti-OVOL2 (A), anti-PKM2 (B) or anti-LDHA (C). To validate antibody specificity, the anti-OVOL2, anti-PKM2, and anti-LDHA was pre-incubated with recombinant His-OVOL2, GST-PKM2, and GST-LDHA protein or His or GST as indicated for 1 hr prior to applying to tissue. Scale bar, 100 μm. D-F) Immunoblot analysis of lysates from OVOL2 KO MCF7 cells (D) or MCF7 cells transfected with control siRNA (siCtrl) or PKM2 siRNA (siPKM2, E) or LDHA siRNA (siLDHA, F). G) OVOL2 expression was assessed between 1097 primary breast tumors and 114 normal breast tissues based on TCGA-BRCA dataset with two-sided Student’s t-test.
