# Supplementary material for: Transcriptional Repression of Aerobic Glycolysis by OVOL2 in Breast Cancer
Source: Adv Sci (Weinh). 2022 Jul 27;9(27):2200705. doi: 10.1002/advs.202200705 (PMC9507357; doi:10.1002/advs.202200705)
Supplement: Supplementary file 4 — Supporting Information [file ADVS-9-2200705-s001.pptx]

## Slide 1
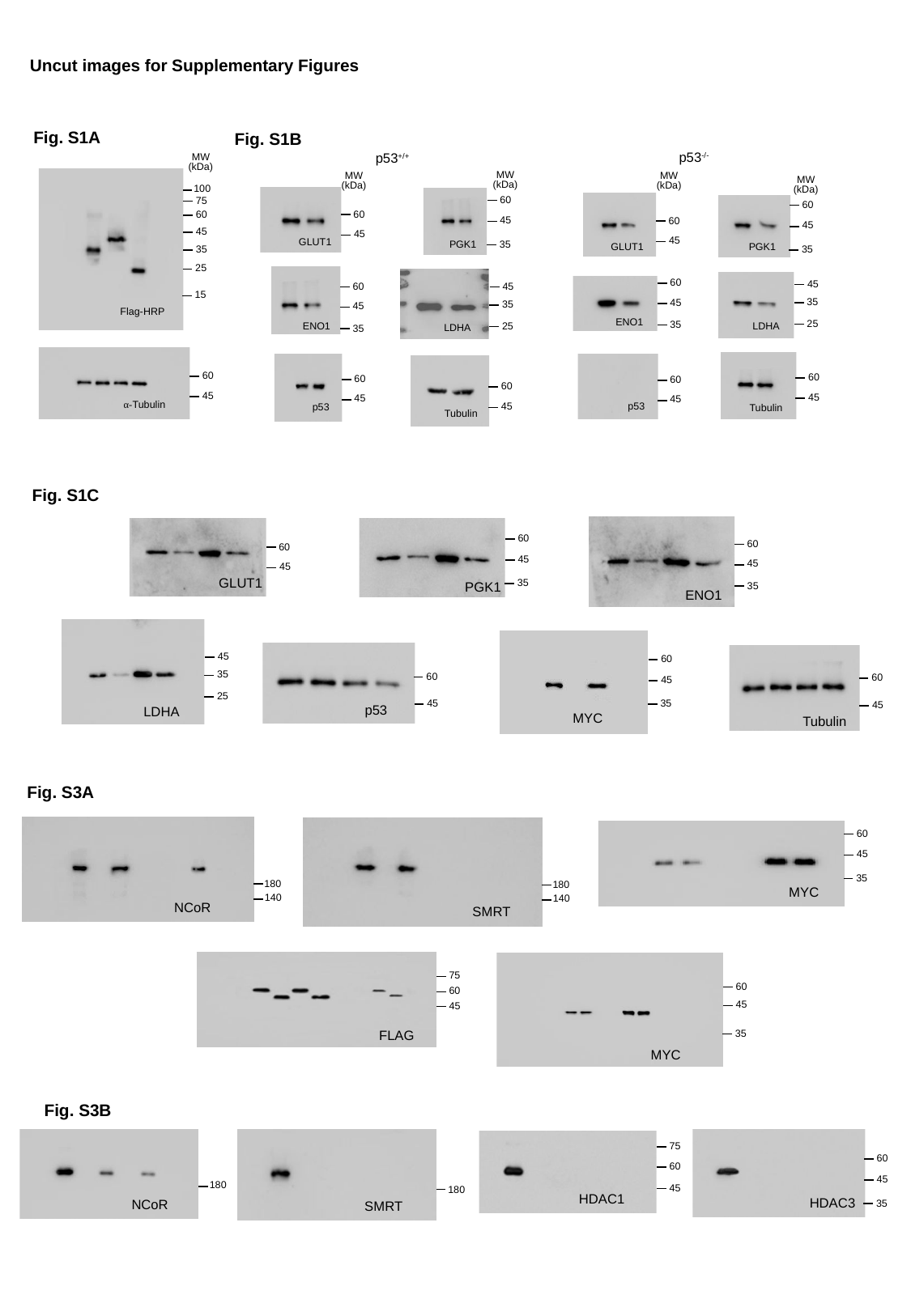

Uncut images for Supplementary Figures
Fig. S1A
Fig. S1B
p53-/-
p53+/+
MW
(kDa)
MW
(kDa)
MW
(kDa)
MW
(kDa)
MW
(kDa)
100
60
75
60
60
60
45
60
45
45
45
45
GLUT1
35
PGK1
GLUT1
PGK1
35
35
25
60
45
45
60
15
35
45
35
45
Flag-HRP
ENO1
25
35
25
ENO1
LDHA
LDHA
35
60
60
60
60
60
45
45
45
45
α-Tubulin
45
p53
p53
Tubulin
Tubulin
Fig. S1C
60
60
60
45
45
45
GLUT1
35
PGK1
35
ENO1
45
60
35
60
60
45
25
45
35
45
p53
LDHA
MYC
Tubulin
Fig. S3A
60
45
35
180
180
MYC
140
140
NCoR
SMRT
75
60
60
45
45
35
FLAG
MYC
Fig. S3B
75
60
60
45
180
45
180
HDAC1
HDAC3
NCoR
35
SMRT

## Slide 2
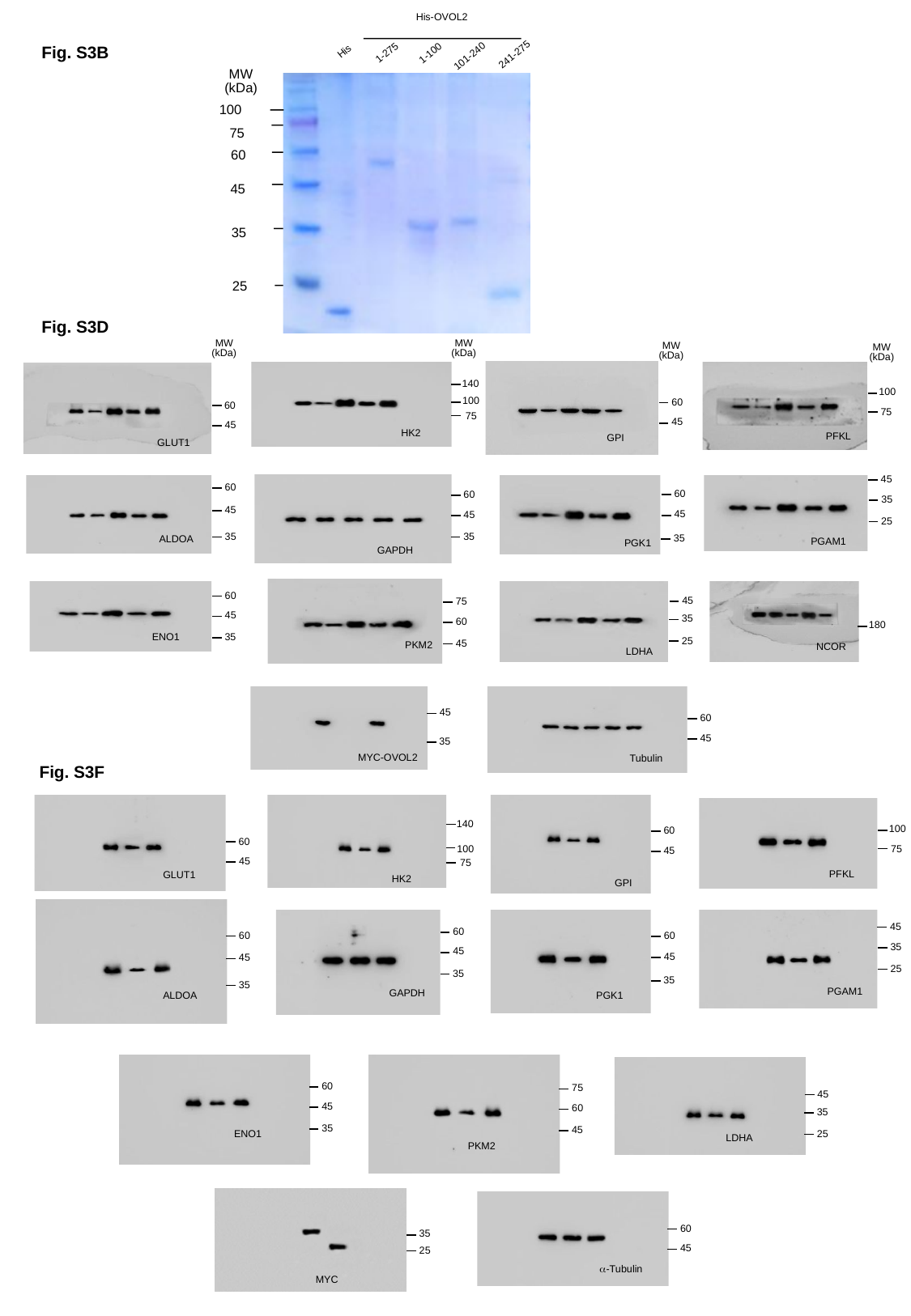

His-OVOL2
His
1-275
1-100
241-275
101-240
100
75
60
45
35
25
Fig. S3B
MW
(kDa)
Fig. S3D
MW
(kDa)
MW
(kDa)
MW
(kDa)
MW
(kDa)
140
100
100
60
60
75
75
45
45
HK2
PFKL
GPI
GLUT1
45
60
60
60
35
45
45
45
25
35
35
35
ALDOA
PGAM1
PGK1
GAPDH
60
45
75
45
35
60
180
35
ENO1
25
45
PKM2
NCOR
LDHA
45
60
45
35
MYC-OVOL2
Tubulin
Fig. S3F
140
100
60
60
75
100
45
45
75
PFKL
GLUT1
HK2
GPI
45
60
60
60
35
45
45
45
25
35
35
35
PGAM1
GAPDH
ALDOA
PGK1
60
75
45
45
60
35
35
45
25
ENO1
LDHA
PKM2
60
35
45
25
a-Tubulin
MYC

## Slide 3
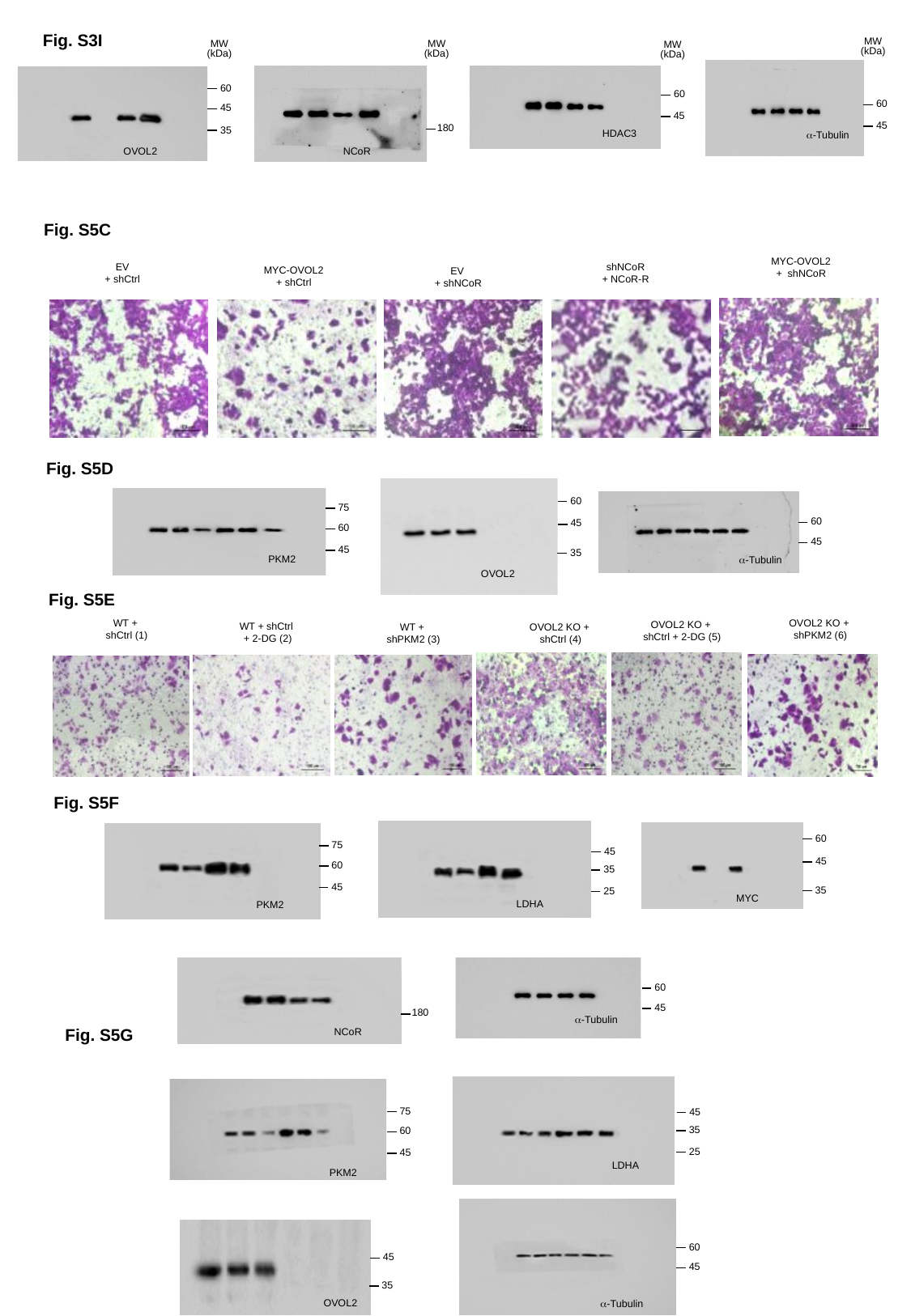

Fig. S3I
MW
(kDa)
MW
(kDa)
MW
(kDa)
MW
(kDa)
60
60
60
45
45
45
180
35
HDAC3
a-Tubulin
OVOL2
NCoR
Fig. S5C
MYC-OVOL2
 + shNCoR
shNCoR
+ NCoR-R
EV
+ shCtrl
MYC-OVOL2
 + shCtrl
EV
 + shNCoR
Fig. S5D
60
75
60
45
60
45
45
35
PKM2
a-Tubulin
OVOL2
Fig. S5E
WT +
shCtrl (1)
OVOL2 KO +
shPKM2 (6)
OVOL2 KO +
shCtrl + 2-DG (5)
WT + shCtrl
 + 2-DG (2)
WT +
shPKM2 (3)
OVOL2 KO +
shCtrl (4)
Fig. S5F
60
75
45
45
60
35
45
35
25
MYC
LDHA
PKM2
60
45
180
a-Tubulin
Fig. S5G
NCoR
75
45
35
60
25
45
LDHA
PKM2
60
45
45
35
OVOL2
a-Tubulin

## Slide 4
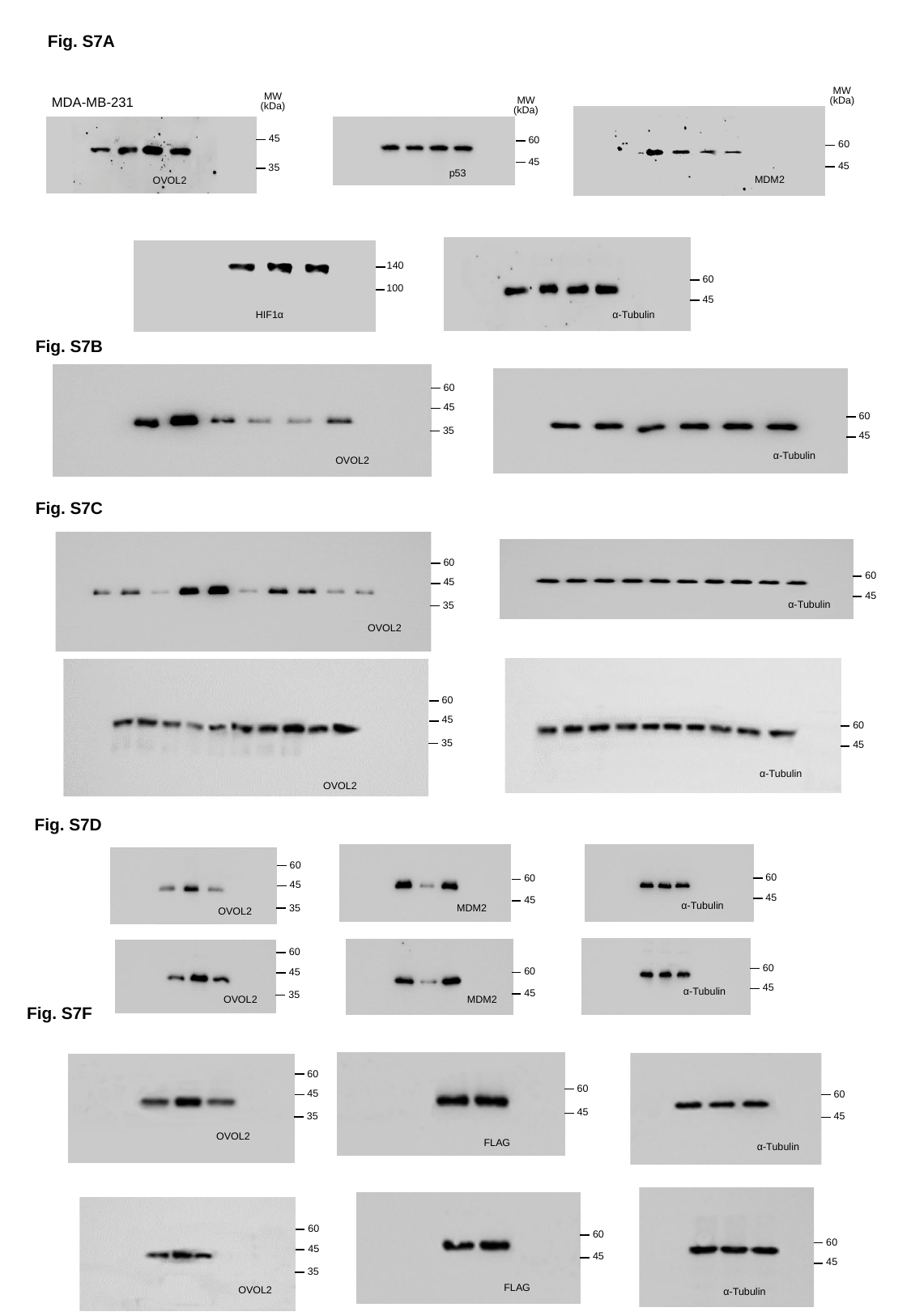

Fig. S7A
MW
(kDa)
MW
(kDa)
MDA-MB-231
MW
(kDa)
45
60
60
45
45
35
p53
MDM2
OVOL2
140
60
100
45
HIF1α
α-Tubulin
Fig. S7B
60
45
60
35
45
α-Tubulin
OVOL2
Fig. S7C
60
60
45
45
α-Tubulin
35
OVOL2
60
45
60
35
45
α-Tubulin
OVOL2
Fig. S7D
60
60
60
45
45
45
α-Tubulin
35
MDM2
OVOL2
60
60
60
45
45
α-Tubulin
45
35
OVOL2
MDM2
Fig. S7F
60
60
45
60
45
45
35
OVOL2
FLAG
α-Tubulin
60
60
60
45
45
45
35
FLAG
OVOL2
α-Tubulin

## Slide 5
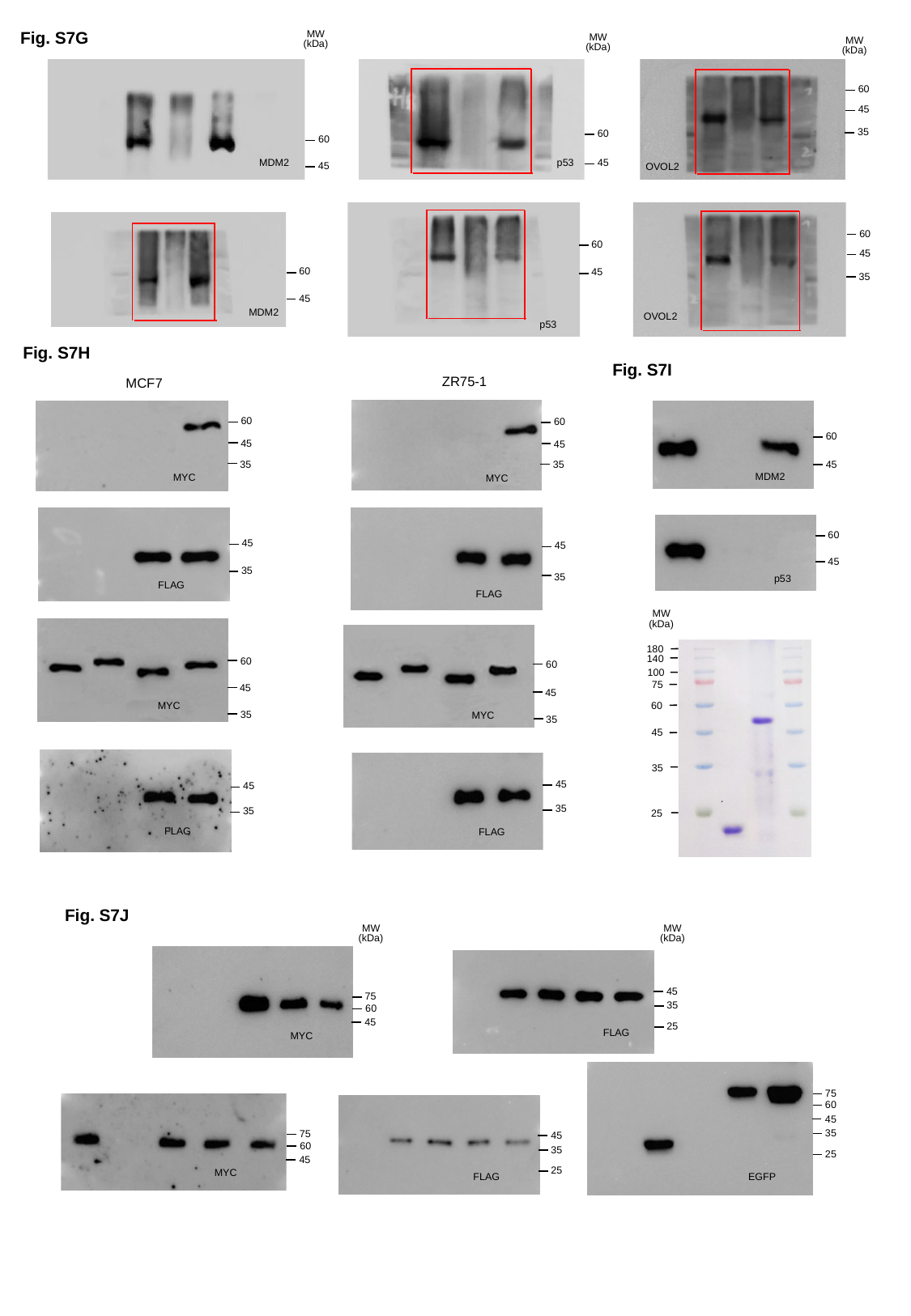

Fig. S7G
MW
(kDa)
MW
(kDa)
MW
(kDa)
60
45
35
60
60
45
MDM2
p53
45
OVOL2
60
60
45
60
45
35
45
MDM2
OVOL2
p53
Fig. S7H
Fig. S7I
ZR75-1
MCF7
60
60
60
45
45
35
45
35
MDM2
MYC
MYC
60
45
45
45
35
35
p53
FLAG
FLAG
MW
(kDa)
180
60
140
60
100
45
75
45
MYC
60
35
MYC
35
45
35
45
45
35
35
25
FLAG
FLAG
Fig. S7J
MW
(kDa)
MW
(kDa)
45
75
35
60
45
25
FLAG
MYC
75
60
45
35
75
45
60
35
25
45
25
MYC
FLAG
EGFP

## Slide 6
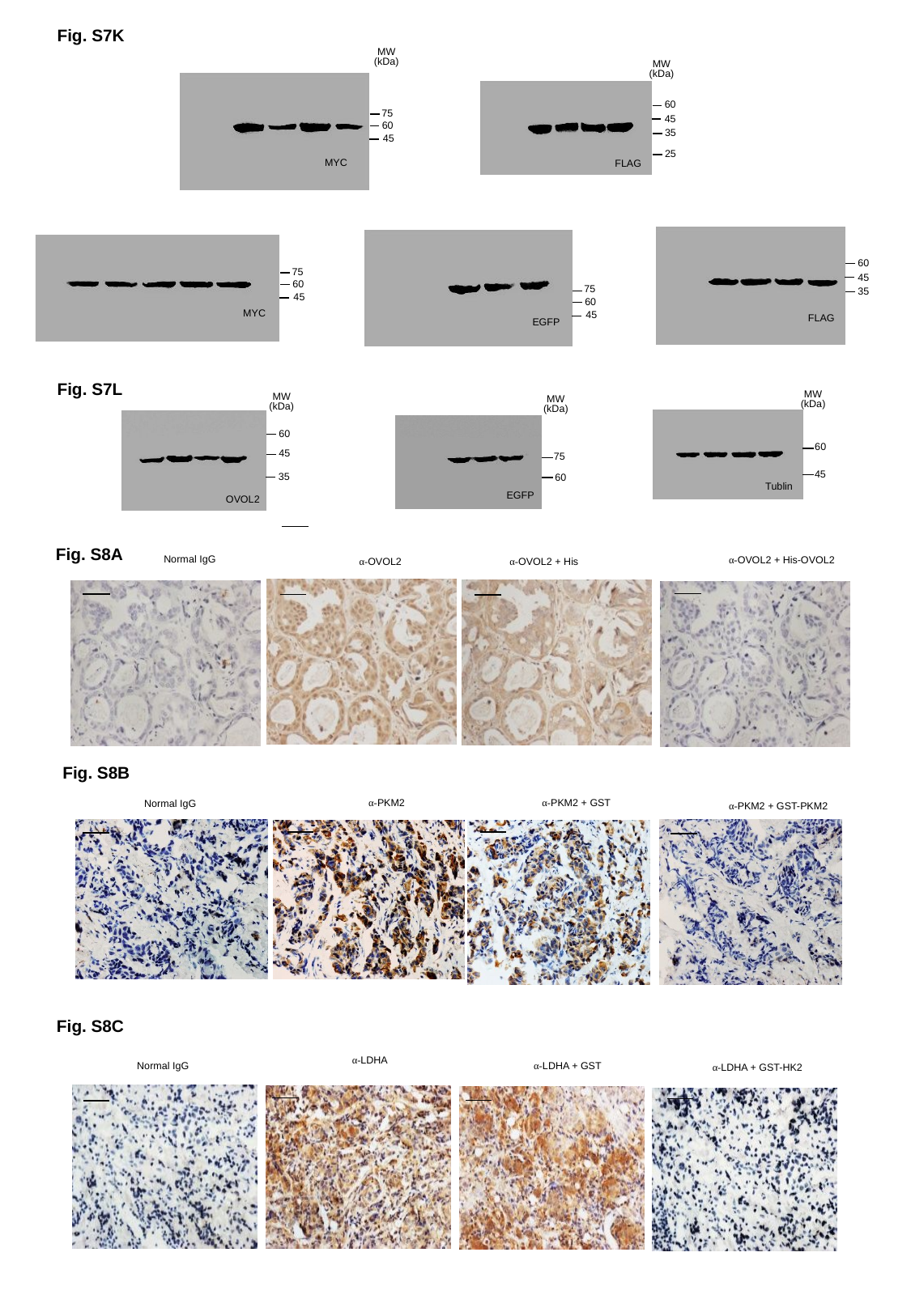

Fig. S7K
MW
(kDa)
MW
(kDa)
60
75
45
60
35
45
25
MYC
FLAG
60
75
45
60
75
35
45
60
MYC
45
FLAG
EGFP
Fig. S7L
MW
(kDa)
MW
(kDa)
MW
(kDa)
60
60
45
75
45
35
60
Tublin
EGFP
OVOL2
Fig. S8A
Normal IgG
α-OVOL2 + His-OVOL2
α-OVOL2
α-OVOL2 + His
Fig. S8B
α-PKM2
α-PKM2 + GST
Normal IgG
α-PKM2 + GST-PKM2
Fig. S8C
α-LDHA
α-LDHA + GST
Normal IgG
α-LDHA + GST-HK2

## Slide 7
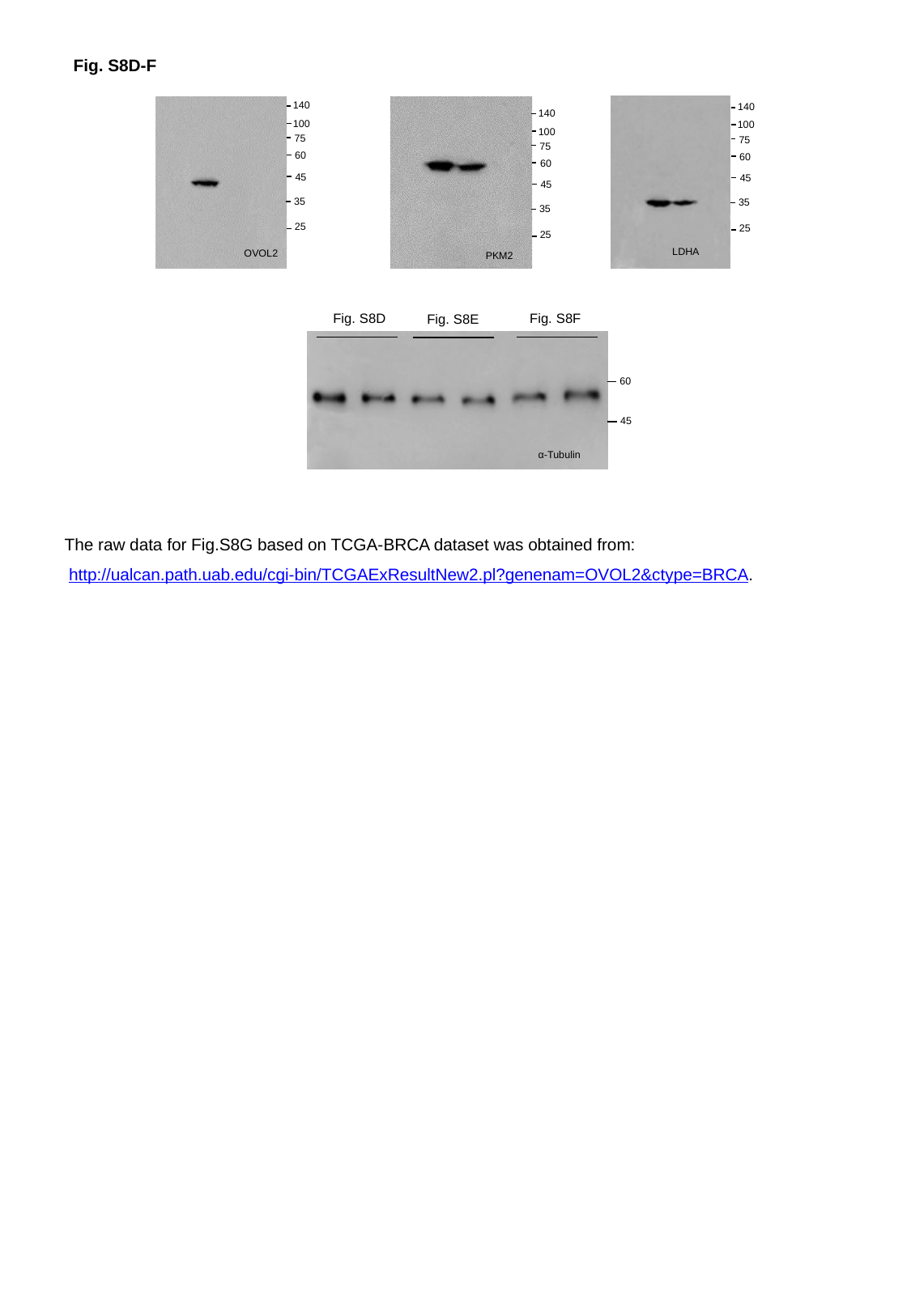

Fig. S8D-F
140
140
140
100
100
100
75
75
75
60
60
60
45
45
45
35
35
35
25
25
25
LDHA
OVOL2
PKM2
Fig. S8F
Fig. S8D
Fig. S8E
60
45
α-Tubulin
The raw data for Fig.S8G based on TCGA-BRCA dataset was obtained from:
 http://ualcan.path.uab.edu/cgi-bin/TCGAExResultNew2.pl?genenam=OVOL2&ctype=BRCA.
