# Supplementary material for: Transcriptional Repression of Aerobic Glycolysis by OVOL2 in Breast Cancer
Source: Adv Sci (Weinh). 2022 Jul 27;9(27):2200705. doi: 10.1002/advs.202200705 (PMC9507357; doi:10.1002/advs.202200705)
Supplement: Supplementary file 5 — Supporting Information [file ADVS-9-2200705-s003.pptx]

## Slide 1
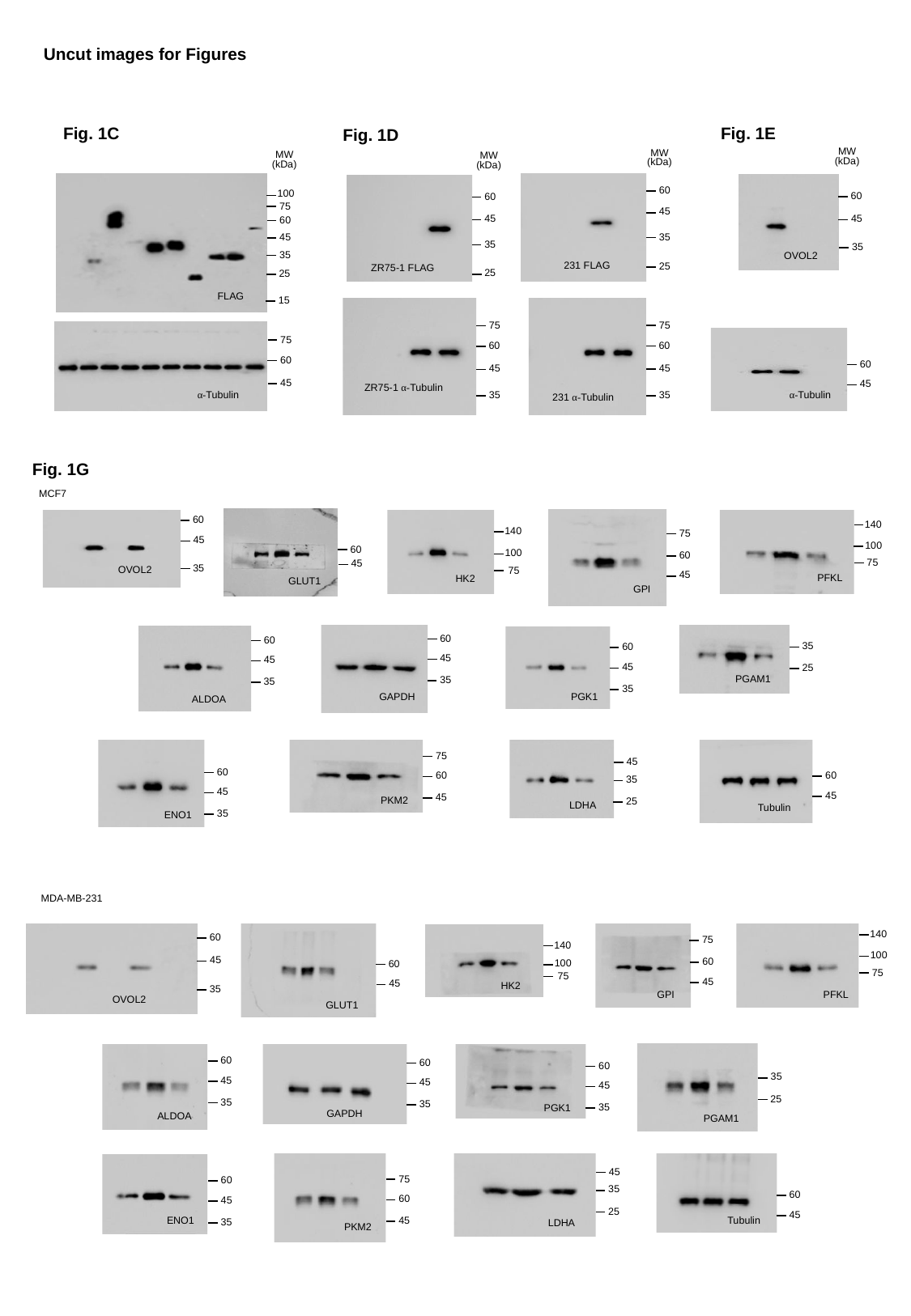

Uncut images for Figures
Fig. 1C
Fig. 1E
Fig. 1D
MW
(kDa)
MW
(kDa)
MW
(kDa)
MW
(kDa)
60
100
60
60
75
45
45
45
60
45
35
35
35
35
OVOL2
231 FLAG
25
ZR75-1 FLAG
25
25
FLAG
15
75
75
75
60
60
60
60
45
45
45
45
ZR75-1 α-Tubulin
α-Tubulin
35
α-Tubulin
35
231 α-Tubulin
Fig. 1G
MCF7
60
140
140
75
45
100
60
100
60
75
45
35
OVOL2
75
45
PFKL
HK2
GLUT1
GPI
60
60
35
60
45
45
45
25
PGAM1
35
35
35
PGK1
GAPDH
ALDOA
75
45
60
60
60
35
45
45
45
PKM2
25
LDHA
Tubulin
35
ENO1
MDA-MB-231
140
60
75
140
100
45
60
100
60
75
75
45
45
HK2
35
GPI
PFKL
OVOL2
GLUT1
60
60
60
35
45
45
45
25
35
35
35
PGK1
GAPDH
ALDOA
PGAM1
45
75
60
35
60
60
45
25
45
Tubulin
45
ENO1
35
LDHA
PKM2

## Slide 2
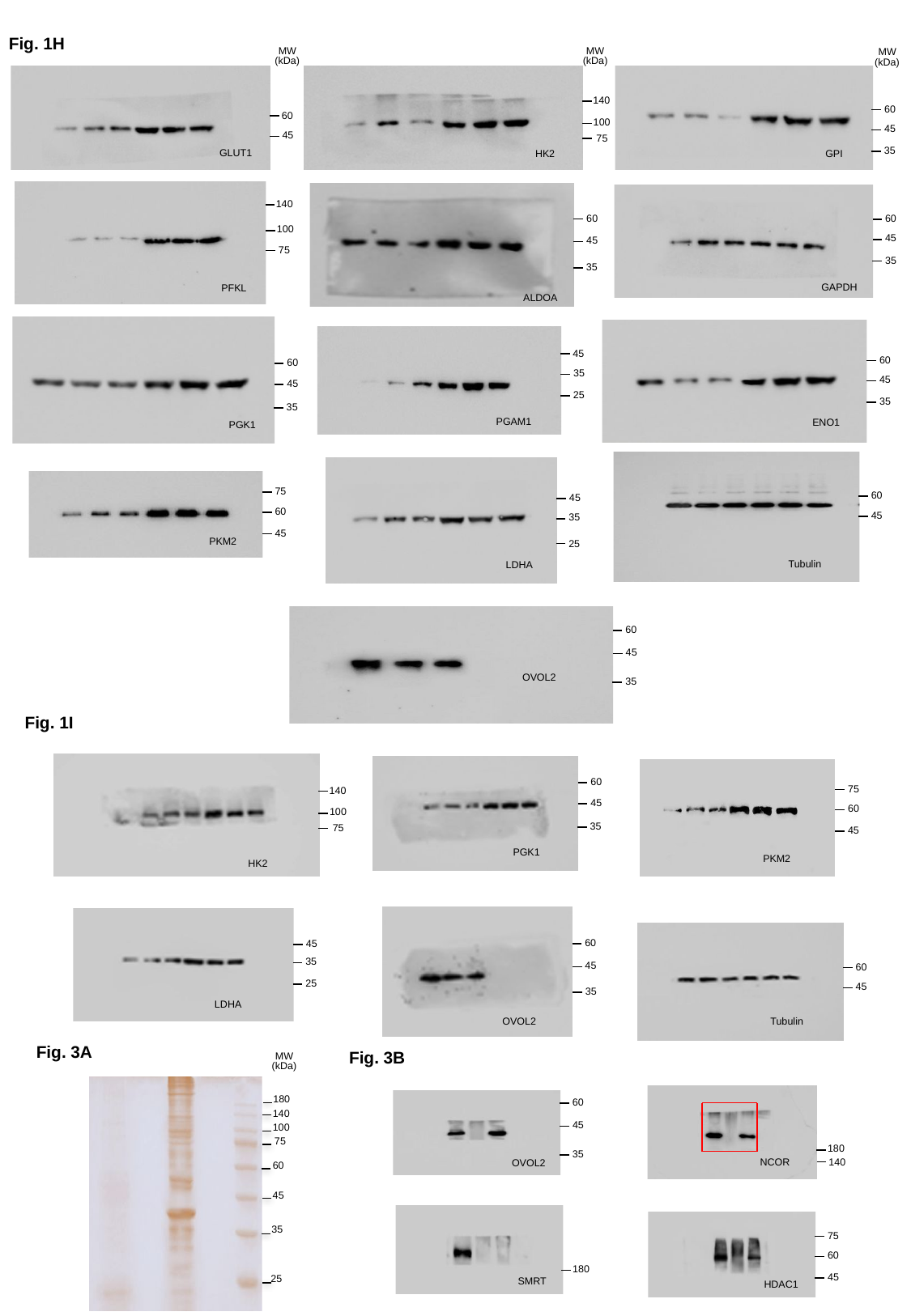

Fig. 1H
MW
(kDa)
MW
(kDa)
MW
(kDa)
140
60
60
100
45
45
75
35
GLUT1
HK2
GPI
140
60
60
100
45
45
75
35
35
GAPDH
PFKL
ALDOA
45
60
60
35
45
45
25
35
35
PGAM1
ENO1
PGK1
75
60
45
60
45
35
45
PKM2
25
Tubulin
LDHA
60
45
OVOL2
35
Fig. 1I
60
75
140
45
60
100
35
75
45
PGK1
PKM2
HK2
60
45
35
45
60
25
45
35
LDHA
Tubulin
OVOL2
Fig. 3A
Fig. 3B
MW
(kDa)
180
60
140
45
100
75
180
35
140
NCOR
OVOL2
60
45
35
75
60
180
45
25
SMRT
HDAC1

## Slide 3
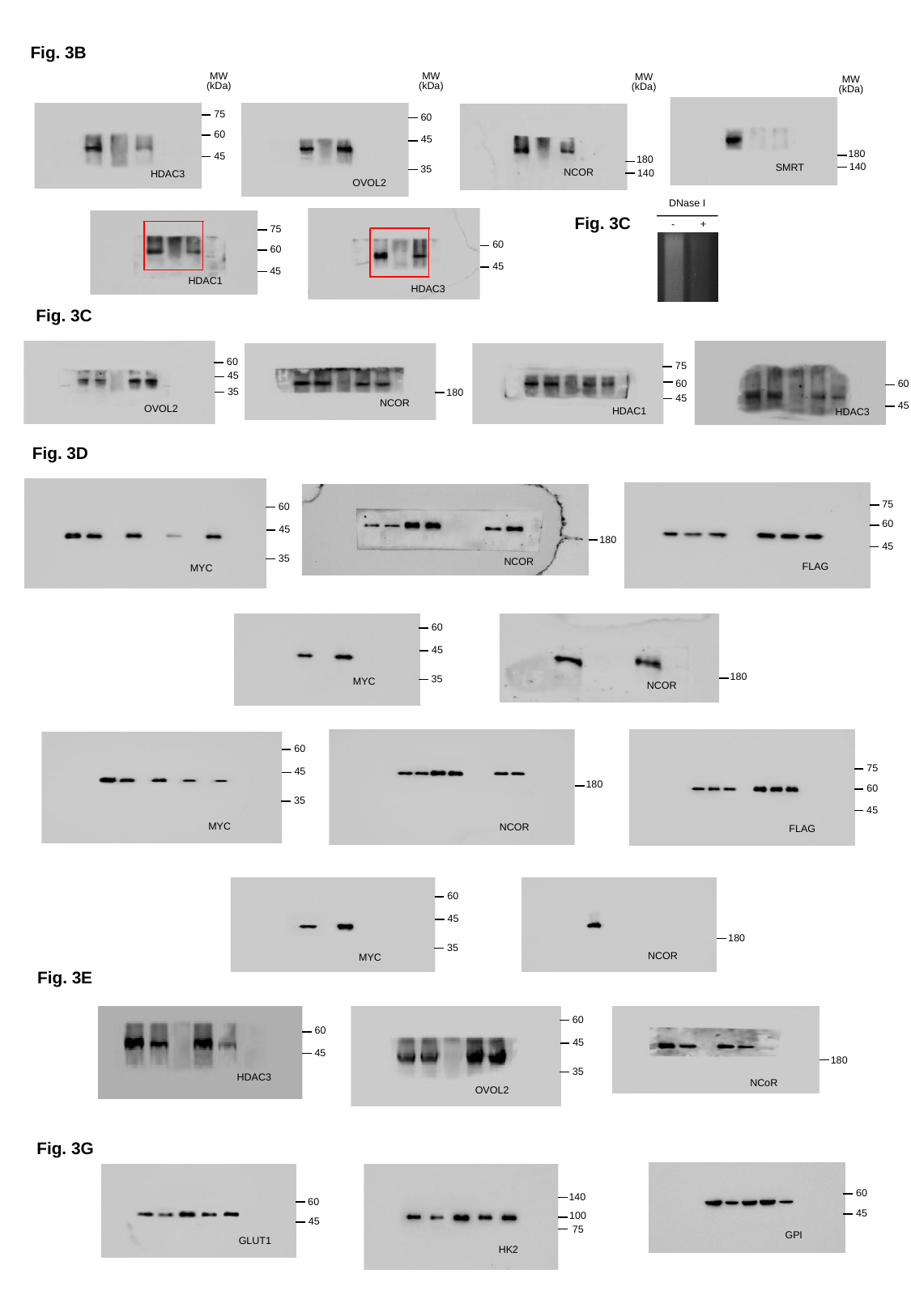

Fig. 3B
MW
(kDa)
MW
(kDa)
MW
(kDa)
MW
(kDa)
75
60
60
45
180
45
180
140
SMRT
35
NCOR
140
HDAC3
OVOL2
DNase I
Fig. 3C
- +
75
60
60
45
45
HDAC1
HDAC3
Fig. 3C
60
75
45
60
60
35
180
45
NCOR
45
OVOL2
HDAC1
HDAC3
Fig. 3D
75
60
60
45
180
45
35
NCOR
FLAG
MYC
60
45
180
35
MYC
NCOR
60
75
45
180
60
35
45
MYC
NCOR
FLAG
60
45
180
35
NCOR
MYC
Fig. 3E
60
60
45
45
180
35
HDAC3
NCoR
OVOL2
Fig. 3G
60
140
60
45
100
45
75
GPI
GLUT1
HK2

## Slide 4
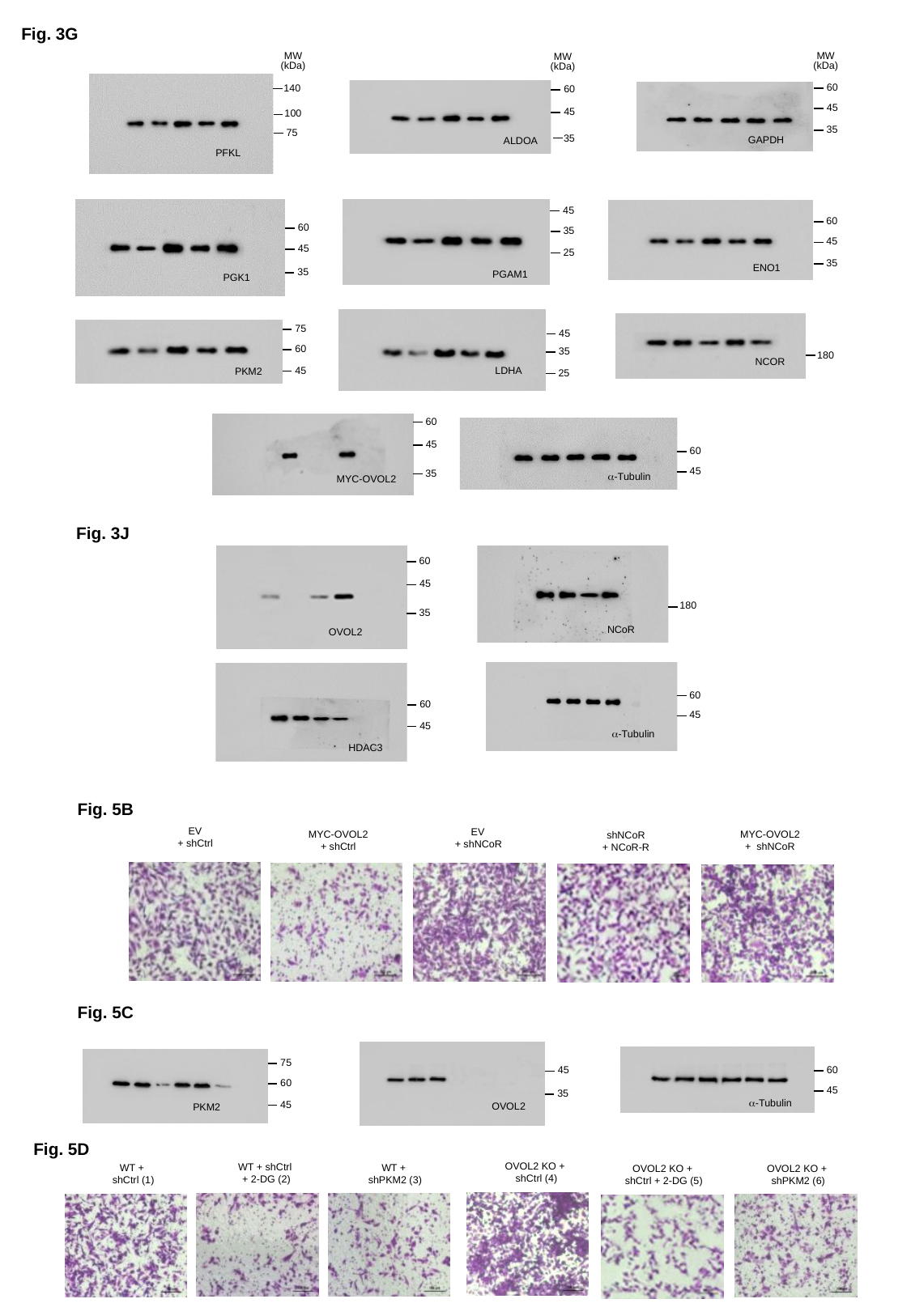

Fig. 3G
MW
(kDa)
MW
(kDa)
MW
(kDa)
60
140
60
45
45
100
35
75
35
GAPDH
ALDOA
PFKL
45
60
60
35
45
45
25
35
ENO1
35
PGAM1
PGK1
75
45
60
35
180
NCOR
45
LDHA
PKM2
25
60
45
60
45
35
a-Tubulin
MYC-OVOL2
Fig. 3J
60
45
180
35
NCoR
OVOL2
60
60
45
45
a-Tubulin
HDAC3
Fig. 5B
EV
+ shCtrl
EV
 + shNCoR
MYC-OVOL2
 + shCtrl
MYC-OVOL2
 + shNCoR
shNCoR
+ NCoR-R
Fig. 5C
75
45
60
60
45
35
a-Tubulin
45
OVOL2
PKM2
Fig. 5D
OVOL2 KO +
shCtrl (4)
WT + shCtrl
 + 2-DG (2)
WT +
shPKM2 (3)
WT +
shCtrl (1)
OVOL2 KO +
shCtrl + 2-DG (5)
OVOL2 KO +
shPKM2 (6)

## Slide 5
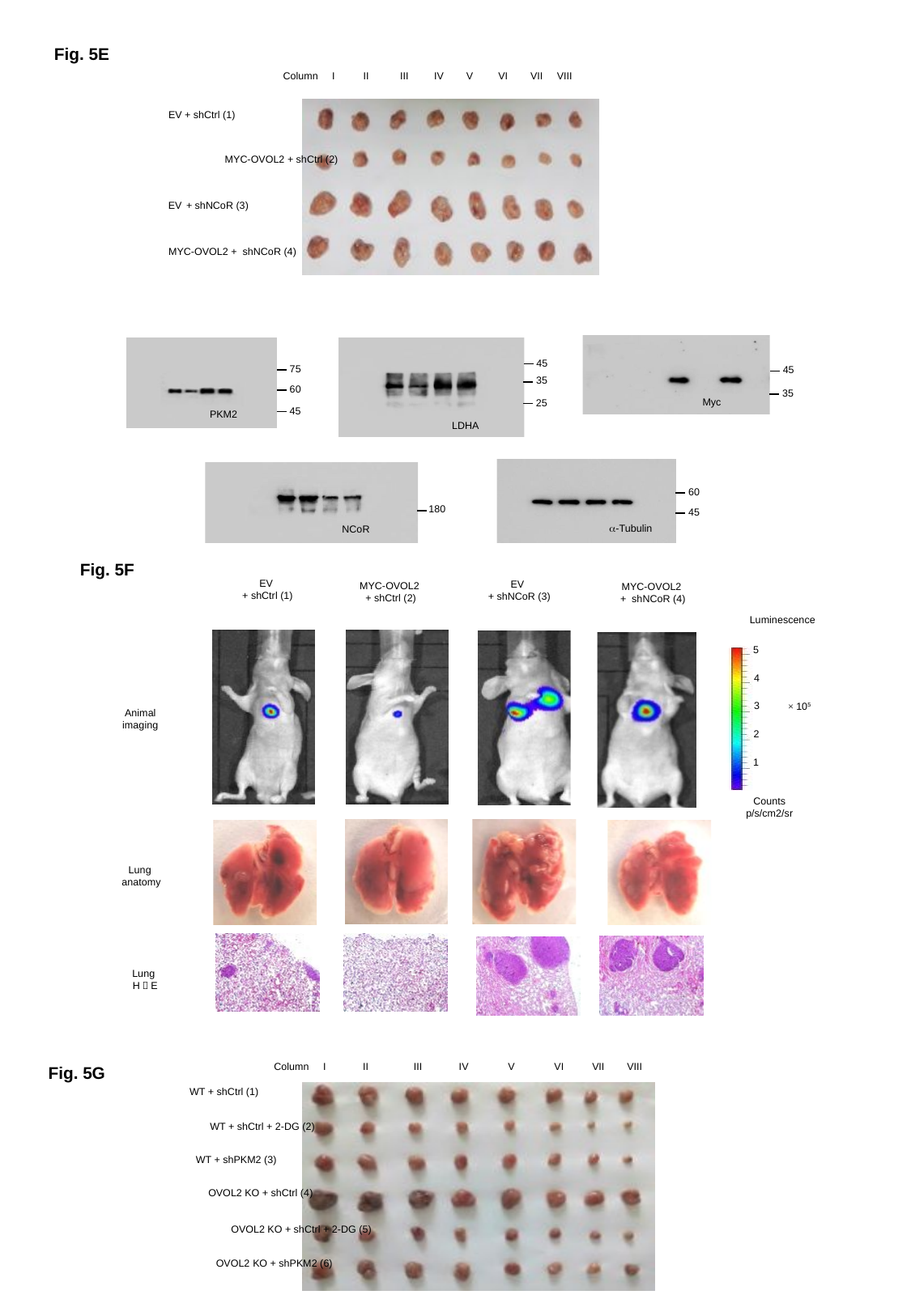

Fig. 5E
Column I II III IV V VI VII VIII
EV + shCtrl (1)
MYC-OVOL2 + shCtrl (2)
EV + shNCoR (3)
MYC-OVOL2 + shNCoR (4)
45
75
45
35
60
35
Myc
25
45
PKM2
LDHA
60
180
45
a-Tubulin
NCoR
Fig. 5F
EV
+ shCtrl (1)
EV
 + shNCoR (3)
MYC-OVOL2
 + shCtrl (2)
MYC-OVOL2
 + shNCoR (4)
Luminescence
5
4
3
× 105
2
1
Counts
p/s/cm2/sr
Animal
imaging
Lung
 anatomy
Lung
 H＆E
Fig. 5G
Column I II III IV V VI VII VIII
WT + shCtrl (1)
WT + shCtrl + 2-DG (2)
WT + shPKM2 (3)
OVOL2 KO + shCtrl (4)
OVOL2 KO + shCtrl + 2-DG (5)
OVOL2 KO + shPKM2 (6)

## Slide 6
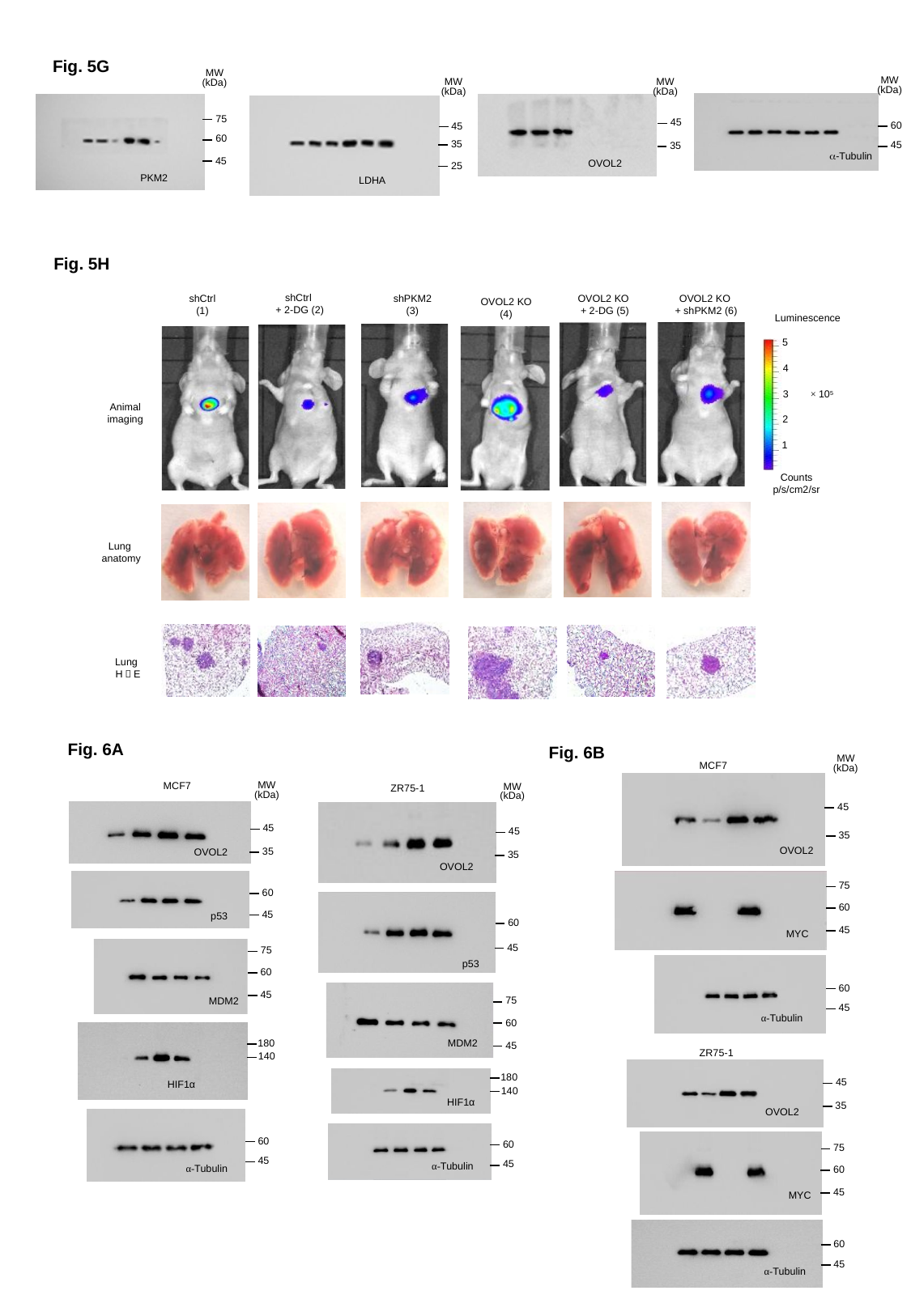

Fig. 5G
MW
(kDa)
MW
(kDa)
MW
(kDa)
MW
(kDa)
75
45
60
45
60
35
45
35
a-Tubulin
45
OVOL2
25
PKM2
LDHA
Fig. 5H
shCtrl
 + 2-DG (2)
shCtrl
(1)
shPKM2
(3)
OVOL2 KO
 + 2-DG (5)
OVOL2 KO
 + shPKM2 (6)
OVOL2 KO
(4)
Luminescence
5
4
3
× 105
2
1
Counts
p/s/cm2/sr
Animal
imaging
Lung
 anatomy
Lung
 H＆E
Fig. 6A
Fig. 6B
MW
(kDa)
MCF7
MCF7
MW
(kDa)
ZR75-1
MW
(kDa)
45
45
45
35
OVOL2
35
OVOL2
35
OVOL2
75
60
60
45
p53
60
45
MYC
45
75
p53
60
60
45
75
MDM2
45
α-Tubulin
60
MDM2
180
45
ZR75-1
140
180
45
HIF1α
140
HIF1α
35
OVOL2
60
60
75
45
45
α-Tubulin
α-Tubulin
60
45
MYC
60
45
α-Tubulin

## Slide 7
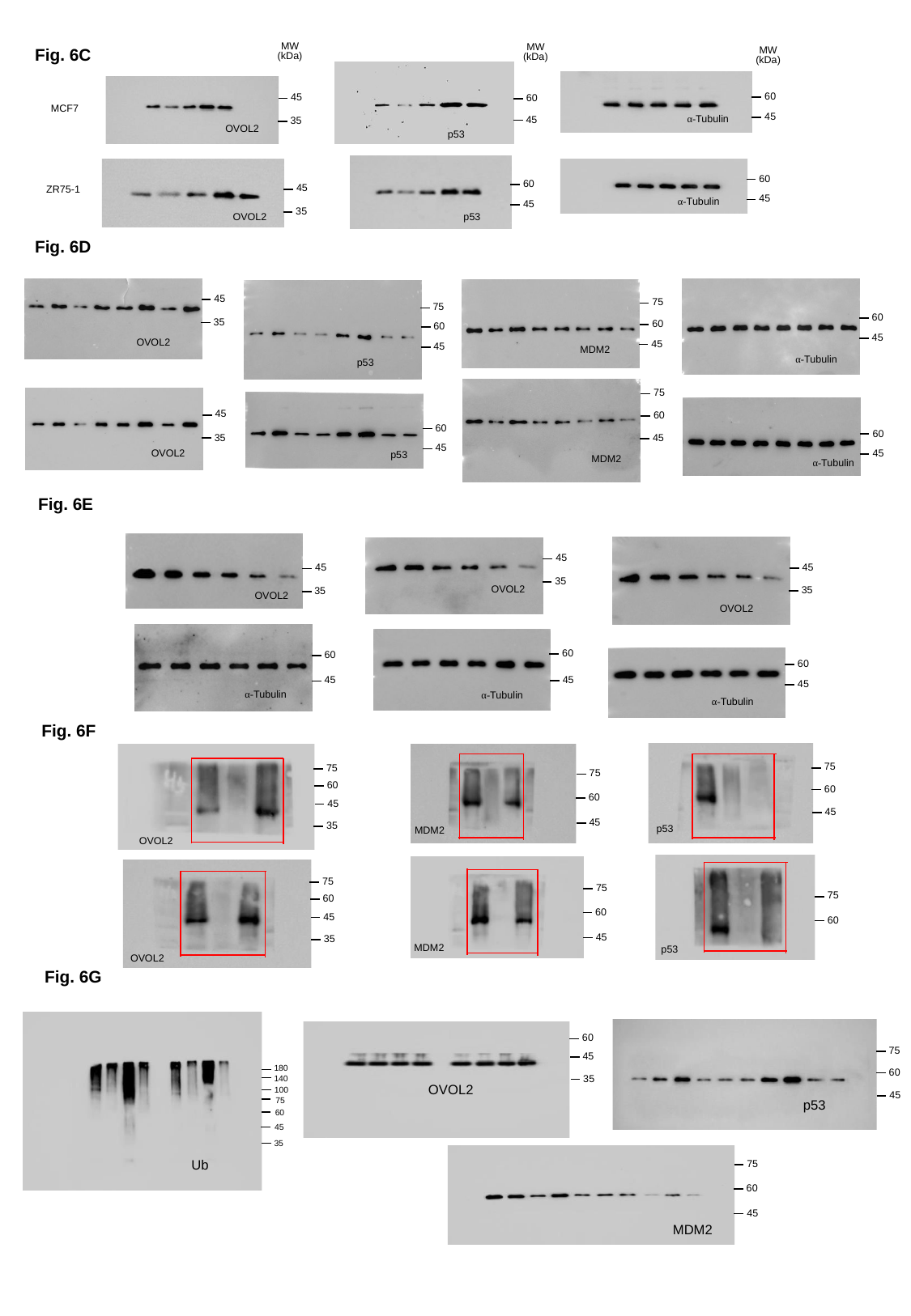

MW
(kDa)
MW
(kDa)
Fig. 6C
MW
(kDa)
60
45
60
MCF7
45
α-Tubulin
45
35
OVOL2
p53
60
60
45
ZR75-1
45
α-Tubulin
45
35
OVOL2
p53
Fig. 6D
45
75
75
60
35
60
60
45
OVOL2
45
45
MDM2
α-Tubulin
p53
75
45
60
60
60
35
45
45
OVOL2
45
p53
MDM2
α-Tubulin
Fig. 6E
45
45
45
35
OVOL2
35
35
OVOL2
OVOL2
60
60
60
45
45
45
α-Tubulin
α-Tubulin
α-Tubulin
Fig. 6F
75
75
75
60
60
60
45
45
45
35
p53
MDM2
OVOL2
75
75
75
60
60
45
60
45
35
MDM2
p53
OVOL2
Fig. 6G
60
75
45
180
60
35
140
OVOL2
100
45
75
p53
60
45
35
Ub
75
60
45
MDM2

## Slide 8
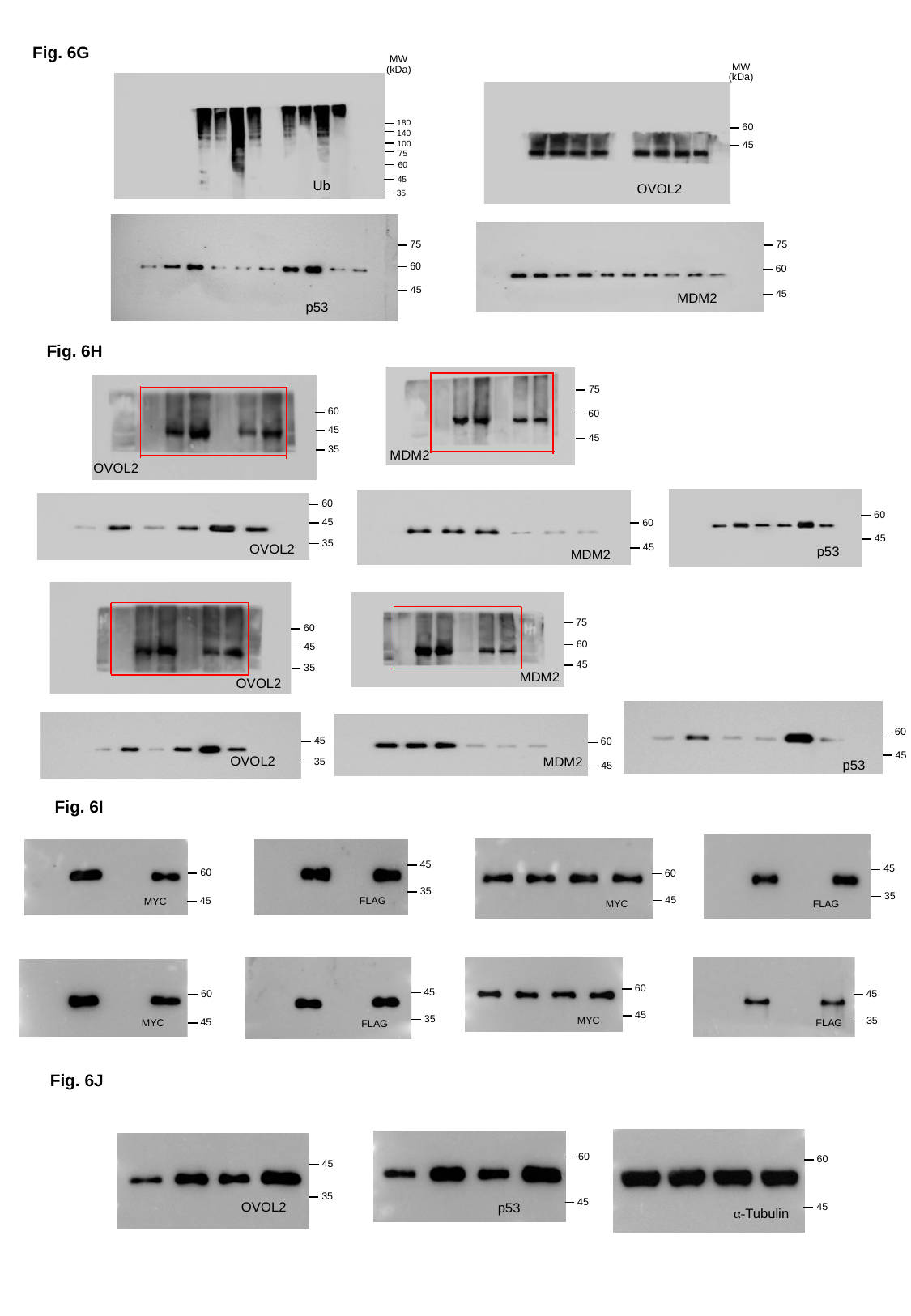

Fig. 6G
MW
(kDa)
MW
(kDa)
180
60
140
100
45
75
60
45
Ub
OVOL2
35
75
75
60
60
45
45
MDM2
p53
Fig. 6H
75
60
60
45
45
35
MDM2
OVOL2
60
60
45
60
45
35
OVOL2
45
p53
MDM2
75
60
60
45
45
35
MDM2
OVOL2
60
45
60
45
OVOL2
MDM2
35
p53
45
Fig. 6I
45
45
60
60
35
35
45
FLAG
45
MYC
FLAG
MYC
60
45
45
60
45
35
MYC
35
45
FLAG
MYC
FLAG
Fig. 6J
60
60
45
35
45
OVOL2
p53
45
α-Tubulin

## Slide 9
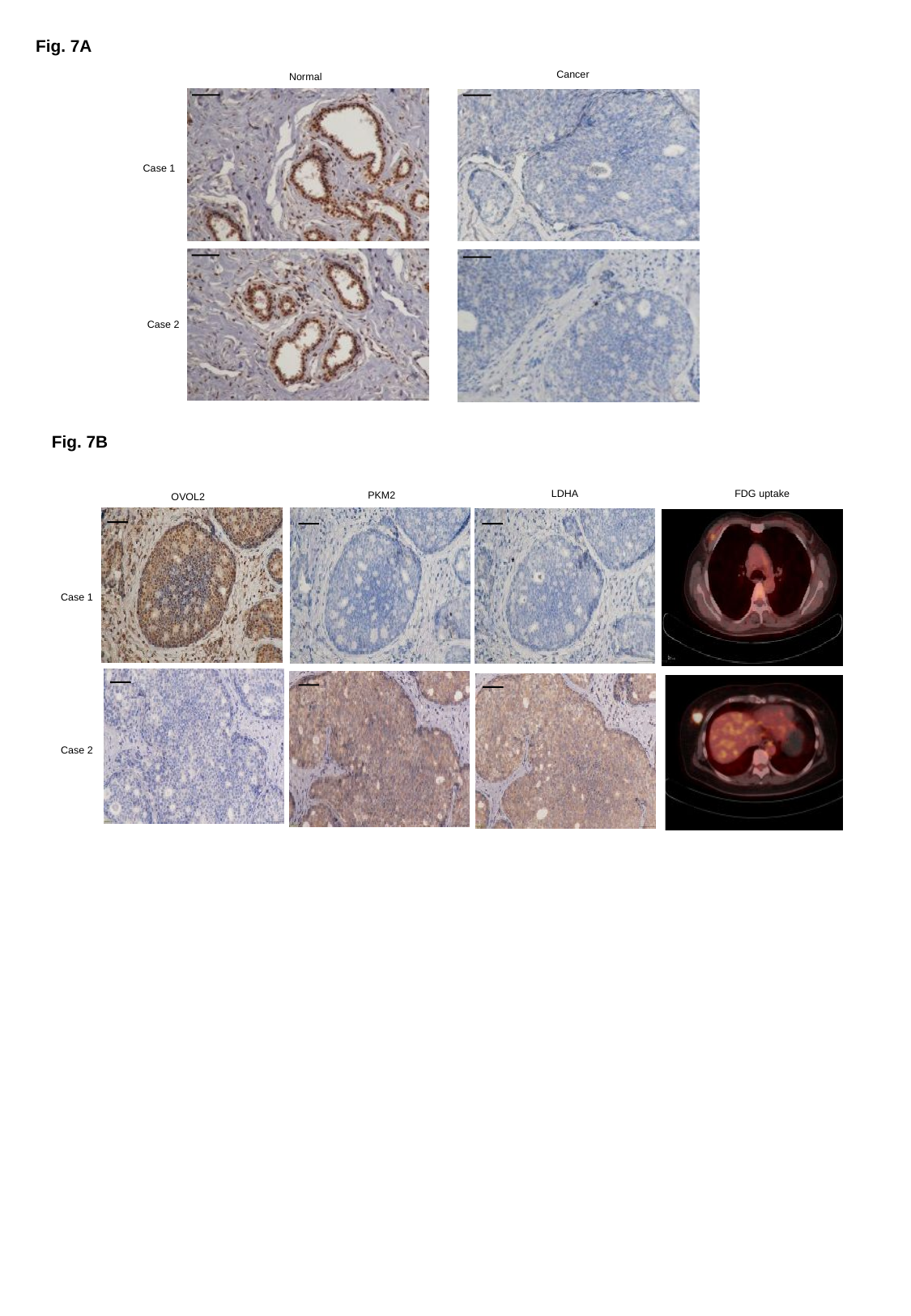

Fig. 7A
Cancer
Normal
Case 1
Case 2
Fig. 7B
LDHA
FDG uptake
PKM2
OVOL2
Case 1
Case 2
